# New Heterocyclic Combretastatin A-4 Analogs: Synthesis and Biological Activity of Styryl-2(3*H*)-benzothiazolones

**DOI:** 10.3390/ph14121331

**Published:** 2021-12-20

**Authors:** Gjorgji Atanasov, Rusi I. Rusew, Vladimir M. Gelev, Christo D. Chanev, Rosica Nikolova, Boris L. Shivachev, Ognyan I. Petrov, Margarita D. Apostolova

**Affiliations:** 1 Roumen Tsanev Institute of Molecular Biology, Bulgarian Academy of Sciences, Acad. G. Bonchev Str., Bl. 21, 1113 Sofia, Bulgaria; atanasovg@bio21.bas.bg; 2 Institute of Mineralogy and Crystallography, Bulgarian Academy of Sciences, Acad. G. Bonchev Str., Bl. 107, 1113 Sofia, Bulgaria; r.rusev93@gmail.com (R.I.R.); rosica.pn@clmc.bas.bg (R.N.); blshivachev@gmail.com (B.L.S.); 3 Department of Pharmaceutical and Applied Organic Chemistry, Faculty of Chemistry and Pharmacy, Sofia University St. Kliment Ohridski, 1 James Bourchier Blvd., 1164 Sofia, Bulgaria; doktor.gelev@gmail.com (V.M.G.); hchanev@chem.uni-sofia.bg (C.D.C.)

**Keywords:** combretastatin A-4, stilbene, benzothiazolones, anticancer agents, tubulin binding, endothelial

## Abstract

Here, we describe the synthesis, characterization, and biological activities of a series of 26 new styryl-2(3H)-benzothiazolone analogs of combretastatin-A4 (CA-4). The cytotoxic activities of these compounds were tested in several cell lines (EA.hy926, A549, BEAS-2B, MDA-MB-231, HT-29, MCF-7, and MCF-10A), and the relations between structure and cytotoxicity are discussed. From the series, compound (*Z*)-3-methyl-6-(3,4,5-trimethoxystyryl)-2(3*H*)-benzothiazolone (**26Z**) exhibits the most potent cytotoxic activity (IC_50_ 0.13 ± 0.01 µM) against EA.hy926 cells. **26Z** not only inhibits vasculogenesis but also disrupts pre-existing vasculature. **26Z** is a microtubule-modulating agent and inhibits a spectrum of angiogenic events in EA.hy926 cells by interfering with endothelial cell invasion, migration, and proliferation. **26Z** also shows anti-proliferative activity in CA-4 resistant cells with the following IC_50_ values: HT-29 (0.008 ± 0.001 µM), MDA-MB-231 (1.35 ± 0.42 µM), and MCF-7 (2.42 ± 0.48 µM). Cell-cycle phase-specific experiments show that **26Z** treatment results in G2/M arrest and mitotic spindle multipolarity, suggesting that drug-induced centrosome amplification could promote cell death. Some **26Z**-treated adherent cells undergo aberrant cytokinesis, resulting in aneuploidy that perhaps contributes to drug-induced cell death. These data indicate that spindle multipolarity induction by **26Z** has an exciting chemotherapeutic potential that merits further investigation.

## 1. Introduction

**Figure 1:**
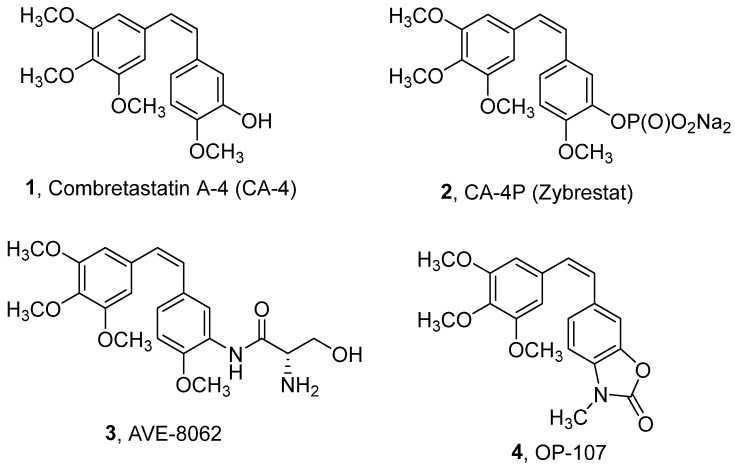
Structure of natural CA-4 and its synthetic analogs.

Microtubules are intracellular polymers comprised of α,β-tubulin heterodimers. They have essential cellular functions such as cell shape maintenance, organelle distribution, cell motility, and chromosome segregation [1]. Disruption of microtubule formation leads to G2/M phase cell cycle arrest and apoptosis [2]. Compounds that disrupt microtubule polymerization/depolymerization dynamics have been extensively studied as anticancer agents [2,3,4]. Such compounds include vinblastine, vincristine, and vinorelbine, which bind to tubulin’s vinca domain, while docetaxel and paclitaxel bind to the taxane-site. [5]. Combretastatin A-4 (CA-4) binds to the colchicine binding site of tubulin. Originally isolated from the bark of the South African willow tree *Combretum caffrum* [6], CA-4 is one of the most potent inhibitors of tubulin polymerization, which interferes with microtubule dynamics and perturbs the mitotic cycle [7,8]. CA-4 selectively inhibits tumor neovascularization, causing rapid vascular shutdown and tumor necrosis [9,10]. When administered in vivo, CA-4 causes disruption and collapse of tumor blood vessels and subsequent necrotic cell death of tumor cells due to the lack of oxygen and nutrients [11,12,13]. The clinical usefulness of CA-4 and other vascular disrupting drugs is hampered by the formation of a viable tumor rim and by dose-limiting adverse effects, such as cardiotoxicity [14,15]. In addition, CA-4 has poor water solubility and is chemically unstable, with the active cis-isomer spontaneously converting to the thermodynamically favorable but less potent trans-isomer [16]. Numerous analogs of CA-4 have been tested to address these drawbacks [17,18,19,20]. The water-soluble phosphate prodrugs Zybrestat (**2**) and AVE-8082 (**3**) have shown promising results in pre-clinical and clinical studies, particularly in combination with other chemotherapeutic agents [21,22] against thyroid, non-small cell lung, and ovarian cancers [23,24,25]. We previously evaluated several CA-4 analogs possessing a benzoxazolone scaffold, and we identified (*Z*)-3-methyl-6-(3,4,5-trimethoxystyryl)-2(3*H*)-benzoxazolone (**4**, OP-107, Figure 1) as being quite potent in inducing anti-proliferative/cytotoxic and anti-angiogenic effects [19].

**Scheme 1:**
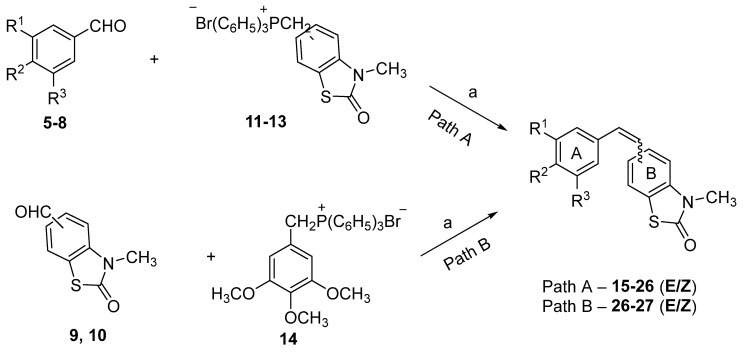
Synthesis of target styryl-benzothiazolones. Reagents and conditions: (**a**) K_2_CO_3_, 18-crown-6, THF/CH_2_Cl_2_ (2:1 *v*/*v*), reflux 4–6 h, 57–87%.

Here, we report a series of 26 new styryl-2(3*H*)-benzothiazolone analogs of CA-4 (Scheme 1, compounds **15**–**27** (**E/Z**)). The 2(3*H*)-benzothiazolone moiety was chosen because of the interesting synthetic and antitumor properties and cardiotonic activity of several compounds containing this moiety [26,27,28]. The detailed physicochemical characterization and the biological activity of 26 new CA-4 benzothiazolone analogs are presented here.

## 2. Results and Discussion

### 2.1. Chemistry

There are numerous methods for producing stilbenes, including synthesis by Wittig reaction [29], Perkin condensation [30], Suzuki coupling [31], Kumada–Corriu cross-coupling [32], and Colvin rearrangement [33]. The Wittig reaction is arguably the most thoroughly studied and direct route to *E/Z*-stilbenes from easily accessible starting materials. The synthesis of the target compounds from substituted methoxybenzaldehydes **5**–**8** and the phosphonium salts **11**–**13** (Path A), or 3-methyl-2(3*H*)-benzothiazolone-6-carbaldehyde or 3-methyl-2(3*H*)-benzothiazolone-7-carbaldehyde **9**–**10** and the phosphonium salt **14** (Path B), is shown in Scheme 1.

The Wittig phosphorus ylides are typically obtained in situ from the corresponding phosphonium salts after treatment with a strong base, such as an organolithium compound (*n*-BuLi, LDA, LiHMDS), alkoxide (NaOEt or *t*-BuOK), metal hydride (NaH), or with sodium hydroxide or carbonate under phase transfer catalysis conditions. Preliminary experiments showed poor *E*/*Z*-stereoselectivity and the need for chromatographic separation of the products, regardless of the base type. We used the Boden modification [34], using K_2_CO_3_ as the base and 18-crown-6 as the phase transfer catalyst, because of the relatively low toxicity of the reagents and no requirement for an inert atmosphere and scrupulously dried solvents. The reaction was conducted in a mixture of THF/dichloromethane (2/1) under reflux and yielded 57–87% of an approximately equimolar mixture of the *E*/*Z*-stilbenes (Table 1).

**Scheme 2:**
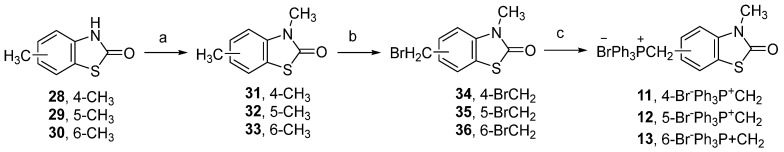
Synthesis of heterocyclic phosphonium salts **11**–**13**, containing a benzothiazolone moiety. Reagents and conditions: (**a**) (CH_3_)_2_SO_4_, aq. NaOH, r.t., 1 h, 80–89%; (**b**) *N*-Bromosuccinimide (NBS), (PhCO)_2_O_2_, CCl_4_, reflux, 3 h, 63–84%; (**c**) PPh_3_, chlorobenzene, reflux, 85–96%.

Methoxy substituted benzaldehydes **5**–**8** are commercially available and inexpensive. As previously described by us [35], 3-methyl-2(3*H*)-benzothiazolone-6-carbaldehyde (**9**) was obtained by Duff reaction. The main challenge was the synthesis of the phosphonium salts **11**–**13**, obtained in three steps from the corresponding 4-, 5-, or 6-methyl-2(3*H*)-benzothiazolones **28**–**30** (Scheme 2).

**Scheme 3:**
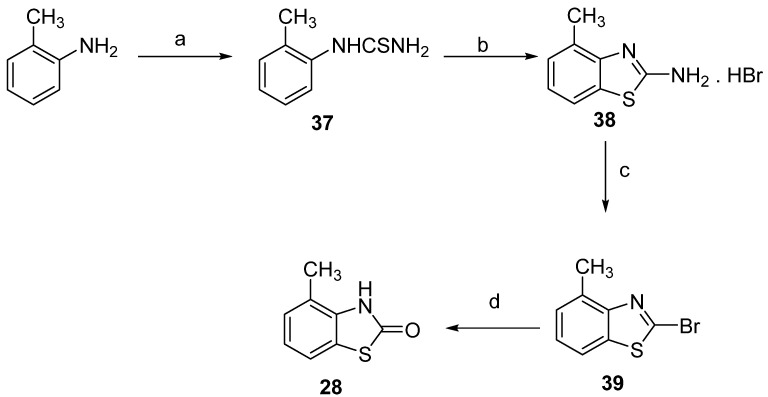
Synthesis of 4-methyl-2(3*H*)-benzothiazolone (**28**). Reagents and conditions: (**a**) NH_4_SCN, HCl, reflux, 77%; (**b**) Br_2_, CHCl_3_, 60 °C, 81%; (**c**) NaNO_2_, HBr, ClCH_2_CH_2_Cl, 5 to 40 °C, 89%; (**d**) 37% HCl, 2-methoxyethanol, reflux, 85%.

The methyl-2(3*H*)-benzothiazolones **28**–**30** were obtained by three different methods, depending on the position of the methyl substituent. The first, 4-Methyl-2(3*H*)-benzothiazolone (**28**), was obtained by acid hydrolysis of 2-bromo-4-methylbenzothiazole (**39**) (the synthesis procedure is described in detail in Supplementary Materials Section S.1.2) [36]. The synthesis of the latter is shown in Scheme 3 and has been described previously [37].

**Scheme 4:**
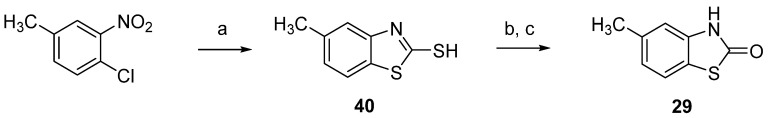
Synthesis of 5-methyl-2(3*H*)-benzothiazolone (**29**). Reagents and conditions: (**a**) Na_2_S_x_, CS_2_, water, reflux, 47%; (**b**) aq. KOH, KMnO_4_; (**c**) 37% HCl, water, reflux, 88%.

As anticipated, efforts to generate 5-methyl-2(3*H*)-benzothiazolone (**29**) by the analogous synthetic route (Scheme 3) led to a mixture of the 5-methyl- and 7-methyl-2(3*H*)-benzothiazolone regioisomers which were difficult to separate [38]. Seeking an alternative regioselective method for obtaining 5-methyl-2(3*H*)-benzothiazolone (**29**), we focused on the oxidation of 2-mercaptobenzothiazoles (benzothiazole-2-thiols) to benzothiazole-2-sulfonic acids and subsequent acid hydrolysis. Using a method described by Dunbook and Zimmerman [39,40], 2-Mercapto-5-methylbenzothiazole (**40**) was obtained from 4-chloro-3-nitrotoluene. Subsequent oxidation of the thiol **40** and its hydrolysis to **29** was carried out as proposed by Efros and Dawidenkow [41,42] (Scheme 4 and Supplementary Materials Sections S.1.3.1 and S.1.3.2).

**Scheme 5:**
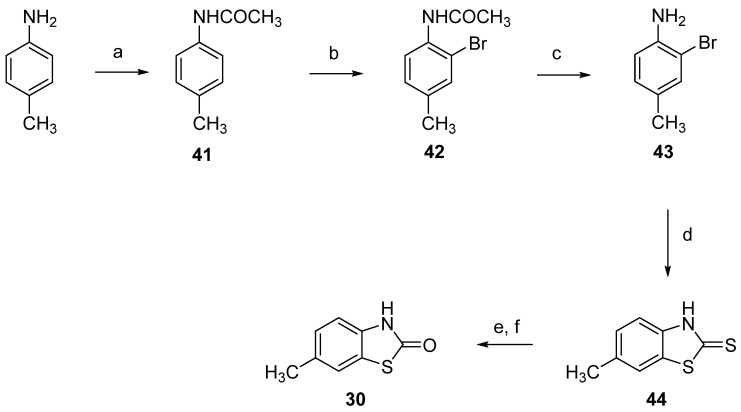
Synthesis of 6-methyl-2(3*H*)-benzothiazolone (**30**). Reagents and conditions: (**a**) (CH_3_CO)_2_O, toluene, 87%; (**b**) Br_2_, AcOH, 76%; (**c**) 37% HCl, EtOH, 67%; (**d**) KSC(S)OC_2_H_5_, *N*-Methyl-2-Pyrrolidone (NMP), 160 °C, 81%; (**e**) aq. KOH, KMnO_4_; (**f**) 37% HCl, water, reflux, 91%.

Similarly to **29** (Scheme 5), 6-Methyl-2(3*H*)-benzothiazolone (**30**) was synthesized from the corresponding 6-methyl-2-mercaptobenzothiazole ((**44**), Supplementary Materials Sections S.1.4.1, S.1.4.2. and S.1.4.3), but the latter was built by cyclization of the 2-bromo-4-methylaniline (**43**) obtained from *p*-toluidine [43] by heating in the presence of potassium ethyl xanthogenate in *N*-methyl-2-pyrrolidone (NMP) [44,45].

**Scheme 6:**
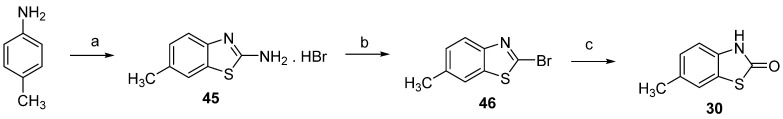
Synthesis of 6-methyl-2(3*H*)-benzothiazolone (**30**). Reagents and conditions: (**a**) NH_4_SCN, Br_2_, AcOH, 80%; (**b**) NaNO_2_, HBr, ClCH_2_CH_2_Cl, 0 °C to r.t, 75%; (**c**) 37% HCl, 2-methoxyethanol, reflux, 89%.

Despite excellent yields, the reaction between 2-bromo-4-methylaniline (**43**) and potassium ethyl xanthogenate was associated with the emission of a noxious odor, which prompted us to test alternative methods. We found that 6-methyl-2(3*H*)-benzothiazolone (**30**) could be synthesized analogously to 4-methyl-2(3*H*)-benzothiazolone (**28**) from 2-bromo-6-methylbenzothiazole (**46**) [46,47,48], as shown in Scheme 6.

**Scheme 7:**
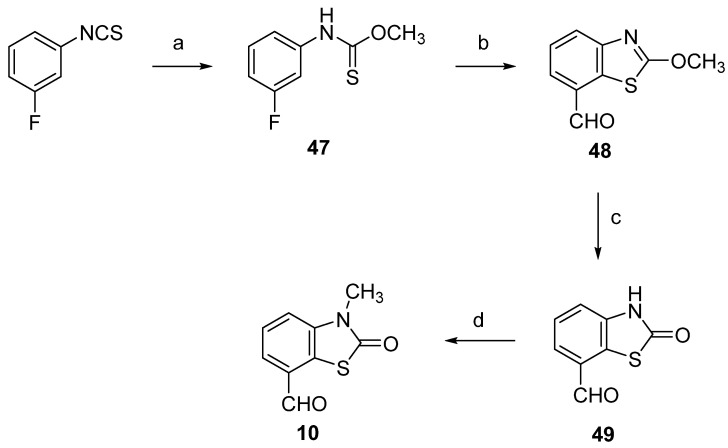
Synthesis of 3-methyl-2(3*H*)-benzothiazolone-7-carbaldehyde (**10**). Reagents and conditions: (**a**) MeOH, Et_3_N, reflux, 79%; (**b**) 1 eq. *n*-BuLi, −60 °C, then 1.6 eq. *tert*-BuLi, −80 to −40 °C, DMF −80 to −20 °C, 60%; (**c**) 37% HCl, EtOH, reflux, 83%; (**d**) CH_3_I, K_2_CO_3_, DMF, 35 °C, 84%.

As commented on above, obtaining pure 7-methyl-2(3*H*)-benzothiazolone free of regioisomers is difficult. Therefore, we chose to obtain (*Z*)-3-methyl-7-(3,4,5-trimethoxystyryl)-2(3*H*)-benzothiazolone **27E/Z** by path B, the Wittig reaction of 3-methyl-2(3*H*)-benzothiazolone-7-carbaldehyde (**10**) and (3,4,5-trimethoxylbenzyl)triphenylphosphonium bromide **14** (Scheme 7). For comparison of the stereoselectivity of path A and B, we also synthesized the compound by path B. As expected, the two Wittig reactions yielded similar *E*/*Z* stereoselectivity (Table 1). The synthesis of **10** was conducted following Stanetty and Krumpak [49], with minor modifications (Scheme 7). The *O*-methyl (3-fluorophenyl)carbamothioate (**47**) was converted to the lithium thiolate with one equivalent of *n*-BuLi, followed by *ortho* metalation with *tert*-BuLi at −80 °C in THF. The addition of DMF led to the isolation of 2-methoxybenzothiazole-7-carbaldehyde (**48**). Acid hydrolysis and subsequent *N*-methylation of 2(3*H*)-benzothiazolone-7-carboxaldehyde (**49**) led to the target aldehyde **10**.

The *E*- vs. *Z*-configuration of stilbene derivatives is typically determined by ^1^H NMR spectroscopy, based on the *J*-coupling constant of the olefin proton resonances. As a general rule, the *Z*-isomers display two doublets in the 6.33–6.75 ppm range with a *J*-constant around 12 Hz, while the *E*-isomer olefinic protons appear around 6.94–7.46 ppm as doublets, with a *J*-constant of 16 Hz. Uncharacteristically, the olefinic protons of the 3,5-dimethoxyphenyl (**25Z**) and 3,4,5-trimethoxyphenyl (**26Z/E**) stilbenes overlapped in CDCl_3_, resulting in singlet resonances. However, the signals were resolved in acetone-d_6_, which allowed the determination of the products’ stereochemistry (*J* = 12.2 for the *Z* isomer and 16.3 Hz for the *E* isomer, respectively). The detailed information of NMR spectra is shown in Supplementary Materials (Figures S4.1.1–S4.14.2).

### 2.2. X-ray Crystallography and Docking

#### 2.2.1. Single Crystal X-ray Diffraction

**Figure 2:**
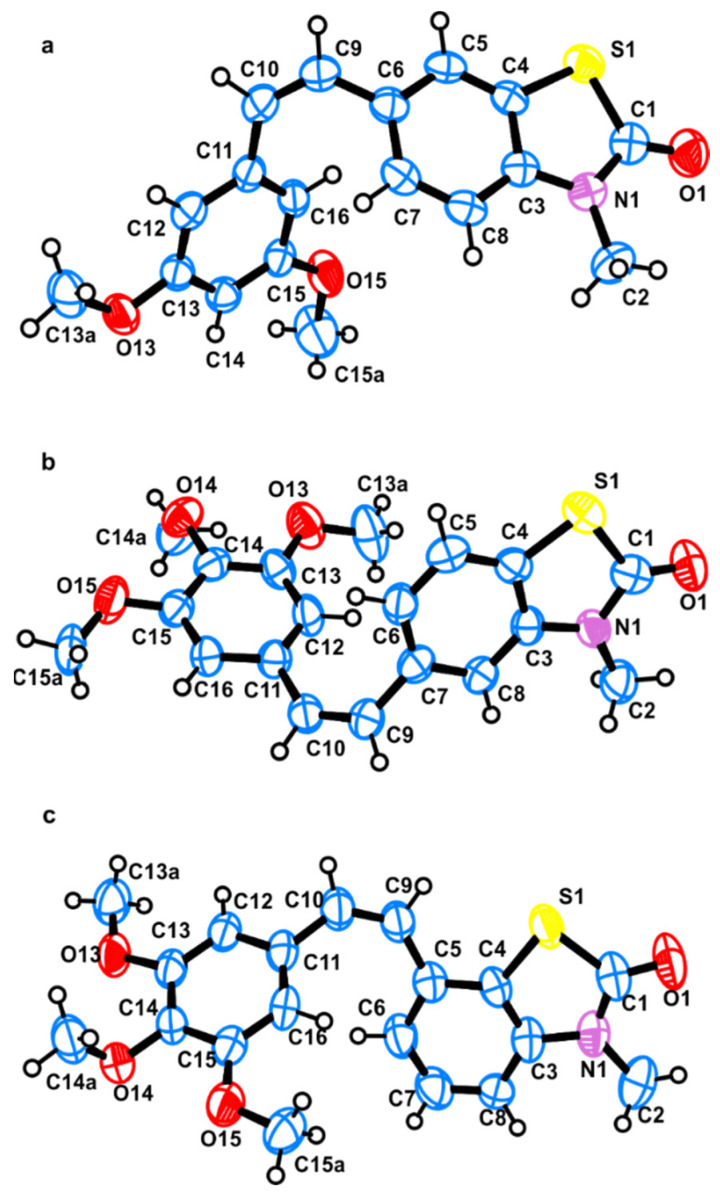
ORTEP view with the atom-numbering scheme of the molecules present in the asymmetric unit of (**a**) **25Z**, (**b**) **22Z**, and (**c**) **27Z**; atomic displacement parameters are at the 50% probability level.

Single-crystal X-ray structure analysis was performed to precisely assign the *E*/*Z* conformation of the newly synthesized compounds. From the 26 new benzothiazolones, single crystals with suitable quality for data collection were grown by slow evaporation for compounds **19Z**, **22Z**, **22E**, **23Z**, **24Z**, **25Z**, **26E,** and **27Z**. ORTEP [50] views of the molecules present in the asymmetric unit (ASU) of the biologically active compounds (e.g., **25Z**, **22Z,** and **27Z**) are shown in Figure 2 and Figure S2.1–S2.5. The most important crystallographic and data refinement parameters from the X-ray diffraction experiment are given in Table S2.1. Compounds **25Z**, **22Z,** and **27Z** crystallize as *Z*-configurational isomers in the corresponding orthorhombic Pca2_1_, Pcab, and monoclinic C2/c space groups. Further, **27Z**, **25Z,** and **22Z** are also positional isomers based on the styryl–benzothiazole link (C9–C5/C6 or C7), even though **25Z** has only two methoxy groups, whereas **27Z** and **22Z** have three.

**Figure 3:**
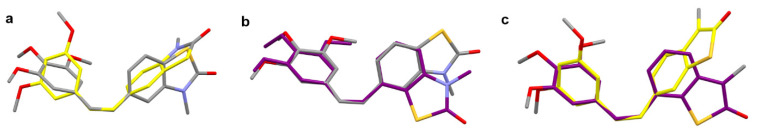
Overlay based on the common for all compounds (–C–CH=CH–C–) bridge of (**a**) **22Z** (grey) and **25Z** (yellow), (**b**) **22Z** (grey) and **27Z** (purple), and (**c**) **25Z** (yellow) and **27Z** (purple), disclosing the structural flexibility of the molecules.

The bond lengths, angles, and torsion angles of the molecules of **19Z**, **22Z**, **22E**, **23Z**, **24Z**, **25Z**, **26E,** and **27Z**, determined from the single crystal X-ray experiments, are comparable with other structures in the Cambridge structural database [ref. codes: TERSUF, WIJSEN, AFOPOC [51,52,53] (Table S2.2). The benzothiazolone and methoxybenzene (mono-, di-, or tri-substituted) moieties are almost planar, with rmsd values < 0.03 Å. However, if we compare their relative orientation regarding the plane involving the double bond (–C–CH=CH–C–), there is a significant deviation of the angles between the mean planes of the benzothiazolone and methoxybenzene rings. The twist and fold angles between the rings and the (–C–CH=CH–C–) plane and those between the normal of the planes of the ring systems vary considerably (Table S2.4). Those values suggest considerable structural (conformational) flexibility (Figure 3) of the synthesized molecules that would enhance ligand–protein interaction. Compounds **25Z**, **22Z,** and **27Z** have non-typical donors (CH_3_), while the acceptors may be C-O_methoxy_ and/or C=O. Those molecular features result in the formation of weak intramolecular and intermolecular weak/hydrogen bonding interactions of C-H_aromatic_⋯C=O, C-H_aromatic_⋯C-O_methoxy_, C-H_methyl_⋯C=O, and C-H_methyl_⋯C-O_methoxy_ type (Table S2.3), that stabilize the molecular and crystal structures. The three-dimensional packing of the molecules in the crystal structures of **25Z** and **22Z** shows zig-zag rearrangement along with *c*– and *b*–cell axes, respectively.

#### 2.2.2. Molecular Docking of **26Z**, **25Z**, **22Z**, and **27Z** in the Colchicine Site of α,β-Tubulin

**Figure 4:**
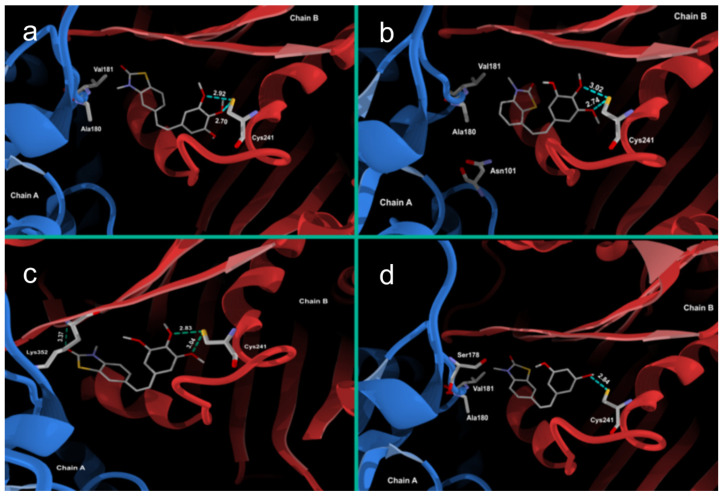
Molecular docking of (**a**) **22Z**, (**b**) **27Z**, (**c**) **26Z** and (**d**) **25Z** in the colchicine site of α,β-tubulin, along with observed interactions.

The ligand-protein interactions between the molecular models of compounds **26Z**, **25Z**, **22Z**, and **27Z** and the tubulin-colchicine:stathmin-like domain complex (PDB code: 1SA0 [54]) were investigated. The structural similarity of synthesized compounds with colchicine suggested docking in the colchicine site of the α,β-tubulin complex. The colchicine binding site lies on the border between the α and β subunits of tubulin; therefore, docking there could lead to inhibition of microtubule polymerization. Colchicine’s mechanism of action is based on hydrogen bonding interactions between methoxy groups and Cys241 and Val181 amino acids [19]. However, for compounds **25Z**, **22Z**, and **27Z**, only interactions with Cys241 are detected (Figure 4).

Crystals of compound **26Z** suitable for a single crystal X-ray experiments were not obtained. The molecular model of **26Z** was software generated [55] based on the crystal structure of **25Z** and the benzoxazolone analog **4** (OP-107) [19], where the difference is an O atom instead of an S atom in the benzoxazolone/benzothiazole moiety. The generated molecular structure of **26Z**, docked in the colchicine site, displayed interactions with Cys241 and Lys352, while the docking results for compounds **22Z**, **27Z,** and **25Z** only showed an interaction with Cys241.

### 2.3. Biology Experiments

#### 2.3.1. Evaluation of Benzothiazolone CA-4 Analog’s Cytotoxic Activity in Human Endothelial and Tumor Cell Lines

The cytotoxic effect of benzothiazolone cis/trans-CA-4 analogs was evaluated using the MTT assay in the endothelial EA.hy926 cell line, since it was shown that the antitumor activity of combretastatin is mainly dependent on its anti-vascular activity [56]. After 72 h of treatment, *Z*-benzothiazolone analogs inhibited cell growth and showed cytotoxic activity in a concentration-dependent manner. *E*-analogs did not show significant activity at concentrations up to 250 μM, except for **26E**. The results are shown in Table 2.

All 13 cis-compounds showed growth inhibitory activity against EA.hy926 cells, with IC_50_ values between 0.13 and 26 µM. **26Z** exhibited the most potent cytotoxic activity with an IC_50_ of 0.13 ± 0.01 μM, which was three to hundreds of times lower than the other analogs. The **26E** trans-form of **26Z** was also active at higher concentrations against endothelial cells with IC_50_ = 38.20 ± 5.35 μM. It is well known that the affinity of *E*-isomer of CA-4 to α,β-tubulin is significantly less potent [57,58]. In general, cis-isomers gave much stronger antiproliferative activities over their trans-isomers, and our data confirmed it, since only the *E*-isomer of the most active compound (**26Z**) showed some activity.

The second most active compound was **27Z**, with IC_50_ = 0.30 ± 0.05 µM. The third most potent compound was **25Z**, with an IC_50_ = 1.88 ± 0.18 µM. The cytotoxic activity of the tested *Z*-benzothiazolones can be ranked as follows: **26Z** > **27Z** > **25Z** > **24Z** ≅ **22Z** ≅ **21Z** > **19Z** > **20Z** ≅ **18Z** > **17Z** ≅ **Z23** > **15Z** ≅ **16Z**. The **26Z** positional isomers **27Z**, **22Z,** and **18Z** demonstrated loss of activity, suggesting that the position of the styryl fragment in ring **B** plays an essential role in the analogs’ bioactivity. Amongst the most active compounds, three (**22Z**, **26Z**, **27Z**) out of four have three methoxy groups in ring **A**. The combination of a styryl fragment at position **6** in ring **B** with three OCH_3_ groups in ring **A** showed the most prominent cytotoxic effect on EA.hy926 cells. The position of the styryl fragment in ring **B** relative to the compounds’ activity can be ranked as **6** > **7** > **5** > **4** and is critical for the observed cytotoxic activity.

The docking analysis also confirmed the importance of methoxy groups in ring **A** together with the position of the styryl fragment in ring **B**. Compound **26Z** shows a hydrogen bonding interaction with *Cys241* and an additional interaction with *Lys352*, which could explain the greater cytotoxic effect of **26Z** against EA.hy926 cells.

The most effective compound, **26Z**, was further tested for cytotoxicity on CA-4 resistant tumor cells. A549, MDA-MB-231, and HT-29 cells are known to be relatively resistant to anticancer agents and CA-4 [59,60,61]. When we treated HT-29 with CA-4, the IC_50_ was 2.16 ± 0.23 µM (Table S3.2, Figure S3.1), while **26Z** showed IC_50_ = 0.008 ± 0.001 µM. Compared to CA-4, **26Z** exhibited a more than 250 times greater cytotoxic effect on colorectal adenocarcinoma HT-29 cells. In the range of 10 nM to 25 µM, **26Z** showed cytostatic activity with 40% viable Colon-26 cells after 72 h, compared to CA-4 with IC_50_ = 660 ± 82 nM.

In A549 cancer cells, a cytotoxic effect with IC_50_ = 3.01 ± 0.16 µM was observed for CA-4, while **26Z** showed cytostatic activity with 45% cell viability at concentrations from 10 nM to 25 µM (Table S3.2). In control BEAS-2B cells, the cytostatic activity was around 80% (viable cells) for both CA-4 and **26Z**. The greater cytotoxic activity of **26Z** against A549 cells compared to CA-4 and the low toxicity in BEAS-2B control cells suggest a possible functional advantage of **26Z** against lung cancer cells.

In MCF-7 breast cancer cells, we observed a cytotoxic effect with IC_50_ = 2.42 ± 0.48 µM for **26Z**, where CA-4 showed a cytostatic impact (40% viable cells) in the concentration range studied. Compound **26Z** has IC_50_ = 1.35 ± 0.42 µM in MDA-MB-231 and a cytostatic activity with 65% viability of the control MCF-10A cell line. It is essential to emphasize the cytotoxic effect of **26Z** at concentrations over 1 µM in triple-negative breast cancer MDA-MB-231 cell lines, while the control cells have 65% viability. Triple-negative breast cancer accounts for about 15% to 20% of all breast cancer cases, and it is difficult to treat. Further experiments are needed to clarify the molecular differences observed between the effect of **26Z** on MDA-MB-231 and MCF-10A cells at concentrations over 1 µM, indicating a potent anti-proliferative effect and suggesting a possible functional advantage of **26Z** when compared to CA-4.

Based on the observed cytotoxic effects, we have selected the three most active compounds (**26Z**, **27Z**, and **25Z**) to conduct further studies and gain insights into the cellular and molecular mechanisms of **26Z**’s action on EA.hy926 endothelial cells.

#### 2.3.2. **25Z**, **26Z**, and **27Z** Decrease the Clonogenic Survival of Endothelial Cells

The clonogenic assay has become the “gold standard” for assessing cellular sensitivity to cytotoxic treatments, as the ability to proliferate and form a colony is a fundamental aspect of cell survival. Cell survival assays measure the end result of treatment, which can be either cell death or survival [62]. To determine whether exposure to increasing **25Z, 26Z**, and **27Z** concentrations (0.01, 0.25, 2.5, 10, and 25 μM) affects the proliferation ability and colony-forming capacity of EA.hy926 cells, we evaluated the late lethality, following treatment for 72 h and 6 days of recovery.

**Figure 5:**
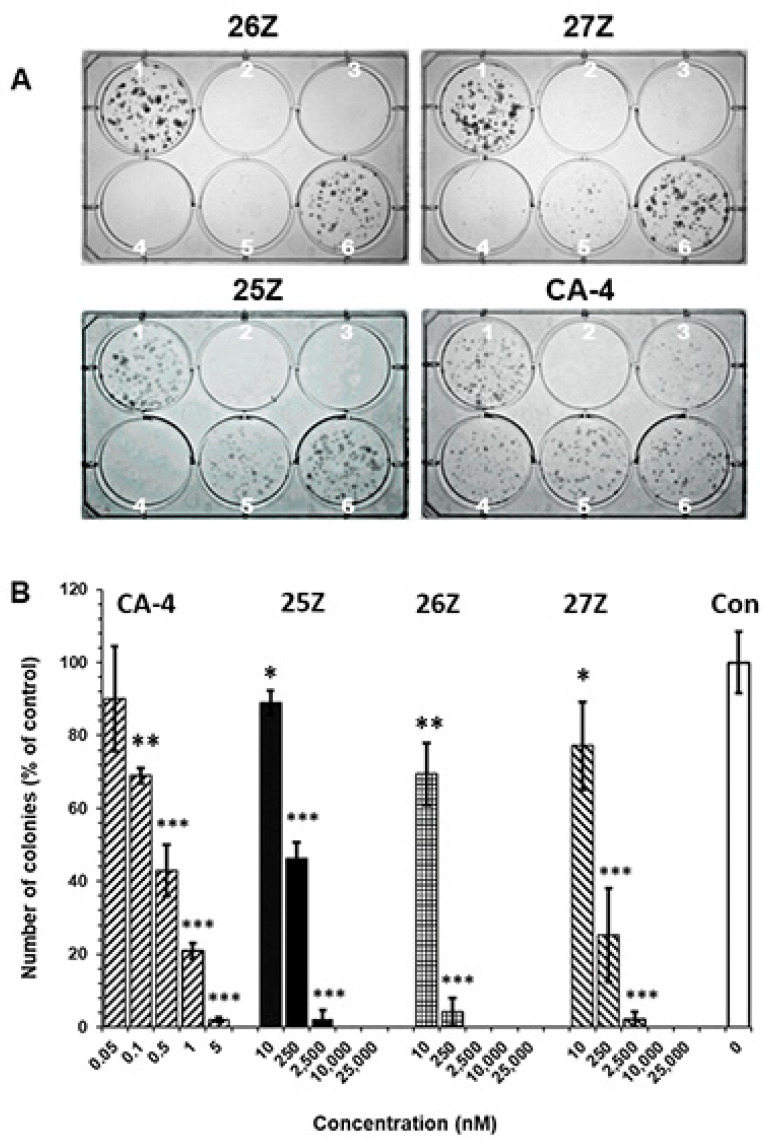
Effect of **25Z**, **26Z**, **27Z**, and CA-4 on colony forming capacity (clonogenic survival) of exponentially growing EA.hy926 cells after treatment for 72 h. (**A**) EA.hy926 cells were treated with: (1) 0.01 μM DMSO, (2) 25 μM, (3) 10 μM, (4) 2.5 μM, (5) 0.25 μM, and (6) 0.01 μM concentrations of **26Z**, **27Z**, **25Z**, and: (1) 0.01 μM DMSO, (2) 1 nM, (3) 0.5 nM, (4) 0.1 nM, (5) 0.05 nM, and (6) 0.01 nM of CA-4 as a positive control. Cells were allowed to form colonies in fresh medium for 6 days after treatment. (**B**) Number of colonies formed on day 6 after treatment normalized to control. The presented data were obtained by manual counting and expressed as mean ± SD (*n* = 3). * *p* < 0.05; ** *p* < 0.01; ***p < 0.001.

The results indicate that **25Z**, **26Z**, and **27Z**, as well as CA-4, significantly inhibit the proliferation and colony formation capabilities of EA.hy926 cells and show persistent effects at concentrations well below the IC_50_, as determined by MTT assay (Figure 5). Semiquantitative analysis showed a strong reduction (about 100%) in colony formation following treatment with 2.5, 10, and 25 µM concentrations of **25Z**, **26Z**, and **27Z**. EA.hy926 cells were more sensitive towards **26Z**, **27Z**, and **25Z** exposure at the lowest dose of 10 nM. Moreover, a 90% decrease in colony number was observed at 250 nM **26Z**, with decreases of 75% and 55% for **27Z** and **25Z**, respectively (Figure 5B). The effect of CA-4 was more pronounced compared to **26Z**, **27Z**, and **25Z**. CA-4 at 1 nM inhibited endothelial cell colony formation by 80%.

The results also showed that many cells treated with CA-4 analogs at concentrations lower than the IC_50_, as determined by the MTT assay, could not continue dividing and forming colonies when the compounds were removed and the cells were allowed to grow in a fresh medium for six days. We found that EA.hy926 cells were more sensitive to CA-4 and **26Z** compared to **25Z** and **27Z**.

To investigate the precise mechanism responsible for the **26Z**-mediated anti-proliferative effects, we examined the cell-cycle distribution profile of **26Z**-treated EA.hy926 cells over time.

#### 2.3.3. **26Z** Causes Mitotic Arrest in Treated EA.hy926 Cells

**Figure 6:**
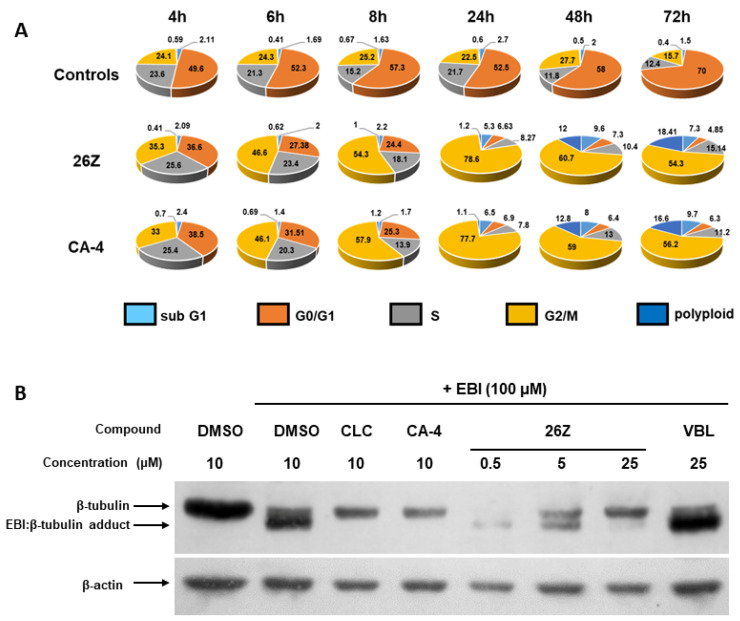
Effect of **26Z** and CA-4 on the cell cycle (**A**) and EBI competition assay in EA.hy926 cells (**B**). DNA content was measured in asynchronously growing cells using propidium iodide staining at 4, 6, 8, 24, 48, and 72 h after treatment with 10 nM CA-4 or 300 nM **26Z**. The results are shown as the percentage of the cells in different cell cycle phases. Cytograms are representative of three independent experiments. In (**B**), EA.hy926 cells were incubated with or without indicated concentrations of the compounds for 2 h, then 100 μM EBI was added to cells and incubated for another 2 h. Total proteins were lysed and subjected to western blot analysis for β-tubulin and β-actin. VBL: vinblastine; CLC: colchicine; EBI: *N*,*N*′-ethylenebis(iodoacetamide).

In EA.hy926 cells, **26Z** treatment caused a significant inhibition of cell-cycle progression, resulting in an accumulation of treated cells in the G2/M phase compared to control cells (Figure 6, Figure S3.2). This effect is similar to the one observed for CA-4. The G2/M population began to rise as early as 4 h post-treatment (~35%) and reached its maximum at around 24 h (~78%), and was still above 50% at 48 h and 72 h. At 48 h and 72 h, G2/M arrested cells decreased, mainly because of increased apoptotic hypodiploid DNA content peak (sub-G1) and polyploid cells. The observed cell cycle distribution shows that cell death is a direct consequence of mitotic arrest, although some cells can overpass it (polyploid > 4N cells).

Tubulin-binding agents cause a cell cycle blockade due to disturbances in microtubule dynamics. Previous studies on CA-4 activity have confirmed that it causes G2/M phase mitotic arrest in treated cells, as the cells are unable to complete the process of cell division [63,64,65].

Due to the structural similarity between our compounds and CA-4, which target the colchicine-binding site of tubulin, further studies were performed to investigate the specificity of **26Z** interaction at the colchicine binding site of tubulin using *N*,*N*-ethylenebis(iodoacetamide) (EBI) in EA.hy926 cells. EBI forms adducts with tubulin which can be detected by Western blotting. Microtubule destabilising agents that bind at the colchicine site, such as colchicine and CA-4, prevent the formation of the β-tubulin-EBI adduct by occupying the binding site [66,67]. EA.hy926 cells were treated with vehicle control (10 μM DMSO), 10 μM colchicine, 10 μM CA-4, 25 μM vinblastine, or different concentrations of **26Z** for 2 h, followed by EBI (100 µM) for an additional 2 h. Control samples show the presence of the β-tubulin-EBI adduct at a lower position, indicating that EBI has crosslinked Cys239 and Cys354 amino acids on the β-tubulin (Figure 6B). In contrast, tubulin EBI-adduct formation was inhibited in EA.hy926 cells treated with 10 μM CA-4, 10 μM colchicine, and 25 µM **26Z**. Figure 6B also shows that binding of **26Z** to the tubulin is concentration dependent, since with increasing the **26Z** concentration, the EBI-adduct formation was decreased. These data proved that **26Z** binds specifically to the colchicine site of β-tubulin.

Cancer cell migration and motility are critical factors in tumor progression and metastasis, and the endothelial cells recruited to the tumor undergo rapid cell migration, proliferation, and differentiation. Endothelial cell migration is of crucial importance for angiogenesis. Based on the results described above, we hypothesized that EA.hy926 cells will have impaired cell motility. A wound-healing assay was performed to investigate the effect of compounds **25Z**, **26Z**, and **27Z** on EA.hy926 cell migration.

#### 2.3.4. In Vitro Cell Migration Assay

**Figure 7:**
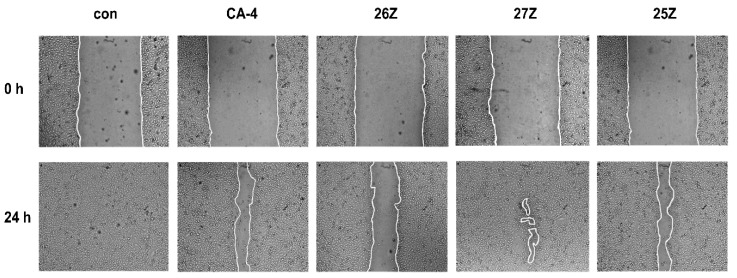
Inhibition of cell migration of EA.hy926 cells treated for 24 h with CA-4, **25Z**, **26Z**, and **27Z** in the wound healing assay. Representative images of 3 independent assays. **25Z**, **26Z**, and **27Z**: 50 nM, CA-4: 1 nM, Con: 0.01 µM DMSO.

As illustrated in Figure 7, the control cells (0.01 µM DMSO) migrated to the scraped area while **25Z**, **26Z**, and **27Z** significantly inhibited cell migration at sub-toxic doses of 50 nM, and 1 nM for CA-4.

These doses were chosen to avoid the effect of toxicity on cell migration. The remaining mean wound opened areas in the treated cell monolayers are as follows: CA-4 = 24.4% ± 4, **25Z** = 22.4% ± 8, **26Z** = 49% ± 6, and **27Z** = 3% ± 1. The leading compound **26Z** is the most anti-mitogenic after 24 h of treatment (Figure 7). At low concentrations, **26Z** can significantly impair (*p* < 0.001) endothelial cell migration. This significant difference in repairing the wounded area in the cell monolayers confirms that **25Z** and **26Z** suppressed endothelial cell migration.

Beyond the migrating front, endothelial cells usually proliferate to generate the necessary number of cells for making new blood vessels. Combretastatins are vascular disrupting agents, and their ability to selectively disrupt already established tumor blood vessels is critical for their in vivo antitumor activity. To further investigate the effects of **25Z**, **26Z**, and **27Z** on neovascularization, we evaluated the effect of CA-4 analogs on tubular morphogenesis.

#### 2.3.5. In Vitro Anti-Vascular Activity of Benzothiazolone CA-4 Analogs

**Figure 8:**
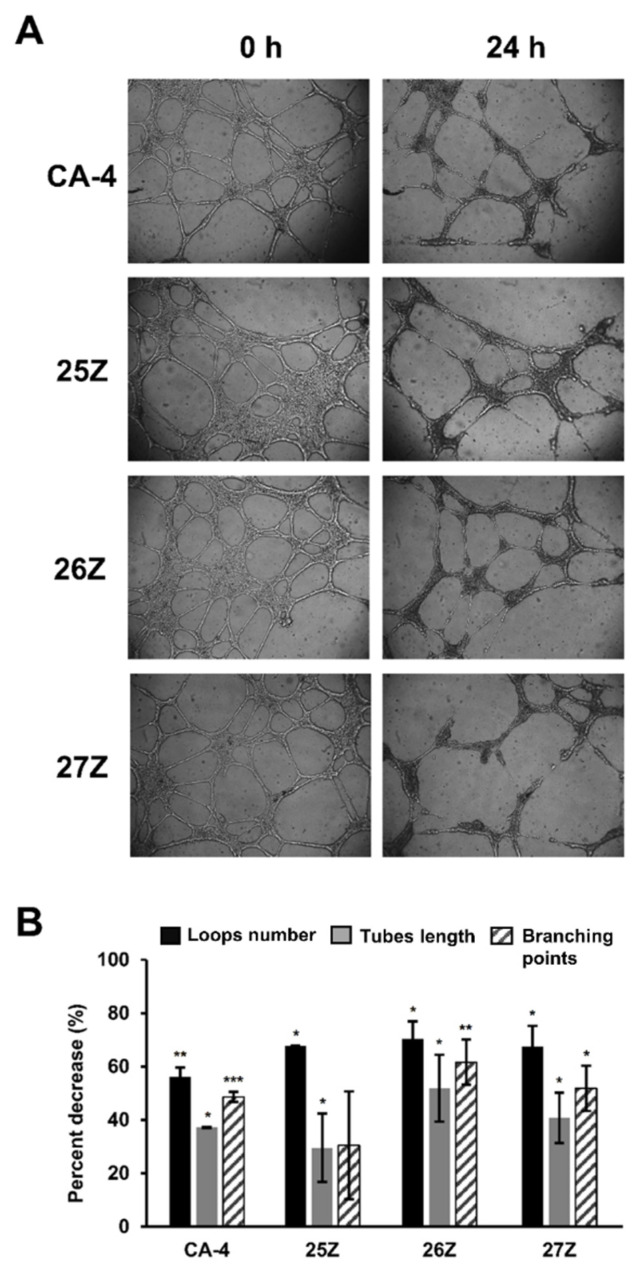
Tube-formation assay of EA.hy926 cells cultured on extracellular matrix (Matrigel). (**A**) Images of formed tubular networks taken after 18 h of incubation and subsequent destruction following 24 h of treatment with equitoxic IC_50_ concentrations of CA-4, **25Z**, **26Z**, and **27Z**. (**B**) Reorganizational analysis of structural network parameters—loop number, tubes length, and branching points. The results are shown as the average ± SD (*n* = 3). * *p* < 0.05; ** *p* < 0.01 *** *p* < 0.001 compared to 0 h.

Upon incubation for 24 h on the extracellular matrix model surface, EA.hy926 cells fuse into continuous tubules with a complete lumen to form capillary-like networks (Figure 8, time 0). After 24 h of treatment, IC_50_ concentrations of **26Z** and **27Z** destroy formed tubes with a significant reduction of ~70% in cumulative tube length, compared with length at time 0 (Figure 8A,B). Dimensional parameters such as tube length reflect cells’ ability to maintain an elongated form and are vital for in vitro vasculogenesis [68]. At the same time, topological parameters such as branch points and loop number contain important physiological information concerning the overall structural arrangement and complexity of the capillary-like tubular network [69]. Figure 8B shows the disruption of established tubes and branches, which is a good indication of a potential anti-vascular effect of the test compounds in vivo. All three analogs reduced loop numbers by about 65–70%, when CA-4-induced reduction was 56 ± 3% (*p* < 0.05). Also, **26Z** decreased the branching points (63%) to a greater extent than CA-4 (50%) (Figure 8B).

The results allowed us to conclude that the CA-4 analogs **25Z**, **26Z**, and **27Z** affect endothelial capillary tubule morphogenesis in vitro. Taken together, all results shown above indicated that **26Z** and **27Z** directly affected EA.hy926 cells by robustly inhibiting invasion, migration, proliferation, and capillary-tube formation, which are essential attributes of potential anti-angiogenic drug candidates [70].

The next step of our study was to identify the cellular basis of the proliferation, migration, and invasion defects observed upon CA-4 analog treatment.

#### 2.3.6. Aberrant Formation of Mitotic Spindles and Microtubule Network Alterations

**Figure 9:**
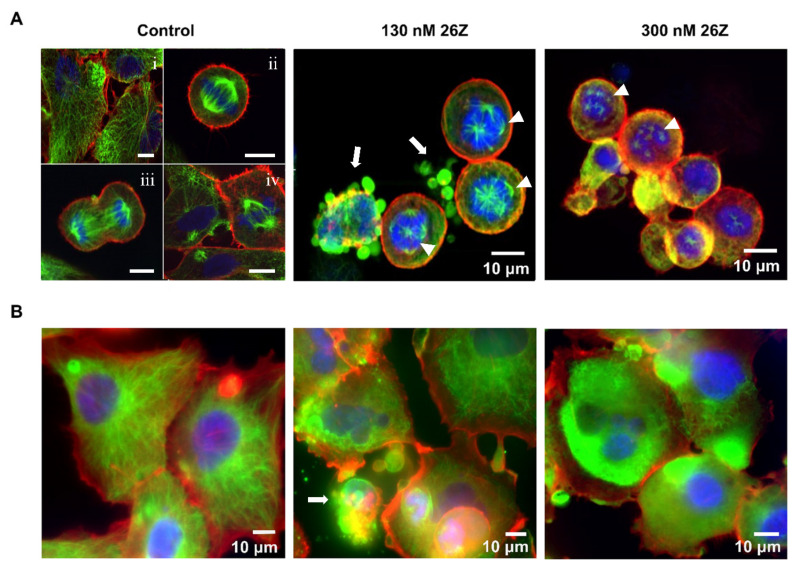
Indirect immunofluorescence staining of α-tubulin (green) and F-actin (red), and simultaneous DAPI chromosome staining (blue) of EA.hy926 cells. (**A**) Mitosis in control (i: interphase; ii: metaphase; iii: anaphase; iv: metaphase and cytokinesis) and **26Z** treated cells with IC_50_ (130 nM) and IC_80_ (300 nM) concentrations for 24 h. Microphotographs were obtained with an Andor Dragonfly 505 Confocal Microscope. (**B**) Cytoskeleton structure of adherent cells in control conditions and after treatment with **26Z** at IC_50_ (130 nM) and IC_80_ (300 nM) for 24 h. Images were acquired with an Axiovert 200 M inverted microscope. Scale bars, 10 μM. Arrowheads—multipolar spindles, arrows—membrane blebbing and apoptotic bodies.

Figure 9 shows the immunofluorescence micrographs of dividing EA.hy926 cells treated with vehicle (0.01 µM DMSO, Control) and **26Z**. Interphase cells (i) showed typical radial arrays of microtubules (Figure 9A, Control, green fluorescence). The mitotic population (~25%, Figure 6) of the total number of cells in a G2/M cell-cycle phase displayed the hallmark features of a typical mitotic process. Congression of chromosomes at the metaphase plate (ii) followed by anaphase onset (iii), telophase, and cytokinesis are evident in Figure 9 (panel A, Control).

Following 24 h of treatment with **26Z,** large proportions of the EA.hy926 cells showed mitotic abnormalities. Typical mitotic defects included a failure of a number of chromosomes to align correctly on the metaphase plate, and the absence of two bipolar spindles with the centromeres of individual chromosomes randomly attached to either of the spindle poles (Figure 9, panel A, **26Z**). Compound **26Z** triggered conspicuous spindle multipolarity with a striking declustering of supernumerary centrosomes and/or apoptosis (130 nM and 300 nM). Treatment with IC_50_ (130 nM) **26Z** for 24 h induced multipolarity, predominantly with three to five poles (3.9 ± 0.99), while IC_80_ (300 nM) increased the number of poles, ranging between six and twelve (8.8 ± 2.3).

Multinucleated interphase cells in treated cultures were also visible (Figure 9B). Some of these cells displayed typical radial arrays of microtubules at 130 and 300 nM **26Z** (Figure 9, panel B, **26Z**). Some mitotically arrested cells somehow slipped out of mitosis at 24 h and appeared as conspicuously large multinucleated cells. Most of these cells were polyploid (>4N) and had exited mitosis, probably by mitotic slippage, without proceeding through normal anaphase and cytokinesis. In adherent cells treated with **26Z** at IC_50_, there were remains of the microtubule network after 24 h, but it was more disorganized, lacking the “hair-like” structural organization typical of control cells (Figure 9B). While most control cells displayed a well-formed microtubule network, with microtubules going out from the center towards the cell’s periphery, cells treated with **26Z** looked more rounded. The microtubule structure was fragmented and loosely organized. There were cells with multi-lobed, segmented nuclei, and apoptotic cells with membrane blebbing and formation of membrane-bound apoptotic bodies (Figure 9, panel B, **26Z**).

#### 2.3.7. **26Z** Acts as Polymerization Inhibitor in Ex Vivo Conditions

**Figure 10:**
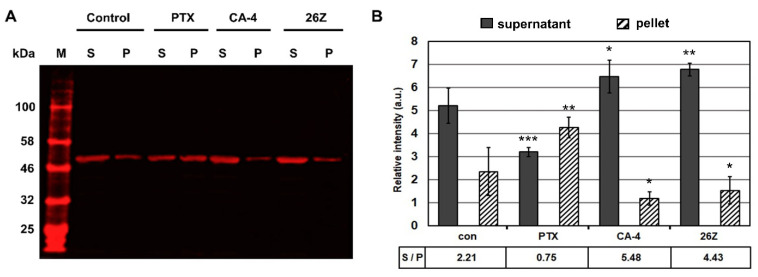
Ex vivo tubulin polymerization reaction at 37 °C in the presence of DMSO (control) or 20 μM of CA-4, paclitaxel, and **26Z**. (**A**) Representative image of western blots from the soluble (S) and polymerized (P) tubulin fractions. (**B**) Signal intensity analysis showing the stabilizing effect of paclitaxel (PTX) and the inhibitory effect of CA-4 and **26Z** on tubulin polymerization, illustrated by changes in the supernatant (S) to pellet (P) ratio. M—molecular marker. The results are shown as the average ± SD (*n* = 3). * *p* < 0.05; ** *p* < 0.01 *** *p* < 0.001 compared to control.

For a better and more specific characterization of the effect of **26Z** on tubulin polymerization, we performed an ex vivo polymerization experiment. Paclitaxel, CA-4, and **26Z** at a concentration of 20 μM were incubated with tubulin for 45 min at 37 °C, and the obtained microtubules were sedimented and analyzed, as described in the Methods section. The results showed that **26Z** acts as a tubulin polymerization inhibitor, although the effect is weaker than that of CA-4. Western blotting results showed that **26Z**, similarly to CA-4, inhibits the process of tubulin polymerization, demonstrated by an increase in the soluble fraction and a decrease in the polymeric fraction of tubulin (Figure 10A,B). On the contrary, paclitaxel, which was used as a positive control, caused a reduction in the soluble and an increase in the polymeric tubulin fraction.

**Figure 11:**
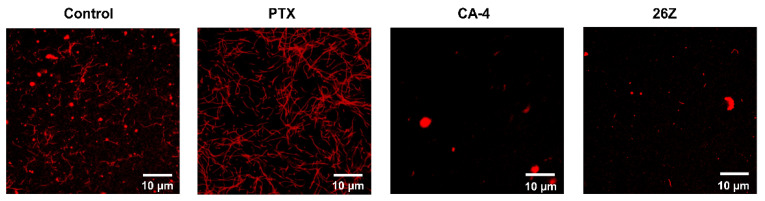
Fluorescence microscopy of ex vivo polymerized rhodamine-labeled α,β-tubulin. Representative images of formed microtubule fibers in control (DMSO 0.01 μM) and paclitaxel (PTX, 20 μM) samples, and inhibition of the process upon treatment with 20 μM CA-4 and **26Z**. Aggregates of non-polymerized tubulin are also visible as points with very bright fluorescence (Control, CA-4, and **26Z**). Scale bar, 10 μm.

These results confirm that **26Z** acts as an inhibitor of tubulin polymerization ex vivo. This was further corroborated by fluorescence microscopy analysis of rhodamine-labeled tubulin fiber formation in the presence of the tested compounds. Fluorescently labeled porcine tubulin together with unlabeled tubulin in a 1:3 ratio formed fluorescent fibers upon polymerization. The addition of CA-4 and **26Z** blocked the process of fiber formation compared to the control and paclitaxel (PTX) samples (Figure 11). The samples treated with **26Z** and CA-4 also contained visible non-polymerized tubulin aggregates. Consistent with previous results, paclitaxel addition promoted the formation of microtubule fibers without visible aggregates of non-polymerized tubulin.

#### 2.3.8. Benzothiazolones Depolymerizing Activity in EA.hy926 Cells

**Figure 12:**
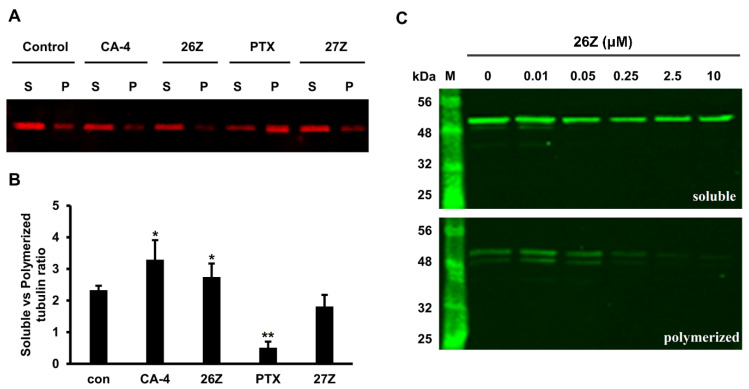
Western blot analysis of soluble and polymerized α,β-tubulin fractions after 6 h of treatment of EA.hy926 cells. (**A**) Western blot analysis of soluble (S) and polymerized (P) fractions isolated from EA.hy926 cells treated with 0.01 μM DMSO (control) or 1 μM of CA-4, **26Z, 27Z** and paclitaxel (PTX). (**B**) Graphic presentation of the soluble vs. polymerized tubulin ratio. (**C**) Concentration-dependent depolymerization of α,β-tubulin in EA.hy926 cells treated for 6 h with 0.01–10 μM **26Z**. All results are representative of three independent experiments. M: protein marker. * *p* < 0.05, ** *p* < 0.01 compared to control.

To confirm the previous observations that **26Z** can inhibit tubulin polymerization ex vivo, we performed an in vitro experiment following treatment of EA.hy926 cells with 0.01 µM DMSO and 1 µM of CA-4, paclitaxel, **26Z**, and **27Z** for 6 h. Extraction of the soluble (S) and polymeric (P) tubulin fractions from EA.hy926 cells was performed as described in the Materials and Methods (Section 3.3.8). Following electrophoretic separation (Figure 12A), the soluble vs. polymerized tubulin ratio was determined.

The results showed ratios of 2.33 ± 0.14 in DMSO control, 0.51 ± 0.19 in paclitaxel treated cells, 3.3 ± 0.61 for CA-4, 2.74 ± 0.43 for **26Z,** and 1.82 ± 0.36 for **27Z** treated cells. Compared to CA-4, **26Z** showed milder inhibitory activity after 6 h. The opposite effect was observed in cells treated with paclitaxel (Figure 12B). The depolymerizing activity of **26Z** is concentration-dependent, and the effect is clearly visible at concentrations higher than 0.05 μM (Figure 12C). Compared to the control DMSO-treated cells, **27Z** did not show a significant difference in α,β-tubulin polymerization. These results confirm the inhibitory action of **26Z** on α,β-tubulin polymerization in cells.

#### 2.3.9. **26Z** Activates Apoptotic Signaling Pathways in EA.hy926 Cells

**Figure 13:**
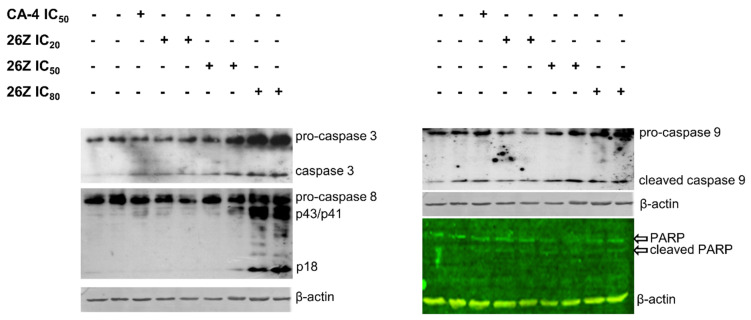
Western blot analysis of cell lysates from EA.hy926 cells treated with **26Z** and CA-4. Activation of receptor and effector caspases 8, 9, and 3 is shown in representative images following 24 h of treatment with **26Z** and CA-4.

The cell fate and pathways for initiation and execution of cell death can differ, ranging from apoptosis, necrosis, mitotic catastrophe, mitotic slippage, etc. Apoptosis, or programmed cell death, is a precisely regulated intracellular process leading to cell death without the induction of inflammation. Induction of apoptosis in proliferating endothelial cells and apoptotic changes in cell morphology are particularly important for disruption of tumor vasculature and blood flow shutdown. We observed the presence of apoptotic cells following treatment with **26Z** (Figure 6 (sub-G1) and Figure 9). To clarify the mechanism of cell death, we studied the activation of caspase signal-transduction pathways by immunoblot analysis of caspase 3, 8, and 9 protein levels. Active forms of effector caspase 3 were detected in cells treated with **26Z** at IC_50_ and IC_80_ (0.30 ± 0.04 μM) and, to a lesser extent, in samples treated with CA-4 IC_50_ and **26Z** IC_20_ (0.04 ± 0.01 μM, Figure 13). There was also observable proteolytic fragmentation of PARP, the downstream substrate of caspase 3, in all EA.hy926 treated cells. Our results showed that caspases 8 and 9 were also activated by **26Z** treatment. The activation of initiator caspase 8 was more pronounced in cells treated with **26Z** IC_80_, while caspase 9 was active in all endothelial cells treated with CA-4 and different concentrations of **26Z** (Figure 13).

Activating all critical components of the apoptotic cascade confirms that **26Z** can induce apoptosis in EA.hy926 cells through extrinsic and intrinsic pathways.

## 3. Materials and Methods

### 3.1. Chemistry

#### 3.1.1. General Information

Melting points (m.p.) were determined on a Boetius hot-stage microscope. ^1^H and ^13^C NMR spectra were obtained with a Bruker DRX250, Bruker DRX400, and DRX 500 spectrometer (Bruker BioSpin GmbH, Rheinstetten, Germany) in CDCl_3_ or acetone-d_6_ as solvent. Chemical shifts were reported in parts per million (ppm, *δ*) relative to the solvent peak (7.26 ppm for ^1^H; 77.16 ppm for ^13^C). Coupling constants (*J*) were measured in Hertz (Hz). Elemental analyses (C, H, N) were carried out by a Vario III microanalyzer. The results obtained were within 0.4% of theoretical values. Thin-layer chromatography (TLC) was carried out on silica gel plates (Kieselgel 60 F254). Flash column chromatography was performed with Merck 60 silica gel (0.040–0.063 mm).

#### 3.1.2. Synthesis of Dimethyl-2(3*H*)-Benzothiazolones **31**–**33**, General Procedure

The corresponding 4-, 5-, or 6-methyl-2(3*H*)-benzothiazolone **28**–**30** (4.13 г; 25 mM) was dissolved in 10% aqueous solution of sodium hydroxide (20 mL). The solution was diluted with water to 200 mL, and dimethyl sulfate (4.8 mL, 51 mmol) was added. The reaction mixture was stirred at room temperature for 1 h and the obtained precipitate was filtered off. The product was purified by recrystallization from an appropriate solvent.

##### 3,4-Dimethyl-2(3*H*)-Benzothiazolone (**31**)

The compound was prepared from 4-methyl-2(3*H*)-benzothiazolone (**28**) following the general procedure in Section 3.1.2. Yield 89%. The product was purified by recrystallization from cyclohexane. Mp: 120–121 °C. Lit. Mp: 122–124 °C [71]. ^1^H NMR (CDCl_3_, 400 MHz): *δ* 2.72, (s, 3H, ArCH_3_), 3.79 (s, 3H, NCH_3_), 7.04–7.09 (m, 2H, ArH), 7.29 (m, 1H, ArH).

##### 3,5-Dimethyl-2(3*H*)-Benzothiazolone (**32**)

The compound was prepared from 5-methyl-2(3*H*)-benzothiazolone (**29**) following the general procedure in Section 3.1.2. Yield 80%. The product was purified by recrystallization from 50% ethanol. Mp: 122–124 °C. Lit. Mp: 121 °C [72]. ^1^H NMR (CDCl_3_, 500 MHz): *δ* 2.43, (s, 3H, ArCH_3_), 3.43 (s, 3H, NCH_3_), 6.86 (d, 1H, *J* = 0.7, ArH, 6.99 (dd, 1H, *J* = 0.7, 8.0, ArH), 7.29 (d, 1H, *J* = 8.0, ArH).

##### 3,6-Dimethyl-2(3*H*)-Benzothiazolone (**33**)

The compound was prepared from 6-methyl-2(3*H*)-benzothiazolone (**30**) following the general procedure in Section 3.1.2. Yield 81%. The product was purified by recrystallization from 50% ethanol. Mp: 71–73 °C. Lit. Mp: 76–77 °C [48]. ^1^H NMR (CDCl_3_, 500 MHz): *δ* 2.38, (s, 3H, ArCH_3_), 3.43 (s, 3H, NCH_3_), 6.92 (d, 1H, *J* = 8.2, ArH, 7.12 (dd, 1H, *J* = 0.8, 8.2, ArH), 7.23 (d, 1H, *J* = 0.8, ArH).

#### 3.1.3. Synthesis of Bromomethyl-3-Methyl-2(3*H*)-Benzothiazolones **34**–**36**, General Procedure

To a solution of the corresponding dimethyl-2(3*H*)-benzothiazolone **31**–**33** (5.38 g, 30 mmol) in carbon tetrachloride (150 mL), *N*-bromosuccinimide (5.52 g, 31 mmol), and dibenzoyl peroxide (0.28 g) were added. The reaction mixture was refluxed for 3 h and directly filtered off to remove participate from the succinimide. The filtrate was concentrated in vacuo and the obtained bromomethyl derivative was purified by recrystallization from an appropriate solvent.

##### 4-Bromomethyl-3-Methyl-2(3*H*)-Benzothiazolone (**34**)

The compound was prepared following the general procedure Section 3.1.3. for bromination. The product was purified by recrystallization from carbon tetrachloride. Yield 84%. Mp: 87–88 °C. ^1^H NMR (CDCl_3_, 400 MHz): δ 3.92 (s, 3H, NCH_3_), 4.80 (s, 2H, CH_2_Br), 7.10 (t, 1H, *J* = 7.8 Hz, ArH), 7.24 (dd, 1H, *J* = 1.1, *J* = 7.8 Hz, ArH), 7.37 (dd, 1H, *J* = 1.1, *J* = 7.8 Hz, ArH).

##### 5-Bromomethyl-3-Methyl-2(3*H*)-Benzothiazolone (**35**)

Compound **35** was prepared following the general procedure Section 3.1.3. for bromination. The product was purified by recrystallization from toluene. Yield 80%. Mp: 128–131 °C. ^1^H NMR (CDCl_3_, 500 MHz): δ 3.47 (s, 3H, NCH_3_), 4.55 (s, 2H, CH_2_Br), 7.07 (s, 1H, Hz, ArH), 7.20 (d, 1H, *J* = 7.2 Hz, ArH), 7.39 (m, 1H, ArH).

##### 6-Bromomethyl-3-Methyl-2(3*H*)-Benzothiazolone (**36**)

Compound **36** was prepared following the general procedure Section 3.1.3. for bromination. The product was purified by recrystallization from toluene. Yield 63%. Mp: 145–147 °C. Lit. Mp: 145–150 °C [73]. ^1^H NMR (CDCl_3_, 500 MHz): δ 3.45 (s, 3H, NCH_3_), 4.54 (s, 2H, CH_2_Br), 7.00 (d, 1H, *J* = 8.3, ArH), 7.36 (dd, 1H, *J* = 1.7, *J* = 8.3, ArH), 7.47 (d, 1H, *J* = 1.7, ArH).

#### 3.1.4. General Procedure for the Synthesis of Phosphonium Salts **11**–**13**

Triphenylphosphine (5.25 g, 20 mmol) was added to a solution of the corresponding bromomethyl derivative **34**–**36** (5.16 g, 20 mmol) in chlorobenzene (30 mL). The reaction mixture was heated to reflux for 15 min and then allowed to cool to room temperature. The obtained crystals were filtered off and washed with benzene. The phosphonium salts were used in the next stage without further purification.

##### [(3-Methyl-2(3*H*)-Benzothiazolone-4-yl)Methyl]Triphenylphosphonium Bromide (**11**)

Starting from bromomethyl derivative **34**, compound **11** was obtained as colorless crystals. Yield: 85%. Mp: 290 °C. ^1^H NMR (CDCl_3_, 400 MHz): *δ* 3.16 (s, 3H, NCH_3_), 5.82 (d, 2H, *J* = 13.4 Hz, PCH_2_), 6.78 (t, 1H, *J* = 7.6 Hz, ArH), 7.19–7.21 (m, 1H, ArH), 7.25–7.28 (m, 1H, ArH), 7.54–7.63 (m, 12H, ArH), 7.70–7.74 (m, 3H, ArH). Calcd. for C_27_H_23_BrNOPS: C 62.31; H 4.45; N 2.69. Found: C 61.98; H 4.13; N 2.33.

##### [(3-Methyl-2(3*H*)-Benzothiazolone-5-yl)Methyl]Triphenylphosphonium Bromide (**12**)

Starting from bromomethyl derivative **35**, compound **12** was obtained as colorless crystals. Yield: 93%. Mp: 305–307 °C. ^1^H NMR (DMSO-d_6_, 400 MHz): *δ* 3.04 (s, 3H, NCH_3_), 5.30 (d, 2H, *J* = 15.6 Hz, PCH_2_), 6.71 (br s, 1H, ArH), 6.93 (dd, 1H, *J* = 1.9, *J* = 8.0, ArH), 7.56 (d, 1H, *J* = 8.0, ArH), 7.71–7.77 (m, 12H, ArH), 7.90–7.93 (m, 3H, ArH). Calcd. for C_27_H_23_BrNOPS: C 62.31; H 4.45; N 2.69; S 6.16. Found: C 62.30; H 4.30; N 2.69; S 6.13.

##### [(3-Methyl-2(3*H*)-Benzothiazolone-6-yl)Methyl]Triphenylphosphonium Bromide (**13**)

Starting from bromomethyl derivative **36**, compound **13** was obtained as colorless crystals. Yield: 96%. Mp: 310–312 °C. ^1^H NMR (CDCl_3_, 400 MHz): *δ* 3.27 (s, 3H, NCH_3_), 5.78 (d, 2H, *J* = 14.5, PCH_2_), 6.64 (d, 1H, *J* = 8.3, ArH), 7.09 (d, 1H, *J* = 2.2, ArH), 7.20 (dd, 1H, *J* = 2.2, *J* = 8.3, ArH), 7.56–7.61 (m, 6H, ArH), 7.71–7.74 (m, 3H, ArH), 7.79–7.84 (m, 6H, ArH). Calcd. for C_27_H_23_BrNOPS: C 62.31; H 4.45; N 2.69; S 6.16. Found: C 62.34; H 4.31; N 2.70; S 6.17.

#### 3.1.5. 3-Methyl-2(3*H*)-Benzothiazolone-7-Carbaldehyde (**10**)

To a solution of 1.34 g (7.5 mmol) 2(3*H*)-benzothiazolone-7-carbaldehyde (**49**) in 10 mL DMF, 2.13 g (15 mmol) K_2_CO_3_ and 1.56 g (11 mmol) methyl iodide was added. The mixture was heated at 45 °C for 1 h and poured into water (50 mL). The obtained precipitate was filtered off and washed with water. Recrystallization from ethanol afforded 1.22 g product. Yield: 84%. Mp: 188–189 °C. ^1^H NMR (DMSO-d_6_, 500 MHz): *δ* 3.44 (s, 3H, NCH_3_), 7.63–7.67 (m, 2H, ArH), 7.88 (m, 1H, ArH), 10.13 (s, 1H, CHO). ^13^C NMR (DMSO-d_6_, 126 MHz): *δ* 29.0, 116.6, 120.0, 126.9, 128.6, 129.2, 138.9, 170.2, 192.5.

#### 3.1.6. General Procedure for the Stilbene Syntheses **15**–**27**

To a stirred solution of the corresponding phosphonium salt **11**–**14** (3 mmol) in THF/CH_2_Cl_2_ (15 mL, 2:1 *v*/*v*), powdered K_2_CO_3_ (10 mmol) and 18-crown-6 (0.01 g) were added, followed by the corresponding aldehyde **5**–**10** (3 mmol). The reaction mixture was refluxed for 4–6 h (monitored by TLC). After cooling, the inorganic salts were filtered off and washed with CH_2_Cl_2_. The filtrate was concentrated under reduced pressure and the mixture of the corresponding *E*/*Z*-stilbenes and triphenylphosphine oxide were isolated by flash column chromatography on silica gel using petroleum ether-AcOEt (9:1). The *Z*-stilbenes were eluted first, followed by the *E*-isomers.

##### (*E*/*Z*)-4-(4-Methoxystyryl)-3-Methyl-2(3*H*)-Benzothiazolone (**15**)

Following the general procedure Section 3.1.6, diastereomers **15Z** and **15E** were obtained by reaction of phosphonium salt **11** and 4-methoxybenzaldehyde (**5**). Separation by flash column chromatography afforded pure stilbenes **15Z** (40% yield) and **15E** (41% yield). Compound **15Z**: colorless oil that crystallizes over time. Mp: 100–102 °C. ^1^H NMR (CDCl_3_, 500 MHz): *δ* 3.66 (s, 3H, NCH_3_), 3.75 (s, 3H, OCH_3_), 6.66 (d, 1H, *J* = 12.0 Hz, =CH), 6.70 (d, 2H, J = 8.8 Hz, ArH), 6.78 (d, 1H, *J* = 12.0 Hz, =CH), 7.02–7.05 (m, 3H, ArH), 7.07 (d, 1H, *J* = 6.7 Hz, ArH), 7.33 (dd, 1H, *J* = 1.2 Hz, *J* =7.3 Hz, ArH). Calcd. for C_17_H_15_NO_2_S: C 68.66; H 5.08; N 4.71. Found: C 68.99; H 5.40; N 4.55. ^13^C NMR (CDCl_3_, 126 MHz): *δ* 32.7, 55.3, 113.9, 121.6, 123.2, 123.4, 123.6, 124.6, 128.6, 129.1, 130.6, 132.0, 135.4, 159.2, 170.6. Compound **15E**: white crystals. Mp: 122–123 °C. ^1^H NMR (CDCl_3_, 500 MHz): *δ* 3.72 (s, 3H, NCH_3_), 3.84 (s, 3H, OCH_3_), 6.80 (d, 1H, *J* = 15.9 Hz, =CH), 6.93 (d, 2H, J = 8.7 Hz, ArH), 7.13 (t, 1H, *J* = 7.8 Hz, ArH), 7.32–7.34 (m, 2H, ArH), 7.43 (d, 2H, *J* = 8.7 Hz, ArH), 7.46 (d, 1H, *J* = 15.9 Hz, =CH). ^13^C NMR (CDCl_3_, 126 MHz): *δ* 33.8, 55.5, 114.5, 121.6, 123.0, 123.1, 123.2, 124.9, 127.4, 127.9, 129.7, 132.8, 135.3, 159.9, 170.8. Calcd. for C_17_H_15_NO_2_S: C 68.66; H 5.08; N 4.71. Found: C 68.83; H 5.11; N 4.62.

##### (*E*/*Z*)-4-(3,4-Dimethoxystyryl)-3-Methyl-2(3*H*)-Benzothiazolone (**16**)

Following the general procedure Section 3.1.6, diastereomers **16Z** and **16E** were obtained by reaction of phosphonium salt **11** and 3,4-dimethoxybenzaldehyde (**6**). Separation by flash column chromatography afforded pure stilbenes **16Z** (38% yield) and **16E** (46% yield). Compound **16Z**: colorless oil. ^1^H NMR (CDCl_3_, 500 MHz): *δ* 3.45 (s, 3H, OCH_3_), 3.64 (s, 3H, NCH_3_), 3.82 (s, 3H, OCH_3_), 6.51 (d, 1H, *J* = 1.4 Hz, ArH), 6.64 (d, 1H, *J* = 12.0 Hz, =CH), 6.70–6.71 (m, 2H, ArH), 6.80 (d, 1H, *J* = 12.0 Hz, =CH), 7.08 (t, 1H, *J* = 7.6 Hz, ArH), 7.12 (d, 1H, *J* = 7.0 Hz, ArH), 7.33 (dd, 1H, *J* = 1.0 Hz, *J* =7.4 Hz, ArH). ^13^C NMR (CDCl_3_, 126 MHz): *δ* 32.8, 55.4, 55.9, 110.9, 111.3, 121.6, 122.8, 123.2, 123.4, 123.6, 124.6, 128.8, 129.1, 132.5, 135.4, 148.6, 114.8, 170.5. Calcd. for C_18_H_17_NO_3_S: C 66.03; H 5.23; N 4.28. Found: C 66.31; H 5.57; N 4.03. Compound **16E**: white crystals. Mp: 154–156 °C. ^1^H NMR (CDCl_3_, 500 MHz): *δ* 3.72 (s, 3H, NCH_3_), 3.92 (s, 3H, OCH_3_), 3.94 (s, 3H, OCH_3_), 6.79 (d, 1H, *J* = 15.9 Hz, =CH), 6.89 (d, 1H, *J* = 8.3 Hz, ArH), 7.01 (d, 1H, *J* = 1.9 Hz, ArH), 7.07 (dd, 1H, *J* = 1.9 Hz, *J* = 8.3 Hz, ArH), 7.13 (t, 1H, *J* = 7.8 Hz, ArH), 7.33 (d, 1H, *J* = 7.8 Hz, ArH), 7.45 (d, 1H, *J* = 15.8 Hz, =CH). ^13^C NMR (CDCl_3_, 126 MHz): *δ* 33.8, 56.0, 56.1, 109.4, 111.5, 119.7, 121.7, 123.1, 123.2, 123.4, 124.8, 127.4, 130.0, 133.1, 135.3, 149.4, 149.5, 170.8.

##### (*E*/*Z*)-4-(3,5-Dimethoxystyryl)-3-methyl-2(3*H*)-benzothiazolone (**17**)

Following the general procedure Section 3.1.6, diastereomers **17Z** and **17E** were obtained by reaction of phosphonium salt **11** and 3,5-dimethoxybenzaldehyde (**7**). Separation by flash column chromatography afforded pure stilbenes **17Z** (41% yield) and **17E** (43% yield). Compound **17Z**: colorless oil. ^1^H NMR (CDCl_3_, 600 MHz): *δ* 3.55 (s, 6H, OCH_3_), 3.64 (s, 3H, NCH_3_), 6.22 (d, 2H, *J* = 2.3 Hz, ArH), 6.28 (t, 1H, *J* = 2.3 Hz, ArH), 6.65 (d, 1H, *J* = 12.0 Hz, =CH), 6.92 (d, 1H, *J* = 12.0 Hz, =CH), 7.06–7.09 (m, 2H, ArH), 7.33–7.36 (m, 1H, ArH). ^13^C NMR (CDCl_3_, 151 MHz): *δ* 32.8, 55.2, 100.5, 107.1, 121.7, 123.2, 123.2, 123.3, 127.1, 129.0, 132.8, 135.3, 137.6, 160.6, 170.5. Calcd. for C_18_H_17_NO_3_S: C 66.03; H 5.23; N 4.28. Found: C 66.40; H 5.41; N 4.17. Compound **17E**: white crystals. Mp: 143–144 °C. ^1^H NMR (CDCl_3_, 400 MHz): *δ* 3.72 (s, 3H, NCH_3_), 3.84 (s, 6H, OCH_3_), 6.44 (t, 2H, *J* = 2.2 Hz, ArH), 6.64 (d, 2H, *J* = 2.2 Hz, ArH), 6.78 (d, 1H, *J* = 15.8, =CH), 7.14 (t, 1H, *J* = 7.8, ArH), 7.33–7.36 (m, 2H, ArH), 7.58 (d, 1H, *J* = 15.8, =CH).

##### (*E*/*Z*)-3-Methyl-4-(3,4,5-Trimethoxystyryl)-2(3*H*)-Benzothiazolone (**18**)

Following the general procedure Section 3.1.6, diastereomers **18Z** and **18E** were obtained by reaction of phosphonium salt **11** and 3,4,5-trimethoxybenzaldehyde (**8**). Separation by flash column chromatography afforded pure stilbenes **18Z** (35% yield) and **18E** (39% yield). Compound **18Z**: colorless oil. ^1^H NMR (CDCl_3_, 500 MHz): *δ* (CDCl_3_, 250 MHz): δ 3.55 (s, 6H, OCH_3_), 3.64 (s, 3H, NCH_3_), 3.79 (s, 3H, OCH_3_), 6.29 (s, 2H, ArH), 6.63 (d, 1H, *J* = 12.0, =CH,), 6.89 (d, 1H, *J* = 12.0, =CH,), 7.10–7.13 (m, 2H, ArH), 7.33–7.36 (m, 2H, ArH). ^13^C NMR (CDCl_3_, 151 MHz): *δ* 32.8, 55.8, 61.0, 106.3, 121.7, 123.3, 123.4, 123.4, 125.9, 128.9, 131.3, 132.7, 135.3, 137.8, 153.0, 170.4. Calcd. for C_19_H_19_NO_4_S: C 63.85; H 5.36; N 3.92. Found: C 63.97; H 5.65; N 3.59. Compound **18E**: white crystals. Mp: 159–160 °C. ^1^H NMR (CDCl_3_, 500 MHz): *δ* 3.72 (s, 3H, NCH_3_), 3.88 (s, 3H, OCH_3_), 3.92 (s, 6H, OCH_3_), 6.71 (s, 2H, ArH), 6.77 (d, 1H, *J* = 15.8, =CH), 7.14 (t, 1H, ArH), 7.31–7.36 (m, 2H, ArH), 7.49 (d, 1H, *J* = 15.8, =CH,). Calcd. for C_19_H_19_NO_4_S: C 63.85; H 5.36; N 3.92. Found: C 64.02; H 5.47; N 3.73.

##### (*E*/*Z*)-5-(4-Methoxystyryl)-3-Methyl-2(3*H*)-Benzothiazolone (**19**)

Following the general procedure Section 3.1.6, diastereomers **19Z** and **19E** were obtained by reaction of phosphonium salt **12** and 4-methoxybenzaldehyde (**5**). Separation by flash column chromatography afforded pure stilbenes **19Z** (36% yield) and **19E** (34% yield). Compound **19Z**: white crystals. Mp: 117–118 °C. ^1^H NMR (CDCl_3_, 600 MHz): *δ* 3.28 (s, 3H, NCH_3_), 3.79 (s, 3H, OCH_3_), 6.50 (d, 1H, *J* = 12.1, =CH), 6.61 (d, 1H, *J* = 12.1, =CH), 6.78 (d, 2H, *J* = 8.8, ArH), 6.98 (br s, 1H, ArH), 7.08 (dd, 1H, *J* = 1.1, *J* = 8.1, ArH), 7.19 (d, 2H, *J* = 8.8, ArH), 7.28 (d, 1H, *J* = 8.1, ArH). ^13^C NMR (CDCl_3_, 151 MHz): *δ* 29.0, 55.4, 110.7, 113.8, 121.1, 122.4, 124.3, 128.0, 129.3, 130.3, 130.8, 136.2, 137.8, 159.0, 170.3. Calcd. for C_17_H_15_NO_2_S: C 68.66; H 5.08; N 4.71; S 10.78. Found: C 69.03; H 5.11; N 4.76; S 11.16. Compound **19E**: white crystals. Mp: 178–180 °C. ^1^H NMR (CDCl_3_, 400 MHz): *δ* 3.49 (s, 3H, NCH_3_), 3.84 (s, 3H, OCH_3_), 6.92 (d, 2H, *J* = 8.7, ArH), 6.99 (d, 1H, *J* = 16.3, =CH), 7.10 (d, 1H, *J* = 16.3, =CH), 7.13 (d, 1H, *J* = 1.1, ArH), 7.30 (dd, 1H, *J* = 1.1, *J* = 8.2, ArH), 7.38 (d, 1H, *J* = 8.1, ArH), 7.47 (d, 2H, *J* = 8.7, ArH). Calcd. for C_17_H_15_NO_2_S: C 68.66; H 5.08; N 4.71; S 10.78. Found: C 68.92; H 5.07; N 4.92; S 10.59.

##### (*E*/*Z*)-5-(3,4-Dimethoxystyryl)-3-Methyl-2(3*H*)-Benzothiazolone (**20**)

Following the general procedure Section 3.1.6, diastereomers **20Z** and **20E** were obtained by reaction of phosphonium salt **12** and 3,4-dimethoxybenzaldehyde (**6**). Separation by flash column chromatography afforded pure stilbenes **20Z** (36% yield) and **20E** (32% yield). Compound **20Z**: colorless oil. ^1^H NMR (CDCl_3_, 250 MHz): *δ* 3.30 (s, 3H, NCH_3_), 3.67 (s, 3H, OCH_3_), 3.86 (s, 3H, OCH_3_), 6.53 (d, 1H, *J* = 12.2, =CH), 6.61 (d, 1H, *J* = 12.2, =CH), 6.75 (d, 1H, *J* = 8.2, ArH), 6.78 (d, 1H, *J* = 1.8, ArH), 6.83 (dd, 1H, *J* = 1.8, *J* = 8.3, ArH), 6.94 (d, 1H, *J* = 1.5, ArH), 7.12 (dd, 1H, *J* = 1.4, *J* = 8.3, ArH), 7.28 (d, 1H, *J* = 8.1, ArH). ^13^C NMR (CDCl_3_, 126 MHz): *δ* 29.0, 55.8, 56.0, 110.8, 111.1, 111.9, 121.2, 121.9, 122.3, 124.3, 128.2, 129.6, 131.0, 136.2, 137.8, 148.6, 148.7, 170.2. Calcd. for C_18_H_17_NO_3_S: C 66.03; H 5.23; N 4.28; S 9.79. Found: C 66.23; H 5.27; N 3.98; S 9.72. Compound **20E**: white crystals. Mp: 132–134 °C. ^1^H NMR (CDCl_3_, 400 MHz): *δ* 3.50 (s, 3H, NCH_3_), 3.92 (s, 3H, OCH_3_), 3.96 (s, 3H, OCH_3_), 6.88 (d, 1H, *J* = 8.2, ArH), 6.99 (d, 1H, *J* = 16.3, =CH), 7.06–7.08 (m, 2H, ArH), 7.09 (d, 1H, *J* = 16.2, =CH), 7.14 (br s, 1H, ArH), 7.31 (dd, 1H, *J* = 1.3, *J* = 8.1, ArH), 7.38 (d, 1H, *J* = 8.1, ArH). Calcd. for C_18_H_17_NO_3_S: C 66.03; H 5.23; N 4.28; S 9.79. Found: C 66.40; H 5.22; N 4.36; S 9.80.

##### (*E*/*Z*)-5-(3,5-Dimethoxystyryl)-3-Methyl-2(3*H*)-Benzothiazolone (**21**)

Following the general procedure Section 3.1.6, diastereomers **21Z** and **21E** were obtained by reaction of phosphonium salt **12** and 3,5-dimethoxybenzaldehyde (**7**). Separation by flash column chromatography afforded pure stilbenes **21Z** (35% yield) and **21E** (33% yield). Compound **21Z**: white crystals. Mp: 98–100 °C. ^1^H NMR (CDCl_3_, 250 MHz): *δ* 3.27 (s, 3H, NCH_3_), 3.68 (s, 6H, OCH_3_), 6.35 (t, 1H, *J* = 2.3, ArH), 6.42 (d, 2H, *J* = 2.3, ArH), 6.58 (d, 1H, *J* = 12.3, =CH), 6.63 (d, 1H, *J* = 12.3, =CH), 6.93 (d, 1H, *J* = 1.5, ArH), 7.09 (dd, 1H, *J* = 1.5, *J* = 8.1, ArH), 7.28 (d, 1H, *J* = 8.1, ArH). ^13^C NMR (CDCl_3_, 151 MHz): *δ* 29.0, 55.4, 99.8, 106.8, 110.9, 121.5, 122.5, 124.5, 129.8, 131.1, 135.6, 137.7, 139.0, 160.9, 170.3. Calcd. for C_18_H_17_NO_3_S: C 66.03; H 5.23; N 4.28; S 9.79. Found: C 66.42; H 5.35; N 4.54; S 9.85. Compound **21E**: white crystals. Mp: 128–130 °C. ^1^H NMR (CDCl_3_, 250 MHz): *δ* 3.49 (s, 3H, NCH_3_), 3.84 (s, 6H, OCH_3_), 6.42 (t, 1H, *J* = 2.2, ArH), 6.68 (d, 2H, *J* = 2.2, ArH), 7.04 (d, 1H, *J* = 16.3, =CH), 7.11 (d, 1H, *J* = 16.3, =CH), 7.14 (d, 1H, *J* = 1.5, ArH), 7.31 (dd, 1H, *J* = 1.5, *J* = 8.1, ArH), 7.39 (d, 1H, *J* = 8.1, ArH). Calcd. for C_18_H_17_NO_3_S: C 66.03; H 5.23; N 4.28; S 9.79. Found: C 65.73; H 5.07; N 4.26; S 10.10.

##### (*E*/*Z*)-3-Methyl-5-(3,4,5-Trimethoxystyryl)-2(3*H*)-Benzothiazolone (**22**)

Following the general procedure Section 3.1.6, diastereomers **22Z** and **22E** were obtained by reaction of phosphonium salt **12** and 3,4,5-trimethoxybenzaldehyde (**8**). Separation by flash column chromatography afforded pure stilbenes **22Z** (27% yield) and **22E** (30% yield). Compound **22Z**: white crystals. Mp: 131–133 °C. ^1^H NMR (CDCl_3_, 600 MHz): *δ* 3.29 (s, 3H, NCH_3_), 3.66 (s, 6H, OCH_3_), 3.82 (s, 3H, OCH_3_), 6.47 (s, 2H, ArH), 6.55 (d, 1H, *J* = 12.2, =CH), 6.58 (d, 1H, *J* = 12.2, =CH), 6.92 (d, 1H, *J* = 1.4, ArH), 7.11 (dd, 1H, *J* = 1.4, *J* = 8.1, ArH), 7.28 (d, 1H, *J* = 8.1, ArH). ^13^C NMR (CDCl_3_, 151 MHz): *δ* 29.0, 56.1, 61.1, 106.0, 110.8, 121.4, 122.2, 124.4, 129.1, 131.1, 132.4, 135.8, 137.6, 137.8, 153.2, 170.3. Calcd. for C_19_H_19_NO_4_S: C 63.85; H 5.36; N 3.92; S 8.97. Found: C 63.45; H 5.27; N 3.98; S 8.72. Compound **22E**: white crystals. Mp: 172–173 °C. ^1^H NMR (CDCl_3_, 400 MHz): *δ* 3.48 (s, 3H, NCH_3_), 3.86 (s, 3H, OCH_3_), 3.91 (s, 6H, OCH_3_), 6.74 (s, 2H, ArH), 7.01 (d, 1H, *J* = 16.4, =CH), 7.05 (d, 1H, *J* = 16.4, =CH), 7.13 (d, 1H, *J* = 1.3, ArH), 7.30 (dd, 1H, *J* = 1.3, *J* = 8.1 ArH), 7.37 (d, 1H, *J* = 8.1, ArH). Calcd. for C_19_H_19_NO_4_S: C 63.85; H 5.36; N 3.92; S 8.97. Found: C 63.50; H 5.25; N 3.98; S 8.83.

##### (*E*/*Z*)-6-(4-Methoxystyryl)-3-Methyl-2(3*H*)-Benzothiazolone (**23**)

Following the general procedure Section 3.1.6, diastereomers **23Z** and **23E** were obtained by reaction of phosphonium salt **13** and 4-methoxybenzaldehyde (**5**). Separation by flash column chromatography afforded pure stilbenes **23Z** (36% yield) and **23E** (35% yield). Compound **23Z**: white crystals. Mp: 139–141 °C. ^1^H NMR (CDCl_3_, 400 MHz): *δ* 3.43 (s, 3H, NCH_3_), 3.80 (s, 3H, OCH_3_), 6.47 (d, 1H, *J* = 12.1, =CH), 6.55 (d, 1H, *J* = 12.1, =CH), 6.77 (d, 2H, *J* = 8.8, ArH), 6.90 (d, 1H, *J* = 8.3, ArH), 7.17 (d, 2H, *J* = 8.8, ArH), 7.23 (dd, 1H, *J* = 1.3, *J* = 8.3, ArH), 7.33 (d, 1H, *J* = 1.3, ArH). ^13^C NMR (CDCl_3_, 151 MHz): *δ* 29.1, 55.3, 110.3, 113.9, 122.6, 122.8, 127.4, 127.6, 129.4, 130.1, 130.2, 133.1, 136.6, 158.9, 170.2. Calcd. for C_17_H_15_NO_2_S: C 68.66; H 5.08; N 4.71; S 10.78. Found: C 69.01; H 5.15; N 4.74; S 10.66. Compound **23E**: white crystals. Mp: 196–197 °C. ^1^H NMR (CDCl_3_, 400 MHz): *δ* 3.46 (s, 3H, NCH_3_), 3.84 (s, 3H, OCH_3_), 6.91 (d, 2H, *J* = 8.8, ArH), 6.96–7.01 (m, 2H, ArH + =CH), 7.01 (d, 1H, *J* = 16.2, =CH), 7.42–7.45 (m, 3H, ArH), 7.56 (d, 1H, *J* = 1.5, ArH). Calcd. for C_17_H_15_NO_2_S: C 68.66; H 5.08; N 4.71; S 10.78. Found: C 69.02; H 5.08; N 4.78; S 10.46.

##### (*E*/*Z*)-6-(3,4-Dimethoxystyryl)-3-Methyl-2(3*H*)-Benzothiazolone (**24**)

Following the general procedure Section 3.1.6, diastereomers **24Z** and **24E** were obtained by reaction of phosphonium salt **13** and 3,4-dimethoxybenzaldehyde (**6**). Separation by flash column chromatography afforded pure stilbenes **24Z** (46% yield) and **24E** (39% yield). Compound **24Z**: white crystals. Mp: 94–96 °C. ^1^H NMR (CDCl_3_, 600 MHz): *δ* 3.42 (s, 3H, NCH_3_), 3.66 (s, 3H, OCH_3_), 3.87 (s, 3H, OCH_3_), 6.48 (d, 1H, *J* = 12.1, =CH), 6.54 (d, 1H, *J* = 12.1, =CH), 6.75 (d, 1H, *J* = 8.2, ArH), 6.80–6.82 (m, 2H, ArH), 6.90 (d, 1H, *J* = 8.3, ArH), 7.25 (dd, 1H, *J* = 1.1, *J* = 8.4, ArH), 7.38 (s, 1H, ArH). ^13^C NMR (CDCl_3_, 151 MHz): *δ* 29.2, 55.8, 55.9, 110.2, 111.0, 111.7, 121.9, 122.5, 122.8, 127.4, 127.7, 129.6, 130.2, 133.0, 136.6, 148.5, 148.6, 170.1. Calcd. for C_18_H_17_NO_3_S: C 66.03; H 5.23; N 4.28; S 9.79. Found: C 65.93; H 5.48; N 4.20; S 10.05. Compound **24E**: white crystals. Mp: 167–169 °C. ^1^H NMR (CDCl_3_, 400 MHz): *δ* 3.46 (s, 3H, NCH_3_), 3.91 (s, 3H, OCH_3_), 3.95 (s, 3H, OCH_3_), 6.87 (d, 1H, *J* = 8.0, ArH), 6.94 (d, 1H, *J* = 16.2, =CH), 6.99–7.06 (m, 4H, ArH + =CH), 7.44 (dd, 1H, *J* = 1.5, *J* = 8.4, ArH), 7.58 (d, 1H, *J* = 1.5, ArH). Calcd. for C_18_H_17_NO_3_S: C 66.03; H 5.23; N 4.28; S 9.79. Found: 66.38; H 5.23; N 4.32; S 9.83.

##### (*E*/*Z*)-6-(3,5-Dimethoxystyryl)-3-Methyl-2(3*H*)-Benzothiazolone (**25**)

Following the general procedure Section 3.1.6, diastereomers **25Z** and **25E** were obtained by reaction of phosphonium salt **13** and 3,5-dimethoxybenzaldehyde (**7**). Separation by flash column chromatography afforded pure stilbenes **25Z** (49% yield) and **25E** (36% yield). Compound **25Z**: white crystals. Mp: 75–77 °C. ^1^H NMR (CDCl_3_, 600 MHz): *δ* 3.42 (s, 3H, NCH_3_), 3.66 (s, 6H, OCH_3_), 6.34 (t, 1H, *J* = 2.3, ArH), 6.41 (d, 2H, *J* = 2.3, ArH), 6.53 (d, 1H, *J* = 12.5, =CH), 6.55 (d, 1H, *J* = 12.5, =CH), 6.89 (d, 1H, *J* = 8.3, ArH), 7.24 (dd, 1H, *J* = 1.7, *J* = 8.3, ArH), 7.35 (d, 1H, *J* = 1.7, ArH). ^1^H NMR (acetone-d6, 250 MHz): *δ* 3.42 (s, 3H, NCH_3_), 3.65 (s, 6H, OCH_3_), 6.36 (t, 1H, *J* = 2.3, ArH), 6.42 (d, 2H, *J* = 2.3, ArH), 6.56 (d, 1H, *J* = 12.2, =CH), 6.63 (d, 1H, *J* = 12.2, =CH), 7.14 (d, 1H, *J* = 8.4, ArH), 7.30 (dd, 1H, *J* = 1.7, *J* = 8.4, ArH), 7.47 (d, 1H, *J* = 1.7, ArH). ^13^C NMR (CDCl_3_, 151 MHz): *δ* 29.9, 55.4, 99.9, 106.8, 110.2, 122.5, 123.0, 127.6, 129.4, 130.3, 132.5, 136.8, 138.9, 160.8, 170.2. Calcd. for C_18_H_17_NO_3_S: C 66.03; H 5.23; N 4.28; S 9.79. Found: C 66.25; H 5.60; N 4.09; S 9.40. Compound **25E**: white crystals. Mp: 145–147 °C. ^1^H NMR (CDCl_3_, 250 MHz): *δ* 3.46 (s, 3H, NCH_3_), 3.83 (s, 6H, OCH_3_), 6.40 (t, 1H, *J* = 2.2, ArH), 6.65 (d, 2H, *J* = 2.2, ArH), 6.93–7.02 (m, 2H, ArH + =CH), 7.06 (d, 1H, *J* = 16.3, =CH), 7.45 (dd, 1H, *J* = 1.7, *J* = 8.4, ArH), 7.58 (d, 1H, *J* = 1.7, ArH). Calcd. for C_18_H_17_NO_3_S: C 66.03; H 5.23; N 4.28; S 9.79. Found: 66.23; H 5.38; N 4.39; S 10.01.

##### (*E*/*Z*)-3-Methyl-6-(3,4,5-Trimethoxystyryl)-2(3*H*)-Benzothiazolone (**26**)

*Path A*. Following the general procedure Section 3.1.6, diastereomers **26Z** and **26E** were obtained by reaction of phosphonium salt **13** and 3,4,5-trimethoxybenzaldehyde (**8**). Separation by flash column chromatography afforded pure stilbenes **26Z** (53% yield) and **26E** (34% yield). *Path B*. Following the general procedure 4.1.6., diastereomers **26Z** and **26E** were obtained by reaction of (3,4,5-trimethoxybenzyl)triphenylphosphonium bromide (**14**) [74] and 3-methyl-2(3*H*)-benzothiazolone-6-carbaldehyde (**9**) [35]. Separation by flash column chromatography afforded pure stilbenes **26Z** (40% yield) and **26E** (36% yield). Compound **26Z**: white crystals. Mp: 115–117 °C. ^1^H NMR (CDCl_3_, 500 MHz): *δ* 3.43 (s, 3H, NCH_3_), 3.68 (s, 6H, OCH_3_), 3.84 (s, 3H, OCH_3_), 6.49 (s, 2H, ArH), 6.52 (s, 2H, =CH), 6.91 (d, 1H, *J* = 8.3, ArH), 7.26 (dd, 1H, *J* = 1.5, *J* = 8.3, ArH), 7.40 (d, 1H, *J* = 1.4, ArH). ^13^C NMR (CDCl_3_, 126 MHz): *δ* 29.2, 56.1, 61.1, 106.1, 110.1, 122.5, 123.0, 127.6, 128.7, 130.3, 132.4, 132.7, 136.8, 137.6, 153.2, 170.1. ^1^H NMR (acetone-d6, 500 MHz): *δ* 3.43 (s, 3H, NCH_3_), 3.63 (s, 6H, OCH_3_), 3.71 (s, 3H, OCH_3_), 6.54 (d, 1H, *J* = 11.3, =CH), 6.58 (s, 2H, ArH), 6.60 (d, 1H, *J* = 12.2, =CH), 7.18 (d, 1H, *J* = 8.4, ArH), 7.33 (dd, 1H, *J* = 1.7, *J* = 8.4, ArH), 7.52 (d, 1H, *J* = 1.6, ArH). Calcd. for C_19_H_19_NO_4_S: C 63.85; H 5.36; N 3.92; S 8.97. Found: C 64.22; H 5.31; N 3.95; S 8.57. Compound **26E**: white crystals. Mp: 167–168 °C. ^1^H NMR (CDCl_3_, 400 MHz): *δ* 3.46 (s, 3H, NCH_3_), 3.87 (s, 3H, OCH_3_), 3.92 (s, 6H, OCH_3_), 6.73 (s, 2H, ArH), 6.98 (s, 2H, =CH), 7.02 (d, 1H, *J* = 8.4, ArH), 7.45 (dd, 1H, *J* = 1.6, *J* = 8.4, ArH), 7.59 (d, 1H, *J* = 1.6, ArH). ^1^H NMR (acetone-d6, 250 MHz): *δ* 3.46 (s, 3H, NCH_3_), 3.74 (s, 3H, OCH_3_), 3.87 (s, 6H, OCH_3_), 6.91 (s, 2H, ArH), 7.15 (d, 1H, *J* = 16.4, =CH), 7.21 (d, 1H, *J* = 8.5, ArH), 7.23 (d, 1H, *J* = 16.4, =CH), 7.57 (dd, 1H, *J* = 1.7, *J* = 8.4, ArH), 7.80 (d, 1H, *J* = 1.7, ArH). Calcd. for C_19_H_19_NO_4_S: C 63.85; H 5.36; N 3.92; S 8.97. Found: C 64.10; H 5.35; N 3.98; S 8.82.

##### (*E*/*Z*)-3-Methyl-7-(3,4,5-Trimethoxystyryl)-2(3*H*)-Benzothiazolone (**27**)

Following the general procedure Section 3.1.6, diastereomers **27Z** and **27E** were obtained by reaction of (3,4,5-trimethoxybenzyl)triphenylphosphonium bromide (**14**) and 3-methyl-2(3*H*)-benzothiazolone-7-carbaldehyde (**10**). Separation by flash column chromatography afforded pure stilbenes **27Z** (39% yield) and **27E** (42% yield). Compound **27Z**: white crystals. Mp: 155–156 °C. ^1^H NMR (CDCl_3_, 500 MHz): *δ* 3.45 (s, 3H, NCH_3_), 3.60 (s, 6H, OCH_3_), 3.81 (s, 3H, OCH_3_), 6.41 (s, 2H, ArH), 6.48 (d, 1H, *J* = 12 Hz, =CH), 6.65 (d, 1H, *J* = 12 Hz, =CH), 6.90 (d, 1H, *J* = 7.9 Hz, ArH), 7.15 (d, 1H, *J* = 7.8 Hz, ArH), 7.23 (t, 1H, *J* = 7.9 Hz, ArH). ^13^C NMR (CDCl_3_, 126 MHz): *δ* 22.3, 55.9, 61.0, 106.2, 108.9, 122.5, 123.5, 125.7, 126.1, 131.6, 132.6, 133.2, 137.9, 138.2, 153.0, 170.1. Calcd. for C_19_H_19_NO_4_S: C 63.85; H 5.36; N 3.92. Found: C 64.19; H 5.54; N 3.79. Compound **27E**: white crystals. Mp: 178–180 °C. ^1^H NMR (CDCl_3_, 500 MHz): *δ* 3.48 (s, 3H, NCH_3_), 3.68 (s, 3H, OCH_3_), 3.93 (s, 6H, OCH_3_), 6.75 (s, 2H, ArH), 6.91–6.96 (m, 2H, ArH + =CH), 7.05 (d, 1H, *J* = 16.1 Hz, =CH), 7.33–7.36 (m, 1H, ArH), 7.37 (dd, 1H, *J* = 1.4, *J* = 7.9 Hz, ArH), 7.37 (d, 1H, *J* = 8.1 Hz, ArH). ^13^C NMR (CDCl_3_, 126 MHz): *δ* 29.4, 56.4, 61.2, 104.1, 109.4, 120.9, 121.2, 124.8, 126.6, 132.2, 132.3, 132.4, 138.5, 138.8, 153.4, 169.8. Calcd. for C19H19NO4S: C 63.85; H 5.36; N 3.92. Found: C 63.67; H 5.15; N 3.81.

### 3.2. Crystallography and Docking

#### 3.2.1. General

Suitable single crystals (with the appropriate size and without cracks) of the corresponding compounds **19Z**, **22Z**, **22E**, **23Z**, **24Z**, **25Z**, **26E**, and **27Z** were mounted on glass capillaries. The coordinates and intensities of the diffraction peaks for **19Z**, **23Z 24Z**, **25Z**, and **27Z** were collected on an Agilent SuperNova Dual diffractometer equipped with an Atlas CCD detector using micro-focus Mo*K*α radiation (λ = 0.71073 Å), while those for compounds **22Z**, **22E**, and **26E** were collected on an Enraf Nonius CAD4 diffractometer equipped with graphite monochromator and scintillation LiI detector. All structures were solved by direct methods and refined by the full-matrix least-squares method on *F*^2^ with ShelxS and ShelxL programs [75] using Olex2 software [76]. The non-hydrogen atoms were located successfully from the Fourier map and were refined anisotropically. All hydrogen atoms were placed on calculated positions using the riding model (Ueq = 1.2 for C-H_aromatic_, C-H_etylenic_ = 0.93 Å and C-H_methyl_ = 0.96 Å). The most important crystallographic parameters from the X-ray diffraction experiment and selected bond lengths, angles, and torsion angles are given in Tables S2.1. and S2.2.

#### 3.2.2. General Molecular Docking of the **22Z**, **27Z**, **26Z**, and **25Z** Derivatives in the Colchicine Binding Site of Tubulin

The docking and three-dimensional representations of predicted ligand-protein interactions were performed using Molegro Virtual Docker (v6.0.1) software [77]. As a starting model, we used the crystal structure of tubulin with colchicine as a ligand (PDB code: 1SA0 [54]). The identification of the cavity (after removing the colchicine) for the potential binding of ligands **22Z**, **27Z**, **26Z**, and **25Z** at the border between the α and β tubulin subunits was performed automatically using the grid-based cavity prediction algorithm. During the docking simulation, the backbone was kept rigid, but the torsional angles in the side chains of amino acids close to the detected cavity were allowed to change. The MolDockSE algorithm (1500 iterations) together with MolDock Grid scoring function (grid resolution of 0. 30 Å) were used to determine the best possible poses [77].

### 3.3. Biology

#### 3.3.1. Cell Cultures

The endothelial cell line (EA.hy926) was kindly provided by Dr. C-J.S. Edgell—University of North Carolina, USA. Cells were cultured in 4.5 g/L D-Glucose DMEM/F12 medium, supplemented with 10% fetal bovine serum (FBS, Cytogen GmBH, Greven, Germany) and 100 U/mL penicillin and streptomycin (Lonza BioWhitaker, Verviers, Belgium). The other cell lines used are described in Table S3.1. Cell cultures were maintained in an incubator at 37 °C with 95% humidity and 5% CO_2_ and were routinely found free of mycoplasma.

#### 3.3.2. Cellular Treatment

All tested compounds were dissolved in DMSO at 25 mM initial concentration and diluted with medium to obtain the working concentrations (0.01 nM–250 µM). Serial drug dilutions were prepared in medium immediately before each experiment. The DMSO working concentration in the medium was 0.01 µM. For the MTT assay, cells were seeded into 96-well microtitre plates at a density of 1 × 10^4^ in 100 µL complete medium per well. They were further incubated for 24 h, followed by treatment with CA-4 analogs up to 72 h. Cell viability was determined using the MTT assay [78]. Ten µL of MTT solution (5 mg/mL) was added to each well and further incubated for 3 h at 37 °C. To dissolve the formazan product, 100 µL/well of 99.5% isopropanol was added. Cell viability was determined by measuring absorbance at 550/630 nm using a DTX880 plate reader (Beckman Coulter, USA). Results, expressed as concentrations that inhibit 20% (IC_20_), 50% (IC_50_), and 80% (IC_80_) of cell growth, were calculated using GraphPad Prism 8.0.1.

#### 3.3.3. Clonogenic Survival Assay

EA.hy926 cells were seeded at 300 cells/well on a 6-well plate and incubated for 24 h. The cells were treated with different concentrations of test compounds for 72 h. After six additional days of growth in a fresh medium to form visible colonies, cells were washed with phosphate-buffered saline (PBS, pH 7.4) and stained with crystal violet (0.5%, *w*/*v*) in 6% glutaraldehyde. After 30 min of incubation, the plates were washed with tap water, dried, photographed, and analyzed using ImageJ [79]. Raw data were normalized to the corresponding DMSO control for each independent experiment. After normalization, statistically significant differences for colony-forming efficiency (CFE) values versus 0.01 µM DMSO were calculated.

#### 3.3.4. Western Blot Analysis

A total of 7 × 10^5^ cells was seeded in 100-mm dishes. On the next day, cells were treated with the corresponding concentrations of CA-4 and **26Z**. After 24 h, treated cells were collected and lysed with lysis buffer (50 mM HEPES, pH 7.7, 250 mM KCl, 10% glycerol, 0.1% Nonidet P-40) containing protease inhibitors (0.4 mM sodium vanadate, 0.5 mM PMSF, 1 mM DTT, 2 mg/mL leupeptin, and 2 mg/mL pepstatin) by incubation on ice for 30 min and overnight at −20 °C [80]. The concentration of obtained total cell protein lysates was determined by the method of Bradford [81]. Samples were separated on 12% polyacrylamide gels and transferred to nitrocellulose or PVDF membranes. Immunoblotting was performed by blocking in 3.5% non-fat dried milk in PBS, with subsequent incubation for 1 h with primary antibodies for caspase 3 (sc-56053, Santa Cruz Biotechnology), caspase 8 (sc-81656, Santa Cruz Biotechnology), caspase 9 (sc-8355, Santa Cruz Biotechnology), and PARP-1 (sc-8007, Santa Cruz Biotechnology), diluted in PBS containing 0.05% Tween-20. This was followed by incubation with a peroxidase-conjugated secondary antibody m-Igk BP-HRP (sc-516102, Santa Cruz Biotechnology) or a donkey anti-rabbit IgG-HRP (sc-2313, Santa Cruz Biotechnology) for 1 h, and detection of the chemiluminescent signal was accomplished with ECL Select^TM^ Western Blotting Detection Reagent (Amersham) on X-ray film. β–actin was used as a loading control and mouse monoclonal antibody was used following the manufacturer’s instruction (sc-47778, Santa Cruz Biotechnology).

PARP-1 was incubated with a secondary IRDye^®^ 800CW goat anti-mouse IgG (H + L) (LI-COR Biosciences) antibody and imaged with an Odyssey Imaging System (LI-COR Biosciences).

#### 3.3.5. In Vitro Cell Migration Assays

A total of 3 × 10^5^ EA.hy926 cells were seeded in a 12-well plate and grown close to the confluence. The cell monolayer was gently scraped with a P200 pipette tip, creating a wound area around 1-mm wide. Cells were washed twice with fresh medium and treated with test compounds diluted in growth media to the corresponding sub-toxic concentrations. Images were acquired at 0 and 24 h following treatment. After incubation for 24 h, the scratch wound width was measured and recorded, and then compared with the initial scratch wound width at 0 h. Using the ImageJ image processing program (ImageJ150-WIN-Java8), the size of the denuded area in pixels was determined at each time point from the digital images.

#### 3.3.6. Matrigel Tube Disruption Assay

The tube formation assay was performed in 96-well plates coated with 40 µL BD Matrigel™ (basement membrane matrix), following the manufacturer’s instruction (BD Biosciences). A total of 2 × 10^4^ EA.hy926 cells was re-suspended in growth medium and added to each well in a final volume of 100 µL. Tube formation was visualized directly using a light microscope. Images were captured using a phase-contrast microscope and quantitation was performed using the WimTube application of the Wimasis online image analysis platform (https://www.wimasis.com/en/products/13/WimTube; accessed on 1 June 2021).

#### 3.3.7. Ex Vivo Tubulin Polymerization Assays

##### Ex Vivo Unlabeled Tubulin Polymerization

α,β-tubulin powder (T240, Cytoskeleton) was diluted in PEM buffer (80 mM PIPES, 2 mM MgCl_2_, 0.5 mM EGTA, pH 6.9) on ice to a final concentration of 4 mg/mL. Five μL of α,β-tubulin was mixed with 0.75 μL 50% glycerol, and 0.7 μL 200 μM solution of the substances studied. Polymerization was started by adding 0.55 μL 10 mM GTP, and the samples were incubated at 35 °C for 45 min, followed by centrifugation at 13,000 × g for 10 min. The supernatant was separated and mixed with 5× sample buffer (5% SDS, 45% glycerol, 12.5% β-mercaptoethanol, and 0.05% bromophenol blue in 200 mM Tris-HCl, pH 6.8). The pellet was re-suspended in 1× sample buffer followed by separation on 10% sodium dodecyl sulfate-polyacrylamide gel electrophoresis (SDS-PAGE). Western blot analysis was performed as described in Section 3.3.4 with the primary anti-β-tubulin antibody (sc-101527, Santa Cruz Biotechnology) diluted to 1:1000, followed by incubation with a secondary donkey anti-mouse 680 antibody (LI-COR Biosciences) and imaging with an Odyssey Imaging System (LI-COR Biosciences). The fluorescence intensity was analysed with Image Studio Lite v. 5.2.

##### Colchicine-Binding Site Assay

*N*,*N*′-ethylene-bis(iodoacetamide) (abcam, ab144980) was dissolved in DMSO to a final concentration of 50 mM. EA.hy926 cells were seeded at density of 1 × 10^5^ cells/mL and incubated overnight. Cells were treated with vehicle control 10 μM DMSO, 10 μM colchicine, 10 μM CA-4, 25 μM vinblastine, or different concentrations of **26Z** for 2 h, then 100 μM EBI was added and the cells were further incubated for another 2 h. Cells were lysed with Laemmli buffer and subjected to Western blot analysis for β-tubulin [66,67]. The detailed Western blot method is as shown above.

##### Ex Vivo Rhodamin-Tubulin Polymerization

TAMRA-Tubulin (TL590M, Cytoskeleton) diluted in GTB buffer (PEM, 10% glycerol, 1 mM GTP) was mixed with unlabeled α,β-tubulin (T240, Cytoskeleton) at a ratio of 1:3 (*v*/*v*) to a concentration of 4.0 mg/mL. Tubulin polymerization was performed at 37°C for 45 min in the presence of 5% glycerol and 20 μM of test compounds (0.08% DMSO, **26Z**, CA-4, and paclitaxel), and it was initiated with the addition of 1 mM GTP. Samples were then centrifuged for 20 min at 30,000× *g*. The pellets were diluted in 6 µL PEM buffer, containing 1 mM GTP and 0.5% glutaraldehyde, mixed with anti-fade mounting media (P36934, Invitrogen, Eugene, USA), and examined under a confocal LSM 980 Airyscan microscope (Carl Zeiss AG, Oberkochen, Germany).

#### 3.3.8. In Vitro Quantification of Polymeric vs. Soluble Tubulin Fractions

To assess the early effects on the microtubule cytoskeleton in vitro, EA.hy926 cells were cultured for 24 h in 6-cm dishes (3 × 10^5^ cells). They were exposed to 0.01 µM DMSO (control), 1 μM CA-4, paclitaxel, or increasing concentrations of **26Z** (50, 100, 250, 2500, 10,000 nM) for 6 h. Cells were washed with PBS and lysed directly in the dishes with hypotonic lysis buffer (20 mM Tris-HCl, 1 mM MgCl_2_, 2 mM EGTA, 0.5% Triton X-100, glycerol 10%, pH 6.8), supplemented with protease inhibitors (2 μg/mL leupeptin, 2 μg/mL pepstatin, 0.4 mM Na vanadate, 0.5 mM PMSF, 1 mM DTT), for 3 min at 30 °C. The soluble fraction was immediately collected, mixed with 5× sample buffer, and boiled at 95 °C for 5 min. The polymerized fraction was collected directly from the culture dish following 10 min of incubation at room temperature with 1× sample buffer [82].

For the concentration-dependent assay, the cells were harvested by scraping and centrifuged at 1000 g for 5 min, and then lysed with the hypotonic cell lysis buffer for 10 min at room temperature [83]. Tubulin fractions were obtained by centrifugation at 12,000 × g for 10 min.

Equal volumes of the samples were subjected to 10% SDS-PAGE followed by western blotting. Detection of β-tubulin content was accomplished with an anti-β-tubulin mouse monoclonal antibody (sc-101527, Santa Cruz Biotechnology).

#### 3.3.9. Cell Cycle Analysis

A total of 5 × 10^5^ cells was treated with **26Z** and CA-4 for time-response assays. Cells were collected by scraping, pelleted, washed with cold PBS (1×), and fixed overnight at 4 °C with 1 mL of ice-cold methanol–acetone (1:1), added dropwise to the pellet while vortexing. Pellets were resuspended and washed in 500 μL of PBS (1×), then permeabilized with a Triton X-100 (0.05–0.1%)/DNA extraction buffer (0.2 M Na_2_HPO_4_, 0.04 M citric acid, pH 7.8) in a 1:1 ratio for 15 min. After that, pelleted cells were treated with RNAse A (20 μg/mL) and labeled with 10 μg/mL of propidium iodide (Sigma-Aldrich) by incubation at 37 °C for at least 30 min, protected from light. DNA content was measured using a FACS Calibur flow cytometer (Beckman Dickenson, NJ, USA), and cell cycle analysis was performed with FlowJo software (version 8.7). At least 10,000 events were recorded for each sample measured in duplicate.

#### 3.3.10. Immunocytochemistry

Indirect immunofluorescence was used to examine the effect of **26Z** on α-tubulin in EA.hy 926 cells. A total of 1 × 10^4^ cells grown on coverslips were washed with PBS and fixed with 3.7% buffered paraformaldehyde. Single fluorescence cell labeling was performed, as described by Apostolova et al. [84], with a primary chicken antibody against α-tubulin (ab89984, Abcam) for 2 h. Cells were washed three times with PBS for 5 min and incubated for 60 min with a goat anti-chicken secondary antibody labeled with AlexaFluor-488 (ab150173, Abcam). F-actin was detected using AlexaFluor-568 Phalloidin (Invitrogen, MA, USA). Following three washes with PBS and two in water, the slides were mounted in UltraCruz fluorescence mounting medium with DAPI (sc-24941, Santa Cruz Biotechnology, USA). Fluorescence microscopy was performed with a Carl Zeiss Axiovert 200 M Inverted Microscope and Andor Dragonfly 505 Confocal Microscope equipped with an iXon Ultra 888 EMCCD camera (Andor, Oxford Instruments, Belfast, Northern Irelands).

#### 3.3.11. Statistical Analysis

The data were evaluated by analysis of variance (ANOVA) followed by Tukey’s post-hock test. Differences in the results at the level of *p* < 0.05 were considered statistically significant. The statistical analysis was carried out using the PASW 18.0 statistical software package (IBM) for Windows.

## 4. Conclusions

Here we describe the synthesis, characterization, and biological activities of a series of 26 cis- and trans-styryl-benzothiazolones. Our results showed that the number of methoxy-groups in ring A and the position of the styryl fragment benzothiazolone heterocycle (4-, 5-, 6- or 7) were crucial for the biological activity of the obtained compounds **15Z**–**27Z**. Among the reported CA-4 analogs, compound **26Z**, (*Z*)-3-methyl-6-(3,4,5-trimethoxystyryl)-2(3*H*)-benzothiazolone, exhibited the most potent anti-proliferative effect. We have demonstrated that its primary mechanism of action ex vivo is the inhibition of β-tubulin polymerization. In vitro activity is associated with changes in the soluble/polymerized α,β-tubulin ratio, thus favoring the soluble form present in the endothelial cells.

Additionally, the in silico studies demonstrated that these molecules could interact with tubulin in the areas of the colchicine binding site, suggesting a similar mechanism of action. The three most active compounds, **25Z**, **26Z**, and **27Z**, induced destabilization of microtubules, cell cycle arrest, mitotic catastrophe, and cell death. We also showed that **26Z** inhibits a spectrum of angiogenic events in EA.hy926 cells by interfering with endothelial cell invasion, migration, and neovascularization. Undoubtedly, future work is warranted to further explore the chemotherapeutic potential of these new CA-4 analogs.

## Figures and Tables

**Table 1:** Structure of *E*/*Z* styryl-benzothiazolones **15**–**27**.

Compound	Ring A			Position in Ring B	Yield (%)
	R^1^	R^2^	R^3^		
**15Z** **15E**	H	OCH_3_	H	4	81
**16Z** **16E**	OCH_3_	OCH_3_	H	4	84
**17Z** **17E**	OCH_3_	H	OCH_3_	4	84
**18Z** **18E**	OCH_3_	OCH_3_	OCH_3_	4	74
**19Z** **19E**	H	OCH_3_	H	5	70
**20Z** **20E**	OCH_3_	OCH_3_	H	5	68
**21Z** **21E**	OCH_3_	H	OCH_3_	5	68
**22Z** **22E**	OCH_3_	OCH_3_	OCH_3_	5	57
**23Z** **23E**	H	OCH_3_	H	6	71
**24Z** **24E**	OCH_3_	OCH_3_	H	6	85
**25Z** **25E**	OCH_3_	H	OCH_3_	6	85
**26Z** **26E**	OCH_3_	OCH_3_	OCH_3_	6	87/76 ^a^
**27Z** **27E**	OCH_3_	OCH_3_	OCH_3_	7	81

^a^ Yield Path A/Path B.

**Table 2:** Growth inhibition of endothelial EA.hy926 cells by benzothiazolone CA-4 analogs after 72 h of treatment.

Compound	IC_50_ ^a^ ± SE (µM)	Compound	IC_50_ ± SE (µM)
**15Z**	22.03 ± 4.57	**22Z**	9.43 ± 0.92
**16Z**	25.78 ± 3.11	**23Z**	19.76 ± 3.45
**17Z**	19.50 ± 2.71	**24Z**	9.28 ± 1.13
**18Z**	15.64 ± 1.16	**25Z**	1.88 ± 0.18
**19Z**	11.96 ± 2.49	**26Z**	0.13 ± 0.01
**20Z**	14.58 ± 1.75	**26E**	38.20 ± 5.35
**21Z**	10.77 ± 0.62	**27Z**	0.30 ± 0.05
		**CA-4**	0.0033 ± 0.0002

**^a^** Drug concentration that inhibits the growth of treated cells by 50% at 72 h. Each drug concentration was tested in eight replicates of three independent syntheses, and IC_50_ values were determined as the average ± SE.

## Data Availability

The data is contained in article and Supplementary Materials.

## Supplementary Materials

The following are available online at https://www.mdpi.com/article/10.3390/ph14121331/s1. S.1.1. General information on chemistry; S.1.2. Synthesis of 4-methyl-2(3*H*)-benzothiazolone (**28**); S.1.3.1. Synthesis of 5-Methylbenzothiazole-2-thiol (**40**); S.1.3.2. Synthesis of 5-Methyl-2(3*H*)-benzothiazolone (**29**); S.1.4.1. Synthesis of 6-Methylbenzothiazole-2-thiol (**44**); S.1.4.2. Synthesis of 2-Bromo-6-methylbenzothiazole (**46**); S.1.4.3. Synthesis of 6-Methyl-2(3*H*)-benzothiazolone (**30**); Table S2.1. The most important crystallographic parameters for the crystal structures; Table S2.2. Selected bond lengths, angles, and torsion angles for the structures; Table S2.3. Potential hydrogen bonding interactions for the structures; Table S2.4. Angles between normals, twist and fold angles for **25Z**, **22Z,** and **27Z**; Figure S2.1. ORTEP view of the molecules in the asymmetric unit of the crystal structures of **19Z**; Figure S2.2. ORTEP view of the molecules in the asymmetric unit of the crystal structures of **22E**; Figure S2.3. ORTEP view of the molecules in the asymmetric unit of the crystal structures of **26E**; Figure S2.4. ORTEP view of the molecules in the asymmetric unit of the crystal structures of **23Z**; Figure S2.5. ORTEP view of the molecules in the asymmetric unit of the crystal structures of **24Z**; Table S3.1. Description of eukaryotic cell lines used for MTT assay; Table S3.2. IC_50_ values for CA-4 and **26Z** against different tumor and control cell lines following treatment for 72 h; Figure S3.1. Dose-response curves of CA-4 (▲) and **26Z** (□) in various tumor cell lines; Figure S3.2. Effect of 26Z and CA-4 on the cell cycle in EA.hy926 cells; Figure S4.1.1. ^1^H-NMR (CDCl_3_) of compound **15Z**; Figure S4.1.2. ^13^C-NMR (CDCl_3_) of compound **15Z**; Figure S4.2.1. ^1^H-NMR (CDCl_3_) of compound **16Z**; Figure S4.2.2. ^13^C-NMR (CDCl_3_): (*Z*)-4-(3,4-Dimethoxystyryl)-3-methyl-2(3*H*)-benzothiazolone (1**6Z**); Figure S4.3.1. ^1^H-NMR (CDCl_3_) of compound **17Z**; Figure S4.3.2. ^13^C-NMR (CDCl_3_): (*Z*)-4-(3,5-Dimethoxystyryl)-3-methyl-2(3*H*)-benzothiazolone (**17Z**); Figure S4.4.1. ^1^H-NMR (CDCl_3_) of compound **18Z**; Figure S4.4.2. ^13^C-NMR (CDCl_3_): (*Z*)-3-Methyl-4-(3,4,5-trimethoxystyryl)-2(3*H*)-benzothiazolone (**18Z**); Figure S4.5.1. ^1^H-NMR (CDCl_3_) of compound **19Z**; Figure S4.5.2. ^13^C-NMR (CDCl_3_): (*Z*)-5-(4-Methoxystyryl)-3-methyl-2(3*H*)-benzothiazolone (**19Z**); Figure S4.6.1. ^1^H-NMR (CDCl_3_) of compound **20Z**; Figure S4.6.2. ^13^C-NMR (CDCl_3_): (*Z*)-5-(3,4-Dimethoxystyryl)-3-methyl-2(3*H*)-benzothiazolone (**20Z**); Figure S4.7.1. ^1^H-NMR (CDCl_3_) of compound **21Z**; Figure S4.7.2. ^13^C-NMR (CDCl_3_): (*Z*)-5-(3,5-Dimethoxystyryl)-3-methyl-2(3*H*)-benzothiazolone (**21Z**); Figure S4.8.1. ^1^H-NMR (CDCl_3_) of compound **22Z**; Figure S4.8.2. ^13^C-NMR (CDCl_3_): (*Z*)-3-Methyl-5-(3,4,5-trimethoxystyryl)-2(3*H*)-benzothiazolone (**22Z**); Figure S4.9.1. ^1^H-NMR (CDCl_3_) of compound **23Z**; Figure S4.9.2. ^13^C-NMR (CDCl_3_): (*Z*)-6-(4-Methoxystyryl)-3-methyl-2(3*H*)-benzothiazolone (**23Z**); Figure S4.10.1. ^1^H-NMR (CDCl_3_) of compound **24Z**; Figure S4.10.2. ^13^C-NMR (CDCl_3_): (*Z*)-6-(3,4-Dimethoxystyryl)-3-methyl-2(3*H*)-benzothiazolone (**24Z**); Figure S4.11.1. ^1^H-NMR (CDCl_3_) of compound **25Z**; Figure S4.11.2. ^1^H-NMR (acetone-d_6_) of compound **25Z**; Figure S4.11.3. ^13^C-NMR (CDCl_3_): (*Z*)-6-(3,5-Dimethoxystyryl)-3-methyl-2(3*H*)-benzothiazolone (**25Z**); Figure S4.12.1. ^1^H-NMR (CDCl_3_) of compound **26Z**; Figure S4.12.2. ^1^H-NMR (acetone-d_6_) of compound **26Z**; Figure S4.12.3. ^13^C-NMR (CDCl_3_) of compound **26Z**; Figure S4.13.1. ^1^H-NMR (CDCl_3_) of compound **26E**; Figure S4.13.2. ^1^H-NMR (acetone-d_6_) of compound **26E**; Figure S4.14.1. ^1^H-NMR (CDCl_3_) of compound **27Z**; Figure S4.14.2. ^13^C-NMR (CDCl_3_) of compound **27Z**. References [36,37,40,42,45,46,47,48] are cited in the Supplementary Materials.

## References

[1] Goodson H.V., Jonasson E.M. (2018). Microtubules and Microtubule-Associated Proteins. Cold Spring Harb. Perspect. Biol..

[2] Kaur R., Kaur G., Gill R.K., Soni R., Bariwal J. (2014). Recent developments in tubulin polymerization inhibitors: An overview. Eur. J. Med. Chem..

[3] La Regina G., Coluccia A., Naccarato V., Silvestri R. (2019). Towards modern anticancer agents that interact with tubulin. Eur. J. Pharm. Sci..

[4] Ong M.S., Deng S., Halim C.E., Cai W., Tan T.Z., Huang R.Y.-J., Sethi G., Hooi S.C., Kumar A.P., Yap C.T. (2020). Cytoskeletal Proteins in Cancer and Intracellular Stress: A Therapeutic Perspective. Cancers.

[5] Naaz F., Haider M.R., Shafi S., Yar M.S. (2019). Anti-tubulin agents of natural origin: Targeting taxol, vinca, and colchicine binding domains. Eur. J. Med. Chem..

[6] Pettit G.R., Singh S.B., Hamel E., Lin C.M., Alberts D.S., Garcia-Kendall D. (1989). Isolation and structure of the strong cell growth and tubulin inhibitor combretastatin A-4. Experientia.

[7] Duan Y., Liu W., Tian L., Mao Y., Song C. (2019). Targeting Tubulin-colchicine Site for Cancer Therapy: Inhibitors, Antibody-Drug Conjugates and Degradation Agents. Curr. Top. Med. Chem..

[8] McLoughlin E.C., O’Boyle N.M. (2020). Colchicine-Binding Site Inhibitors from Chemistry to Clinic: A Review. Pharmaceuticals.

[9] Liu Y., Wang S., Zhao X., Feng Y., Bormans G., Swinnen J., Oyen R., Huang G., Ni Y., Li Y. (2020). Predicting Clinical Efficacy of Vascular Disrupting Agents in Rodent Models of Primary and Secondary Liver Cancers: An Overview with Imaging-Histopathology Correlation. Diagnostics.

[10] Nagaiah G., Remick S.C. (2010). Combretastatin A4 phosphate: A novel vascular disrupting agent. Future Oncol..

[11] Yin T., Yin T., Liu Y., Peeters R., Feng Y., Yu J., Himmelreich U., Oyen R., Ni Y. (2017). Vascular disrupting agent in pancreatic and hepatic tumour allografts: Observations of location-dependent efficacy by MRI, microangiography and histomorphology. Br. J. Cancer.

[12] Field J.J., Kanakkanthara A., Miller J.H. (2014). Microtubule-targeting agents are clinically successful due to both mitotic and interphase impairment of microtubule function. Bioorg. Med. Chem..

[13] Siemann D.W., Chaplin D.J., Walicke P.A. (2009). A review and update of the current status of the vasculature-disabling agent combretastatin-A4 phosphate (CA4P). Expert Opin. Investig. Drugs.

[14] Grisham R., Ky B., Tewari K.S., Chaplin D.J., Walker J. (2018). Clinical trial experience with CA4P anticancer therapy: Focus on efficacy, cardiovascular adverse events, and hypertension management. Gynecol. Oncol. Res. Pract..

[15] Jaroch K., Karolak M., Górski P., Jaroch A., Krajewski A., Ilnicka A., Sloderbach A., Stefański T., Sobiak S. (2016). Combretastatins: In vitro structure-activity relationship, mode of action and current clinical status. Pharmacol. Rep..

[16] Pettit G.R., Toki B.E., Herald D.L., Boyd M.R., Hamel E., Pettit R.K., Chapuis J.C. (1999). Antineoplastic agents. 410. Asymmetric hydroxylation of trans-combretastatin A-4. J. Med. Chem..

[17] Malebari A.M., Wang S., Greene T.F., O’Boyle N.M., Fayne D., Khan M.F., Nathwani S.M., Twamley B., McCabe T., Zisterer D.M. (2021). Synthesis and Antiproliferative Evaluation of 3-Chloroazetidin-2-ones with Antimitotic Activity: Heterocyclic Bridged Analogues of Combretastatin A-4. Pharmaceuticals.

[18] Romagnoli R., Oliva P., Salvador M.K., Manfredini S., Padroni C., Brancale A., Ferla S., Hamel E., Ronca R., Maccarinelli F. (2021). A facile synthesis of diaryl pyrroles led to the discovery of potent colchicine site antimitotic agents. Eur. J. Med. Chem..

[19] Gerova M.S., Stateva S.R., Radonova E.M., Kalenderska R.B., Rusew R.I., Nikolova R.P., Chanev C.D., Shivachev B.L., Apostolova M.D., Petrov O.I. (2016). Combretastatin A-4 analogues with benzoxazolone scaffold: Synthesis, structure and biological activity. Eur. J. Med. Chem..

[20] Ibrahim T.S., Hawwas M.M., Malebari A.M., Taher E.S., Omar A.M., O’Boyle N.M., McLoughlin E., Abdel-Samii Z.K., Elshaier Y.A.M.M. (2020). Potent Quinoline-Containing Combretastatin A-4 Analogues: Design, Synthesis, Antiproliferative, and Anti-Tubulin Activity. Pharmaceuticals.

[21] Takahashi S., Nakano K., Yokota T., Shitara K., Muro K., Sunaga Y., Ecstein-Fraisse E., Ura T. (2016). Phase 1 study of ombrabulin in combination with cisplatin (CDDP) in Japanese patients with advanced solid tumors. Jpn. J. Clin. Oncol..

[22] Gao M., Zhang D., Jin Q., Jiang C., Wang C., Li J., Peng F., Huang D., Zhang J., Song S. (2016). Combretastatin-A4 phosphate improves the distribution and antitumor efficacy of albumin-bound paclitaxel in W256 breast carcinoma model. Oncotarget.

[23] Morgan R.D., Banerjee S., Hall M., Clamp A.R., Zhou C., Hasan J., Orbegoso C., Taylor S., Tugwood J., Lyon A.R. (2020). Pazopanib and Fosbretabulin in recurrent ovarian cancer (PAZOFOS): A multi-centre, phase 1b and open-label, randomised phase 2 trial. Gynecol. Oncol..

[24] Ljubas J., Ovesen T., Rusan M.A. (2019). Systematic Review of Phase II Targeted Therapy Clinical Trials in Anaplastic Thyroid Cancer. Cancers.

[25] Monk B.J., Sill M.W., Walker J.L., Darus C.J., Sutton G., Tewari K.S., Martin L.P., Schilder J.M., Coleman R.L., Balkissoon J. (2016). Randomized Phase II Evaluation of Bevacizumab Versus Bevacizumab Plus Fosbretabulin in Recurrent Ovarian, Tubal, or Peritoneal Carcinoma: An NRG Oncology/Gynecologic Oncology Group Study. J. Clin. Oncol..

[26] Irfan A., Batool F., Zahra Naqvi S.A., Islam A., Osman S.M., Nocentini A., Alissa S.A., Supuran C.T. (2020). Benzothiazole derivatives as anticancer agents. J. Enzyme Inhib. Med. Chem..

[27] Rouf A., Tanyeli C. (2015). Bioactive thiazole and benzothiazole derivatives. Eur. J. Med. Chem..

[28] Chander Sharma P., Sharma D., Sharma A., Bansal K.K., Rajak H., Sharma S., Thakur V.K. (2020). New horizons in benzothiazole scaffold for cancer therapy: Advances in bioactivity, functionality, and chemistry. Appl. Mater Today.

[29] Pettit G.R., Singh S.B., Boyd M.R., Hamel E., Pettit R.K., Schmidt J.M., Hogan F. (1995). Antineoplastic agents. 291. Isolation and synthesis of combretastatins A-4, A-5, and A-6(1a). J. Med. Chem..

[30] Chen Y., Zou Y., Sun H.-Y., Liu X.-K., Xiao C.-F., Sun J., He S.-J., Li J. (2011). Practical and green synthesis of combretastatin A-4 and its prodrug CA4P using renewable biomass-based starting material. Synthesis.

[31] Gaukroger K., Hadfield J.A., Hepworth L.A., Lawrence N.J., McGown A.T. (2001). Novel syntheses of cis and trans isomers of combretastatin A-4. J. Org. Chem..

[32] Camacho-Dávila A.A. (2008). Kumada–Corriu Cross Coupling Route to the Anti-Cancer Agent Combretastatin A-4. Synth. Commun..

[33] Petrov O.I., Gerova M.S., Chanev C.D., Petrova K.V. (2011). New efficient synthesis of combretastatin A-4 via colvin rearrangement. Synthesis.

[34] Boden R. (1975). A mild method for preparing trans-alkenes: Crown ether catalysis of the Wittig reaction. Synthesis.

[35] Petrov O.I., Kalcheva V.B., Antonova A.T. (1997). C-Formylation of some 2(3*H*)-benzazolones and 2*H*-1,4-benzoxazin-3(4*H*)-one. Coll. Czechoslov. Chem. Commun..

[36] Zhou B., Hong H., Wang H., Zhang T., Han L., Zhu N. (2018). Efficient synthesis of benzothiazolone derivatives by a domino reaction of disulfide and COS under mild conditions. Eur. J. Org. Chem..

[37] Gerova M.S., Svetoslavov F.E., Shivachev B.L., Nikolova R.P., Petrov O.I. (2017). Synthesis of 4-acetyl-2(3*H*)-benzothiazolone: Sulfur bioisostere of benzoxazolone allelochemicals. Phosphorus Sulfur Silicon Relat. Elem..

[38] Barnikow G., Bödeker J. (1967). Über die S-N-Bindung, I. Bis-guanyl-disulfid-Salze als Zwischenstufe der Bildung von 1.2. 4-Thiadiazolidinen und 2-Amino-benzthiazolen. Chem. Ber..

[39] Dunbrook R., Zimmermann M. (1934). A Method for the Preparation of 2-Mercaptobenzothiazole1. J. Am. Chem. Soc..

[40] Teppema J., Sebrell B. (1927). Researches on mercaptothiazoles. I. J. Am. Chem. Soc..

[41] Efros L.S., Dawidenkow L.R. (1951). Obtaining 2-benzothiazole sulfonic acid. Zh. Obsh Khim..

[42] Dekhane D.V., Pawar S.S., Gupta S.V., Shingare M.S., Thore S.N. (2011). Synthesis of benzimidazolones, benzooxazolones, 2-amino-benzothiazoles from ethyl cyanoformate and o-phenylene diamines, o-aminophenols, oaminothiophenols promoted by lithium bromide. Lett. Org. Chem..

[43] Furniss B.S. (2003). Vogel’s Textbook of Practical Organic Chemistry.

[44] Chaudhuri N.C. (1996). Convenient strategies for the preparation of modified 2 (3 H)-benzothiazolethiones. Synth. Commun..

[45] Franchini C., Muraglia M., Corbo F., Florio M.A., Di Mola A., Rosato A., Matucci R., Nesi M., van Bambeke F., Vitali C. (2009). Synthesis and biological evaluation of 2-mercapto-1,3-benzothiazole derivatives with potential antimicrobial activity. Arch. Pharm..

[46] Hrobárik P., Hrobáriková V., Sigmundová I., Zahradník P., Fakis M., Polyzos I., Persephonis P. (2011). Benzothiazoles with tunable electron-withdrawing strength and reverse polarity: A route to triphenylamine-based chromophores with enhanced two-photon absorption. J. Org. Chem..

[47] Matevosyan R.O., Abramova N.I., Donskikh O.B. (1968). Chemistry of free radicals of the hydrazine series. LIV. Synthesis and study of the properties of α-(6-methylbenzothyazolyl-2)-α-phenyl-β-picrylhydrazyl and α-(2-methylbenzothyazolyl-6)-α-phenyl-β-picryhydrazyl. J. Org. Chem. USSR Engl. Transl..

[48] Hunter F.R., Parken R.E. (1935). The unsaturation and tautomeric mobility of heterocyclic compounds. Part VI. The mobility of 5-substituted 1-hydroxybenzthiazoles, and the ultra-violet absorption of mobile and static derivatives of 1-hydroxybenzthiazole. J. Chem. Soc..

[49] Stanetty P., Krumpak B. (1996). Novel synthesis of benzothiazole derivatives via directed lithiation and aryne-mediated cyclization followed by quenching with electrophiles. J. Org. Chem..

[50] Farrugia L.J. (2012). WinGX and ORTEP for Windows: An update. J. Appl. Crystallogr..

[51] Aydın A., Akkurt M., Önkol T., Büyükgüngör O. (2006). 3-[6-(2-Chlorobenzoyl)-2-oxo-2,3-dihydro-1,3-benzothiazol-3-yl] propanenitrile. Acta Crystallogr. Sect. E Struct. Rep. Online.

[52] Dölling W., Kischkies K., Heinemann F., Hartung H. (1993). Synthesen von α, β-ungesättigten 2-(2-Oxo-benzazolin-3-yl)-3-hydroxy-dithio-carbonsäure-alkylestern und entsprechend substituierten Keten-S, S-acetalen sowie deren Kristall-und Molekülstruktur. Monatsh. Chem..

[53] Chantrapromma S., Suwunwong T., Boonnak N., Fun H.-K. (2013). (2*E*)-1-(Pyridin-2-yl)-3-(2,4,5-trimethoxyphenyl) prop-2-en-1-one. Acta Crystallogr. Sect. E Struct. Rep. Online.

[54] Ravelli R.B., Gigant B., Curmi P.A., Jourdain I., Lachkar S., Sobel A., Knossow M. (2004). Insight into tubulin regulation from a complex with colchicine and a stathmin-like domain. Nature.

[55] Long F., Nicholls R.A., Emsley P., Grazulis S., Merkys A., Vaitkus A., Murshudov G.N. (2017). AceDRG: A stereochemical description generator for ligands. Acta Crystallogr. Sect. D Biol. Crystallogr..

[56] Mahal K., Biersack B., Schruefer S., Resch M., Ficner R., Schobert R., Mueller T. (2016). Combretastatin A-4 derived 5-(1-methyl-4-phenyl-imidazol-5-yl)indoles with superior cytotoxic and anti-vascular effects on chemoresistant cancer cells and tumors. Eur. J. Med. Chem..

[57] Gaspari R., Prota A.E., Bargsten K., Cavalli A., Steinmetz M.O. (2017). Structural Basis of cis- and trans-Combretastatin Binding to Tubulin. Chem.

[58] Kwak Y.S., Joo S.H., Gansukh E., Mistry B.M., Keum Y.S. (2019). Synthesis and anticancer activities of polymethylenedioxy analogues of combretastatin A-2. Appl. Biol. Chem..

[59] Conesa-Milián L., Falomir E., Murga J., Carda M., Meyen E., Liekens S., Alberto Marco J. (2018). Synthesis and biological evaluation of carbamates derived from aminocombretastatin A-4 as vascular disrupting agents. Eur. J. Med. Chem..

[60] Hura N., Sawant A.V., Kumari A., Guchhait S.K., Panda D. (2018). Combretastatin-Inspired Heterocycles as Antitubulin Anticancer Agents. ACS Omega.

[61] Han J., Lim W., You D., Jeong Y., Kim S., Lee J.E., Shin T.H., Lee G., Park S. (2019). Chemoresistance in the Human Triple-Negative Breast Cancer Cell Line MDA-MB-231 Induced by Doxorubicin Gradient Is Associated with Epigenetic Alterations in Histone Deacetylase. J. Oncol..

[62] Franken N.A., Rodermond H.M., Stap J., Haveman J., Van Bree C. (2006). Clonogenic assay of cells in vitro. Nat. Protoc..

[63] Tarade D., Ma D., Pignanelli C., Mansour F., Simard D., van den Berg S., Gauld J., McNulty J., Pandey S. (2017). Structurally simplified biphenyl combretastatin A4 derivatives retain in vitro anti-cancer activity dependent on mitotic arrest. PLoS ONE.

[64] Ashraf M., Shaik T.B., Malik M.S., Syed R., Mallipeddi P.L., Vardhan M.V.P.S.V., Kamal A. (2016). Design and synthesis of cis-restricted benzimidazole and benzothiazole mimics of combretastatin A-4 as antimitotic agents with apoptosis inducing ability. Bioorg. Med. Chem. Lett..

[65] Romagnoli R., Prencipe F., Oliva P., Kimatrai Salvador M., Brancale A., Ferla S., Hamel E., Viola G., Bortolozzi R., Persoons L. (2020). Design, synthesis and biological evaluation of 2-alkoxycarbonyl-3-anilinoindoles as a new class of potent inhibitors of tubulin polymerization. Bioorg. Chem..

[66] Song J., Gao Q.L., Wu B.W., Zhu T., Cui X.X., Jin C.J., Wang S.Y., Wang S.H., Fu D.J., Liu H.M. (2020). Discovery of tertiary amide derivatives incorporating benzothiazole moiety as anti-gastric cancer agents in vitro via inhibiting tubulin polymerization and activating the Hippo signaling pathway. Eur. J. Med. Chem..

[67] Fortin S., Lacroix J., Côté M.-F., Moreau E., Petitclerc E., C-Gaudreault R. (2010). Quick and simple detection technique to assess the binding of antimicrotubule agents to the colchicine-binding site. Biol. Proced. Online.

[68] Merks R.M., Brodsky S.V., Goligorksy M.S., Newman S.A., Glazier J.A. (2006). Cell elongation is key to in silico replication of in vitro vasculogenesis and subsequent remodeling. Dev. Biol..

[69] Guidolin D., Vacca A., Nussdorfer G.G., Ribatti D. (2004). A new image analysis method based on topological and fractal parameters to evaluate the angiostatic activity of docetaxel by using the Matrigel assay in vitro. Microvasc. Res..

[70] Galmarini C.M., Martin M., Bouchet B.P., Guillen-Navarro M.J., Martínez-Diez M., Martinez-Leal J.F., Akhmanova A., Aviles P. (2018). Plocabulin, a novel tubulin-binding agent, inhibits angiogenesis by modulation of microtubule dynamics in endothelial cells. BMC Cancer.

[71] Umemura T., Morino H., Watanabe T., Uematsu T. (1981). Method for Preparing 4-Substituted-N-methylbenzothiazolone derivatives. https://patents.google.com/patent/US4293702A/en.

[72] Sexton W.A. (1939). Reactions of benzthiazole derivatives. Part I. The reactivity of methylthiol group in quaternary salts of 1-methylthiobenzthiazole. J. Chem. Soc..

[73] Bruneau A.R.P., Crawley G.C., Oldham K. (1993). Bicyclic Heterocyclic Derivatives as 5-lipoxygenase Inhibitors. https://patents.google.com/patent/US5179115A/en.

[74] Pettit G.R., Singh S.B., Niven M.L., Hamel E., Schmidt J.M. (1987). Isolation, structure, and synthesis of combretastatins A-1 and B-1, potent new inhibitors of microtubule assembly, derived from Combretum caffrum. J. Nat. Prod..

[75] Sheldrick G.M. (2008). A short history of SHELX. Acta Crystallogr. Sect. A Found. Crystallogr..

[76] Dolomanov O.V., Bourhis L.J., Gildea R.J., Howard J.A.K., Puschmann H. (2009). OLEX2: A complete structure solution, refinement and analysis program. J. Appl. Crystallogr..

[77] Thomsen R., Christensen M.H. (2006). MolDock: A new technique for high-accuracy molecular docking. J. Med. Chem..

[78] Mosmann T. (1983). Rapid colorimetric assay for cellular growth and survival: Application to proliferation and cytotoxicity assays. J. Immunol. Methods.

[79] Schneider C.A., Rasband W.S., Eliceiri K.W. (2012). NIH Image to ImageJ: 25 years of image analysis. Nat. Methods.

[80] D’Souza S.J., Pajak A., Balazsi K., Dagnino L. (2001). Ca^2+^ and BMP-6 signaling regulate E2F during epidermal keratinocyte differentiation. J. Biol. Chem..

[81] Bradford M.M. (1976). A rapid and sensitive method for the quantitation of microgram quantities of protein utilizing the principle of protein-dye binding. Anal. Biochem..

[82] Padmaja P., Rao G.K., Indrasena A., Reddy B.V.S., Patel N., Shaik A.B., Reddy N., Dubey P.K., Bhadra M.P. (2015). Synthesis and biological evaluation of novel pyrano [3, 2-c] carbazole derivatives as antitumor agents inducing apoptosis via tubulin polymerization inhibition. Org. Biomol. Chem..

[83] Wang S., Zhang S., Zhao Z., Zhang C., Yang X., Wang Y. (2017). Connexin 43 enhances paclitaxel cytotoxicity in colorectal cancer cell lines. Exp. Ther. Med..

[84] Apostolova M.D., Cherian M.G. (2000). Delay of M-phase onset by aphidicolin can retain the nuclear localization of zinc and metallothionein in 3T3-L1 fibroblasts. J. Cell. Physiol..

