# Supplementary material for: New Heterocyclic Combretastatin A-4 Analogs: Synthesis and Biological Activity of Styryl-2(3H)-benzothiazolones
Source: Pharmaceuticals (Basel). 2021 Dec 20;14(12):1331. doi: 10.3390/ph14121331 (PMC8703450; doi:10.3390/ph14121331)
Supplement: Supplementary file 1 [file pharmaceuticals-14-01331-s001.zip › pharmaceuticals-1498278-supplementary.pdf]

Supplementary materials

Contents

S1. Chemistry………………………………………………………………………... 5

S.1.1. General information…………………………………………………………….. 5

S.1.2. Synthesis of 4-methyl-2(3*H*)-benzothiazolone (**28**)……………………………….. 5

S.1.3.1. Synthesis of 5-Methylbenzothiazole-2-thiol (**40**)…………………………………5

S.1.3.2. Synthesis of 5-Methyl-2(3*H*)-benzothiazolone (**29**)………………………………6

S.1.4.1. Synthesis of 6-Methylbenzothiazole-2-thiol (**44**)……………………………….. 6

S.1.4.2. Synthesis of 2-Bromo-6-methylbenzothiazole (**46**)……………………………..6

S.1.4.3. Synthesis of 6-Methyl-2(3*H*)-benzothiazolone (**30**)………………………………6

S.2. Crystallography…………………………………………………………………...7

Table S 2.1. The most important crystallographic parameters for the crystal structures……7

Table S 2.2. Selected bond lengths, angles and torsion angles for the structures…………..8

Table S 2.3. Potential hydrogen bonding interactions for the structures…………………..10

Table S 2.4. Angles between normals, twist and fold angles for **25Z**, **22Z** and **27Z**……...11

Figure S 2.1. ORTEP view of the molecules in the asymmetric unit of the crystal structures of **19Z**………………………………………………………………………………………12

Figure S 2.2. ORTEP view of the molecules in the asymmetric unit of the crystal structures of **22**E……………………………..……………………………………………………12

Figure S 2.3. ORTEP view of the molecules in the asymmetric unit of the crystal structures of **26E**………………………………………………………………………………………12

Figure S 2.4. ORTEP view of the molecules in the asymmetric unit of the crystal structures of **23Z**…………………………………………………………………………………….. 13

Figure S 2.5. ORTEP view of the molecules in the asymmetric unit of the crystal structures of **24Z**………………………………………………………………………………………13

S.3. Biology…………………………………………………………………………….. 14

S.3.1. Cell lines………………………………….…………………………………………..14

Table S 3.1. Description of eukaryotic cell lines used for MTT assay……………………..14

S.3.2. Evaluation of leading benzothiazolone CA-4 analogs cytotoxic activity in different tumor and control cell lines………………………………………………………………… 15

Table S 3.2. IC_{50} values for CA-4 and **26Z** against different tumor and control cell lines following treatment for 72 hours……………………..…………...…………………….......15

Figure S 3.1. Dose-response curves of CA-4 (▲) and **26Z** (□) in various tumor cell lines…16

Figure S 3.2. Effect of **26Z** and CA-4 on the cell cycle in EA.hy926 cells …..…………… 17

S.4. NMR spectra…………………...…………..……………………………………………..18

Figure S 4.1.1. ^{1}H-NMR ($CDCl_3$): (*Z*)-4-(4-Methoxystyryl)-3-methyl-2(3*H*)-benzothiazolone (**15Z**)……..……………………………………………………………………………..18

Figure S 4.1.2. ^{13}C-NMR ($CDCl_3$): (*Z*)-4-(4-Methoxystyryl)-3-methyl-2(3*H*)-benzothiazolone (**15Z**)………………………………………………………………………………………18

Figure S 4.2.1.^{1}H-NMR ($CDCl_3$): (*Z*)-4-(3,4-Dimethoxystyryl)-3-methyl-2(3*H*)-benzothiazolone (**16Z**)……………….……………………………………………………………19

Figure S 4.2.2.^{13}C-NMR ($CDCl_3$): (*Z*)-4-(3,4-Dimethoxystyryl)-3-methyl-2(3*H*)-benzothiazolone (**16Z**)………………… …………………………………………………19

Figure S 4.3.1. ^{1}H-NMR ($CDCl_3$): (*Z*)-4-(3,5-Dimethoxystyryl)-3-methyl-2(3*H*)-benzothiazolone (**17Z**)……………………………………………………………………….20

Figure S 4.3.2. ^{13}C-NMR ($CDCl_3$): (*Z*)-4-(3,5-Dimethoxystyryl)-3-methyl-2(3*H*)-benzothiazolone (**17Z**)………………………………………………………………………20

Figure S 4.4.1. ^{1}H-NMR ($CDCl_3$): (*Z*)-3-Methyl-4-(3,4,5-trimethoxystyryl)-2(3*H*)-benzothiazolone (**18Z**)……………………… …………………………………………... 21

Figure S 4.4.2. ^{13}C-NMR ($CDCl_3$): (*Z*)-3-Methyl-4-(3,4,5-trimethoxystyryl)-2(3*H*)-benzothiazolone (**18Z**)………………………………………………………………………21

Figure S 4.5.1. ^{1}H-NMR ($CDCl_3$): (*Z*)-5-(4-Methoxystyryl)-3-methyl-2(3*H*)-benzothiazolone (**19Z**)………………………………………………………………………………………22

Figure S 4.5.2. ^{13}C-NMR ($CDCl_3$): (*Z*)-5-(4-Methoxystyryl)-3-methyl-2(3*H*)-benzothiazolone (**19Z**)………………………………………………………………………………………22

Figure S 4.6.1. ^{1}H-NMR ($CDCl_3$): (*Z*)-5-(3,4-Dimethoxystyryl)-3-methyl-2(3*H*)-benzothiazolone (**20Z**)………………………………… ………………………………23

Figure S 4.6.2. ^{13}C-NMR ($CDCl_3$): (*Z*)-5-(3,4-Dimethoxystyryl)-3-methyl-2(3*H*)-benzothiazolone (**20Z**)……………………… ……………………………………23

Figure S 4.7.1. ^{1}H-NMR ($CDCl_3$): (*Z*)-5-(3,5-Dimethoxystyryl)-3-methyl-2(3*H*)-benzothiazolone (**21Z**)……… ……………………………………………………………24

Figure S 4.7.2. ^{13}C-NMR ($CDCl_3$): (*Z*)-5-(3,5-Dimethoxystyryl)-3-methyl-2(3*H*)-benzothiazolone (**21Z**)………………………. ……………………………………..24

Figure S 4.8.1. 1H-NMR ($CDCl_3$): (*Z*)-3-Methyl-5-(3,4,5-trimethoxystyryl)-2(3*H*)-benzothiazolone (**22Z**)……………………… ………………………………………...25

Figure S 4.8.2. ^{13}C-NMR ($CDCl_3$): (*Z*)-3-Methyl-5-(3,4,5-trimethoxystyryl)-2(3*H*)-benzothiazolone (**22Z**)………..…………………………………………………25

Figure S 4.9.1. ^{1}H-NMR ($CDCl_3$): (*Z*)-6-(4-Methoxystyryl)-3-methyl-2(3*H*)-benzothiazolone (**23Z**)………………………………………………………………………………… 26

Figure S 4.9.2. ^{13}C-NMR ($CDCl_3$): (*Z*)-6-(4-Methoxystyryl)-3-methyl-2(3*H*)-benzothiazolone (**23Z**)…………………………………………………………………………………26

Figure S 4.10.1. ^{1}H-NMR ($CDCl_3$): (*Z*)-6-(3,4-Dimethoxystyryl)-3-methyl-2(3*H*)-benzothiazolone (**24Z**)……………………………………………………………………..27

Figure S 4.10.2. ^{13}C-NMR ($CDCl_3$): (*Z*)-6-(3,4-Dimethoxystyryl)-3-methyl-2(3*H*)-benzothiazolone (**24Z**)……………………………………………………………………..27

Figure S 4.11.1. ^{1}H-NMR ($CDCl_3$): (*Z*)-6-(3,5-Dimethoxystyryl)-3-methyl-2(3*H*)-benzothiazolone (**25Z**)……………………… ………………………………………………28

Figure S 4.11.2. ^{1}H-NMR (acetone-d_6): (*Z*)-6-(3,5-Dimethoxystyryl)-3-methyl-2(3*H*)-benzothiazolone (**25Z**)……………………………………………………………………28

Figure S 4.11.3. ^{13}C-NMR ($CDCl_3$): (*Z*)-6-(3,5-Dimethoxystyryl)-3-methyl-2(3*H*)-benzothiazolone (**25Z**)………………… …………………………………………………29

Figure S 4.12.1. ^{1}H-NMR ($CDCl_3$): (*Z*)-3-Methyl-6-(3,4,5-trimethoxystyryl)-2(3*H*)-benzothiazolone (**26Z**)…………… ………………………………………………………29

Figure S 4.12.2. ^{1}H-NMR (acetone-d_6): (*Z*)-3-Methyl-6-(3,4,5-trimethoxystyryl)-2(3*H*)-benzothiazolone (**26Z**)……………………………………………………………………30

Figure S 4.12.3. ^{13}C-NMR ($CDCl_3$): (*Z*)-3-Methyl-6-(3,4,5-trimethoxystyryl)-2(3*H*)-benzothiazolone (**26Z**)……………………………………………………………………30

Figure S 4.13.1. ^{1}H-NMR ($CDCl_3$): (*E*)-3-Methyl-6-(3,4,5-trimethoxystyryl)-2(3*H*)-benzothiazolone (**26E**)………………………… ……………………………………31

Figure S 4.13.2. ^{1}H-NMR (acetone-d6): (*E*)-3-Methyl-6-(3,4,5-trimethoxystyryl)-2(3*H*)-benzothiazolone (**26E**)……………………………………………………………………31

Figure S 4.14.1. ^{1}H-NMR ($CDCl_3$): (*Z*)-3-Methyl-7-(3,4,5-trimethoxystyryl)-2(3*H*)-benzothiazolone (**27Z**)……………………………………………………………………32

Figure S 4.14.2. ^{13}C-NMR ($CDCl_3$): (*Z*)-3-Methyl-7-(3,4,5-trimethoxystyryl)-2(3*H*)-benzothiazolone (**27Z**)……………………………………………………………………32

References ………………………..…………………………………………………………. .33

S1. Chemistry

S.1.1. General information

Melting points (m.p.) were determined on a Boetius hot-stage microscope. ^{1}H and ^{13}C NMR spectra were obtained with a Bruker DRX250, Bruker DRX400, and DRX 500 spectrometer in $CDCl_3$ or acetone-d_6 as solvent. Chemical shifts were reported in parts per million (ppm, δ) relative to the solvent peak (7.26 ppm for ^{1}H; 77.16 ppm for ^{13}C). Coupling constants (J) were measured in Hertz (Hz). Elemental analyses (C, H, N) were carried out by a Vario III microanalyzer. Obtained results were within 0.4% of theoretical values. Thin-layer chromatography (TLC) was carried out on silica gel plates (Kieselgel 60 F_{254}). Flash column chromatography was performed with Merck 60 silica gel (0.040-0.063 mm).

S.1.2. Synthesis of 4-methyl-2(3*H*)-benzothiazolone (**28**)

In 50 ml methoxyethanol were added 10.26 g (0.045 mol) 2-bromo-4-methylbenzothiazole (**39**) [1] and 10 ml conc. HCl. The reaction mixture was heated under reflux for 4 h until complete by TLC and poured into 100 ml cold water. The formed precipitate was filtered and washed with water yielding 6.30g (85%) of crude **28**. M.p. 204-205 °C (ethanol), lit. m.p. 210 - 212 °C [2].

S.1.3.1. Synthesis of 5-Methylbenzothiazole-2-thiol (**40**)

Sodium polysulfide solution was prepared by dissolving 19.2 g (0.06 mol) sulfur in a hot solution of 72.1 g (0.03 mol) $Na_2S \times 9H_2O$ in 60 ml water. Heat was applied until the full dissolution of the sulfur. After cooling to r.t. the polysulfide solution was transferred to a 250 round bottom flask equipped with a long reflux condenser and to it was added 17.1 g (0.1 mol) 4-chloro-3-nitrotoluene. 12.1 g (0.2 mol) carbon disulfide was added with vigorous stirring with a magnetic stir bar and the mixture was heated under reflux for 5 h. The excess CS_2 was distilled off, the remaining mixture was diluted with water to 300 ml, and acidified with 1 volume dil. HCl. The precipitated mixture of 5-methylbenzothiazole-2-thiol (**40**) and sulfur was filtered, and washed with water. The product was separated from the sulfur by dissolving in 300 ml water and 30 ml 25 % ammonium hydroxide at 80°. The sulfur is filtered off and the filtrate is acidified with 10% HCl to precipitate 5-methylbenzothiazole-2-thiol (**40**). The product is filtered, washed with water and dried at 50 °C to yield 8.52 g (47%). M.p. 173-175 °C (ethanol), lit. m.p. 171-173 [3].

S.1.3.2. Synthesis of 5-Methyl-2(3*H*)-benzothiazolone (**29**)

5.44 g (0.03 mol) 5-methylbenzothiazole-2-thiol (**40**) was added to a solution of 2.37 g (0.036 mol) potassium hydroxide in 20 ml water. The suspension was stirred until full dissolution. The clear solution was diluted to 100 ml with water and drop-wise with vigorous stirring was added a solution of 11 g (0.07 mol) $KMnO_4$ in 120 ml water. The reaction was judged complete when the color of a drop of solution in filter paper was pale pink, indicating small excess of permanganate. The precipitated MnO_2 was filtered off and washed several times with water. The filtrate was acidified with conc. HCl to pH 1 and boiled until the emission of SO_2 had stopped. After cooling, the precipitated 5-methyl-2(3*H*)-benzothiazolone (**29**) was filtered and washed with water. Yield 4.36 g (88%). M.p. 179-181 °C (50% ethanol), lit. m.p. 182-184 [4].

S.1.4.1. Synthesis of 6-Methylbenzothiazole-2-thiol (**44**)

A mixture of 18.6 g (0.1 mol) 2-bromo-4-methylaniline и 32.1 g (0.2 mol) potassium ethyl xanthogenate in 80 ml *N*-methyl-2-pyrrolidone (NMP) was heated to 130 °C for 2 h (TLC). After cooling to room temperature the reaction mixture was poured in 200 ml water and acidified with 10% HCl. The forming precipitate was filtered, washed with water, and dried at 50 ºC. Yield 14.7 g (81%). M.p. 179-181 °C (50% ethanol), lit. m.p. 180-181 °C [5].

S.1.4.2. Synthesis of 2-Bromo-6-methylbenzothiazole (**46**).

To a mixture of 80 mL dichloromethane and 15 mL acetic acid, 9.85 g (0.06 mol) 2-amino-6-methylbenzothiazole (**45**) [7], was added, followed by 35 mL 48% HBr. After cooling of the resulting suspension on an ice bath, a solution of 10 g (0.14 mol) $NaNO_2$ in 20 mL water was added slowly keeping the temperature at 5 °C. After the addition was complete, the reaction mixture was stirred at room temperature followed by heating at 40 °C for 15 min (nitrogen was released). Then, the obtained dark mixture was cooled and washed successively with 2 × 50 mL water, 2 × 50 mL 5% aq $NaHSO_3$, and water again. The organic layer was separated, dried over Na_2SO_4, filtered and concentrated in vacuo to afford **46** as yellow oil in 86% yield. The oil was dissolved in petroleum ether (5 mL) and allowed to crystallize slowly at 4 °C. Yield: 10.3 g (75%), M.p. 48-50 °C, lit. m.p. 47 ºC [6], lit. m.p. 54-56 ºC [7].

S.1.4.3. Synthesis of 6-Methyl-2(3*H*)-benzothiazolone (**30**)

The compound was obtained from 6-methylbenzothiazole-2-thiol (**44**) following procedure 1.3.2. with a yield of 91%. Alternatively the compound was obtained from 2-bromo-6-methylbenzothiazole (**46**) following procedure 1.2. with a yield of 89%. M.p. 167-169 °C (50% ethanol), lit. m.p. 168-169 °C [8].

S.2. Crystallography

Table S 2.1. The most important crystallographic parameters for the crystal structures

Structure code	**24Z**	**27Z**	**23Z**	**25Z**	**19Z**
Empirical formula	$C_{18}H_{17}NO_3S$	$C_{19}H_{19}NO_4S$	$C_{17}H_{15}NO_2S$	$C_{18}H_{17}NO_3S$	$C_{17}H_{15}NO_2S$
Formula weight	327.38	357.41	297.36	327.38	297.36
Temperature/K	290	290	290.0	290	290.0
Crystal system	Monoclinic	Monoclinic	Orthorhombic	Orthorhombic	Orthorhombic
Space group	$P2_1/n$	$C2/c$	$P2_12_12_1$	$Pca2_1$	$Pca2_1$
a/Å	8.5946(5)	28.942(4)	7.6444(3)	7.5669(2)	15.7095(8)
b/Å	22.6744(11)	7.8598(5)	13.0845(5)	10.1175(4)	6.7976(3)
c/Å	9.1614(5)	20.296(2)	14.7030(4)	21.7151(8)	13.8849(5)
α/°	90	90	90.0	90	90.0
β/°	111.379(6)	131.35(2)	90.0	90	90.0
γ/°	90	90	90.0	90	90.0
Volume/Å^3	1662.50(17)	3465.8(10)	1470.64(9)	1662.48(10)	1482.72(12)
Z	4	8	4	4	4
ρ_{calc}g/cm^3	1.308	1.370	1.343	1.308	1.332
μ/mm^{-1}	0.209	0.211	0.000	0.209	0.000
F(000)	688.0	1504.0	624.0	688.0	624.0
Crystal size/mm^3	0.3 × 0.25 × 0.12	0.3 × 0.25 × 0.12	0.3 × 0.25 × 0.12	0.3 × 0.25 × 0.12	0.3 × 0.25 × 0.12
Radiation	Mo$K\alpha$ λ = 0.71073	Mo$K\alpha$ λ = 0.71073	Mo$K\alpha$ λ = 0.71073	Mo$K\alpha$ λ = 0.71073	Mo$K\alpha$ λ = 0.71073
2Θ range for data collection/°	5.85 to 58.984	5.644 to 58.104	6.006 to 58.974	6.724 to 59.382	5.868 to 59.098
Index ranges	-11 ≤ h ≤ 10, -28 ≤ k ≤ 17, -11 ≤ l ≤ 12	-36 ≤ h ≤ 39, -10 ≤ k ≤ 9, -25 ≤ l ≤ 27	-10 ≤ h ≤ 7, -13 ≤ k ≤ 18, -15 ≤ l ≤ 20	-10 ≤ h ≤ 7, -12 ≤ k ≤ 12, -26 ≤ l ≤ 29	-21 ≤ h ≤ 15, -9 ≤ k ≤ 7, -17 ≤ l ≤ 18
Reflections collected/independent	9355/3964	12324/3986	7037/3287	6280/2979	6257/3097
R_{int}/R_{sigma}	0.0236/0.0324	0.0531/0.0648	0.0260/0.0356	0.0233/0.0302	0.0296/0.0349
Data/restraints/parameters	3964/0/211	3986/0/230	3287/0/192	2979/1/211	3097/1/192
Goodness-of-fit on F^2	1.023	1.027	1.078	1.088	1.083
Final R indexes [I>=2σ (I)]	R_1 = 0.0472 wR_2 = 0.1052	R_1 = 0.0864 wR_2= 0.2132	R_1 = 0.0401 wR_2 = 0.0799	R_1 = 0.0383 wR_2 = 0.0883	R_1 = 0.0462 wR_2 = 0.0986
Final R indexes [all data]	R_1 = 0.0812 wR_2 = 0.1259	R_1 = 0.1640 wR_2 = 0.2600	R_1 = 0.0556 wR_2 = 0.0880	R_1 = 0.0511 wR_2 = 0.0976	R_1 = 0.0727 wR_2 = 0.1098
Largest diff. peak/hole / e Å^{-3}	0.18/-0.28	0.32/-0.25	0.20/-0.15	0.14/-0.19	0.14/-0.17

Structure code	**26E**	**22Z**	**22E**
Empirical formula	$C_{19}H_{19}NO_4S$	$C_{19}H_{19}NO_4S$	$C_{19}H_{19}NO_4S$
Formula weight	357.41	357.41	357.41
Temperature/K	290	290	290
Crystal system	Monoclinic	Orthorhombic	Monoclinic
Space group	$P2_1/c$	$Pcab$	$P2_1/n$
a/Å	10.998(4)	12.383(4)	11.4641(7)
b/Å	14.232(6)	15.610(7)	11.7561(3)
c/Å	11.472(5)	18.324(5)	13.9331(4)
α/°	90	90	90
β/°	104.55(2)	90	109.057(10)

γ/°	90	90	90
Volume/Å^3	1738.1(12)	3542(2)	1774.89(16)
Z	4	8	4
ρ_{calc}g/cm^3	1.366	1.341	1.338
μ/mm^{-1}	0.210	0.206	0.206
F(000)	752.0	1504.0	752.0
Crystal size/mm^3	0.26 × 0.26 × 0.2	0.3 × 0.3 × 0.3	0.32 × 0.3 × 0.3
Radiation	Mo$K\alpha$ λ = 0.71073	Mo$K\alpha$ λ = 0.71073	Mo$K\alpha$ λ = 0.71073
2Θ range for data collection/°	3.826 to 51.928	4.446 to 51.942	4.012 to 55.942
Index ranges	0 ≤ h ≤ 13, -17 ≤ k ≤ 17, -14 ≤ l ≤ 5	0 ≤ h ≤ 15, -19 ≤ k ≤ 19, -22 ≤ l ≤ 22	0 ≤ h ≤ 15, -15 ≤ k ≤ 15, -18 ≤ l ≤ 17
Reflections collected/independent	5637/2837	13009/3474	8396/4251
R_{int}/R_{sigma}	0.0825/0.1079	0.2737/0.1838	0.0822/0.1143
Data/restraints/parameters	2837/0/230	3474/0/230	4251/0/230
Goodness-of-fit on F^2	0.967	0.946	0.947
Final R indexes [I>=2σ (I)]	R_1 = 0.0546 wR_2 = 0.1110	R_1 = 0.0773 wR_2 = 0.1454	R_1 = 0.0526 wR_2 = 0.1011
Final R indexes [all data]	R_1 = 0.1370 wR_2 = 0.1380	R_1 = 0.2312 wR_2 = 0.2001	R_1 = 0.1822, wR_2 = 0.1357
Largest diff. peak/hole / e Å^{-3}	0.17/-0.21	0.27/-0.25	0.14/-0.18

Table S 2.2. Selected bond lengths, angles and torsion angles for the structures

Structure	19Z	25Z	23Z	27Z	24Z
**Bonds**	**Å**	**Å**	**Å**	**Å**	**Å**
S1—C4	1.747 (4)	1.744 (3)	1.740 (3)	1.761 (4)	1.746 (2)
S1—C1	1.777 (5)	1.781 (4)	1.783 (3)	1.777 (5)	1.778 (3)
O1—C1	1.223 (5)	1.209 (4)	1.214 (3)	1.218 (6)	1.217 (3)
N1—C1	1.350 (5)	1.366 (4)	1.367 (4)	1.377 (7)	1.365 (3)
N1—C2	1.460 (5)	1.449 (4)	1.454 (4)	1.452 (6)	1.455 (3)
N1—C3	1.397 (5)	1.386 (4)	1.393 (3)	1.400 (6)	1.387 (3)
C7—C9	1.484 (5)	–	–	–	–
C6—C9	–	1.461 (5)	1.470 (4)	–	1.463 (4)
C5—C9	–	–	–	1.464 (6)	–
C10—C9	1.333 (5)	1.332 (5)	1.331 (4)	1.326 (6)	1.324 (4)
C10—C11	1.474 (6)	1.474 (5)	1.477 (4)	1.473 (6)	1.484 (3)
O13—C13	–	1.369 (4)	–	1.369 (5)	1.369 (2)
O14—C14	1.367 (5)	**–**	1.366 (3)	1.375 (5)	1.369 (2)
O15—C15	–	1.374 (4)	–	1.359 (6)	–
O13—C13A	–	1.426 (4)	–	1.412 (6)	1.411 (3)
O14—C14A	1.414 (5)	**–**	1.431 (4)	1.434 (6)	1.402 (3)
O15—C15A	–	1.417 (5)	–	1.412 (6)	–
**Angles**	**°**	**°**	**°**	**°**	**°**
C4—S1—C1	90.9 (2)	91.6 (2)	91.7 (1)	90.9 (2)	91.3 (1)
C3—C4—S1	111.2 (3)	110.3 (3)	110.6 (2)	111.2 (3)	110.5 (2)
C5—C4—S1	128.2 (3)	129.1 (2)	128.4 (2)	125.2 (3)	127.7 (2)
N1—C1—S1	109.9 (3)	109.4 (3)	109.2 (2)	109.6 (3)	109.6 (2)
O1—C1—S1	123.6 (4)	124.7 (3)	123.9 (3)	124.9 (5)	124.4 (2)
O1—C1—N1	126.5 (4)	126.0 (4)	126.9 (3)	125.5 (5)	126.0 (2)

C4—C3—N1	112.3 (3)	113.2 (3)	112.8 (2)	113.6 (4)	113.5 (2)
C1—N1—C2	120.9 (3)	120.7 (3)	120.8 (3)	120.9 (4)	121.3 (2)
C1—N1—C3	115.7 (3)	115.5 (3)	115.6 (2)	114.7 (4)	115.1 (2)
C8—C3—N1	127.3 (3)	126.8 (3)	127.1 (3)	127.2 (4)	127.0 (2)
C13—O13—C13A	–	117.2 (3)	–	117.4 (4)	117.2 (2)
C14—O14—C14A	118.3 (4)	–	117.5 (2)	112.9 (4)	117.6 (2)
C15—O15—C15A	–	118.6 (3)	–	118.2 (4)	–
**Torsion angles**	°	°	°	°	°
C2—N1—C1—O1	−2.7 (6)	1.2 (5)	2.9 (5)	−0.9 (9)	0.9 (3)
C2—N1—C3—C8	1.6 (6)	−1.8 (4)	−1.6 (5)	2.5 (8)	2.2 (3)
C7—C8—C3—N1	178.0 (4)	−178.8 (3)	178.2 (3)	−177.1 (5)	179.64 (18)
S1—C4—C5—C6	−179.9 (3)	177.2 (2)	179.9 (2)	−179.0 (4)	−178.57 (14)
C11—C10—C9—C7	−8.4 (7)	–	–	–	–
C11—C10—C9—C6	–	−7.0 (7)	6.6 (7)	–	6.3 (5)
C11—C10—C9—C5	–	–	–	5.5 (10)	–
C12—C13—O13—C13A	–	−0.2 (5)	–	3.7 (7)	−8.3 (3)
C15—C14—O14—C14A	−0.8 (6)	–	1.7 (4)	102.3 (5)	18.8 (3)
C16—C15—O15—C15A	–	178.4 (4)	–	−3.5 (7)	–
O13—C13—C14—O14	–	–	–	1.4 (6)	−2.2 (2)
O14—C14—C15—O15	–	–	–	−2.5 (6)	–

**Structure**	**22Z**	**26E**	**22E**
**Bonds**	**Å**	**Å**	**Å**
S1—C4	1.745 (5)	1.742 (3)	1.740 (3)
S1—C1	1.796 (6)	1.782 (4)	1.774 (3)
O1—C1	1.211 (6)	1.207 (4)	1.216 (4)
N1—C1	1.359 (7)	1.359 (5)	1.364 (4)
N1—C2	1.441 (6)	1.469 (4)	1.455 (3)
N1—C3	1.400 (6)	1.390 (4)	1.393 (4)
C7—C9	1.489 (7)	–	1.467 (4)
C6—C9	–	1.469 (5)	–
C5—C9	–	–	–
C10—C9	1.333 (7)	1.314 (5)	1.309 (4)
C10—C11	1.459 (7)	1.471 (5)	1.469 (4)
O13—C13	1.371 (6)	1.356 (4)	1.364 (3)
O14—C14	1.372 (6)	1.382 (4)	1.376 (3)
O15—C15	1.373 (6)	1.360 (4)	1.362 (3)
O13—C13A	1.423 (7)	1.417 (4)	1.420 (3)
O14—C14A	1.413 (6)	1.425 (5)	1.421 (4)
O15—C15A	1.427 (7)	1.434 (4)	1.415 (4)
**Angles**	°	°	°
C4—S1—C1	90.8 (3)	92.0 (2)	91.5 (2)
C3—C4—S1	111.8 (4)	110.0 (2)	111.2 (2)
C5—C4—S1	128.6 (5)	128.7 (3)	129.3 (2)
N1—C1—S1	109.1 (5)	108.9 (3)	109.2 (2)
O1—C1—S1	123.2 (5)	124.4 (3)	124.7 (3)
O1—C1—N1	127.6 (6)	126.7 (4)	126.1 (3)
C4—C3—N1	112.3 (5)	113.4 (3)	112.2 (3)
C1—N1—C2	120.1 (5)	120.6 (3)	120.7 (3)
C1—N1—C3	116.0 (5)	115.8 (3)	115.9 (3)
C8—C3—N1	126.2 (5)	127.1 (3)	126.3 (3)
C13—O13—C13A	117.7 (5)	117.5 (3)	117.6 (2)
C14—O14—C14A	114.0 (5)	113.1 (3)	113.7 (2)
C15—O15—C15A	116.9 (5)	118.2 (3)	118.2 (3)
**Torsion angles**	°	°	
C2—N1—C1—O1	-2.1 (9)	-0.5 (7)	-2.8 (5)
C2—N1—C3—C8	5.7 (8)	-0.9 (7)	2.6 (5)
C7—C8—C3—N1	-179.9 (5)	-178.5 (4)	-178.8 (3)
S1—C4—C5—C6	-179.1 (4)	180.0 (3)	-178.9 (3)

C11—C10—C9—C7	10.1 (1)	–	–
C11—C10—C9—C6	–	178.7 (4)	-178.9 (3)
C11—C10—C9—C5	–	–	–
C12—C13—O13—C13A	11.6 (8)	-3.6 (6)	0.6 (4)
C15—C14—O14—C14A	-102.6 (6)	94.0 (4)	86.7 (4)
C16—C15—O15—C15A	-9.7 (8)	6.7 (6)	9.3 (5)
O13—C13—C14—O14	-3.8 (7)	4.4 (6)	2.0 (4)
O14—C14—C15—O15	2.1 (7)	-3.3 (5)	-2.2 (4)

Table S 2.3. Potential hydrogen bonding interactions for the structures

**Structure 24Z**				
D—H···A	***D—H*** **(Å)**	***H···A*** **(Å)**	***D···A*** **(Å)**	***D—H···A*** **(°)**
C8—H8···O14^{i}	0.93	2.64	3.560 (3)	172
C2—H2C···O1^{i}	0.96	2.58	3.488 (3)	159
C14A—H14C···O1^{ii}	0.96	2.49	3.447 (3)	173
Symmetry operations: *(i) −x+2, −y, −z+2; (ii) −x+3/2, y+1/2, −z+3/2.*				
**Structure 27Z**				
C14A—H14B···O1^{i}	0.96	2.54	3.494 (6)	171
C14A—H14C···O13	0.96	2.56	3.076 (6)	114
Symmetry operation: *(i) x+1/2, y+1/2, z+1.*				
**Structure 23Z**				
C14A—H14A···O1^{i}	0.96	2.58	3.402 (4)	144
Symmetry operation: *(i) x, y−1, z.*				
**Structure 25Z**				
C14—H14···O1^{i}	0.93	2.62	3.496 (4)	157
C8—H8···O15^{ii}	0.93	2.48	3.282 (4)	145
Symmetry operations: *(i) −x, −y, z+1/2; (ii) x+1/2, −y, z.*				
**Structure 19Z**				
C15—H15···O1^{i}	0.93	2.44	3.337 (4)	162
C2—H2C···S1^{ii}	0.96	2.87	3.770 (5)	156
Symmetry operations*: (i) x, y, z+1; (ii) x, y+1, z.*				
**Structure 22Z**				
C2—H2A···O14^{i}	0.96	2.52	3.240 (7)	132
Symmetry operation*: (i) x+1/2, −y+3/2, z.*				
**Structure 26E**				

C2—H2C···O15[i]	0.96	2.61	3.374 (5)	137
C15A—H15C···O13[ii]	0.96	2.64	3.460 (5)	143
C14A—H14C···O1[iii]	0.96	2.63	3.562 (5)	164
C13A—H13B···O15[iv]	0.96	2.57	3.419 (5)	147
***Symmetry operations**: (i) −x+1, −y+1, −z+1; (ii) −x, y+1/2, −z+3/2; (iii) x−1, y, z+1; (iv) −x, y−1/2, −z+3/2.*				
**Structure 22E**				
C5—H5···O1[i]	0.93	2.52	3.359 (4)	150

***Symmetry operation**: (i) −x+3/2, y−1/2, −z+1/2.*

Table S 2.4. Angles between normals, twist and fold angles for 25Z, 22Z and 27Z

Planes / Angle type [°]	Metoxybenzene / double bond	Benzotiazole / double bond
	**25Z**	
Angles between normals	140.55	46.45
Twist angles	140.91	44.50
Fold angles	30.4	15.01
	**22Z**	
Angle between normals	33.0	55.8
Twist angles	32.7	54.7
Fold angles	5.7	13.1
	**27Z**	
Angle between normals	138.6	27.1
Twist angles	139.2	25.5
Fold angles	28.6	9.48

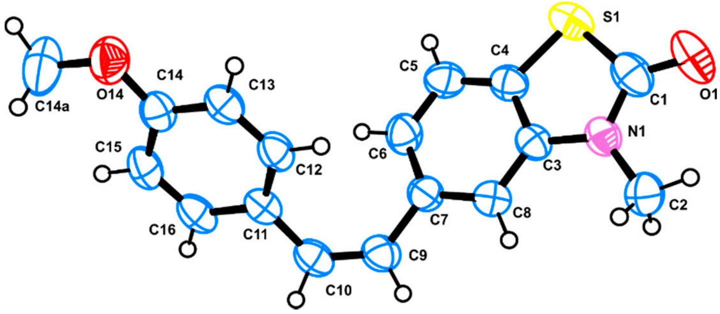

Figure S 2.1. ORTEP view of the molecules in the asymmetric unit of the crystal structures of **19Z**

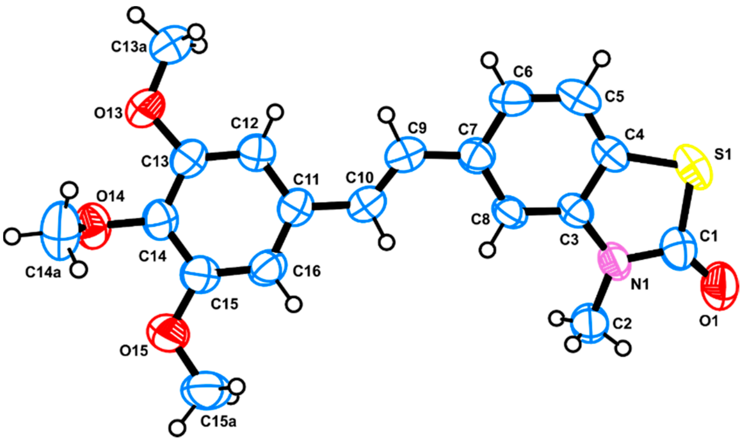

Figure S 2.2. ORTEP view of the molecules in the asymmetric unit of the crystal structures of **22E**

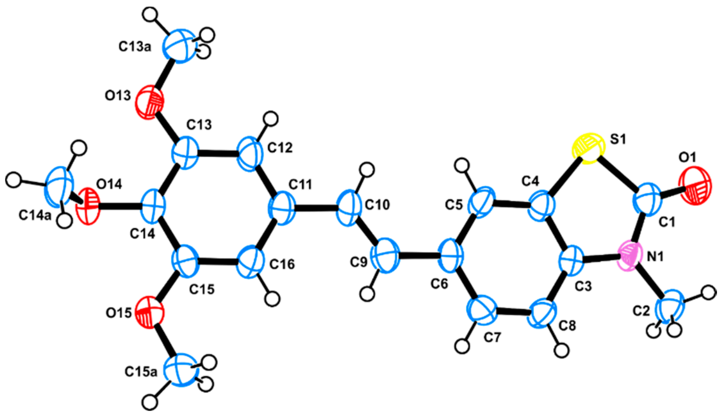

Figure S 2.3. ORTEP view of the molecules in the asymmetric unit of the crystal structures of **26E**

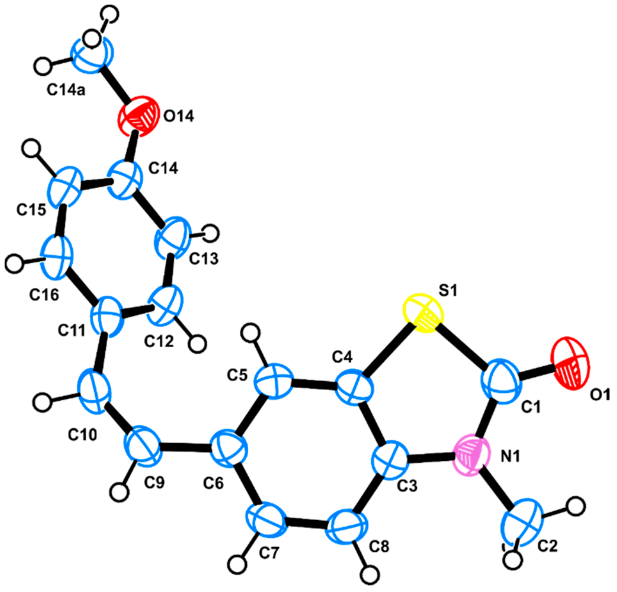

Figure S 2.4. ORTEP view of the molecules in the asymmetric unit of the crystal structures of **23Z**

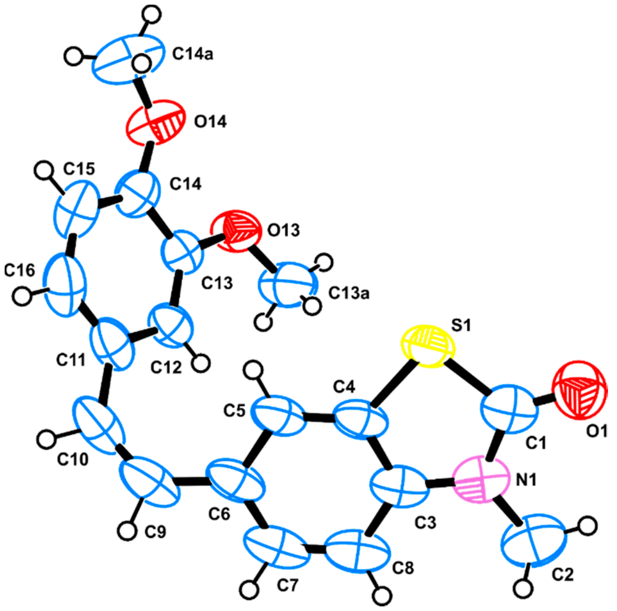

Figure S 2.5. ORTEP view of the molecules in the asymmetric unit of the crystal structures of **24Z**

S.3. Biology

S.3.1. Cell lines

The cell lines used and the conditions of growth are shown in Table S 3.1.

Table S 3.1. Description of eukaryotic cell lines used for MTT assay.

Panel	Cell line	Origin	Tissue, characteristics	Source	Culture conditions
Lung	**A549**	Human (h)	lung carcinoma	ATCC, CCL-185	3.1 g/L D-Glucose DMEM/F12 with 10% (v/v) FBS, 100 U/mL penicillin, 100 U/mL streptomycin (Lonza)
	**BEAS-2B**	h	lung, bronchus; epithelial virus transformed	ATCC, CRL-9609	BEBM medium supplemented with insulin, rhEGF, GA-1000, BPE, transferrin, hydrocortisone, triiodothyronine (T3), epinephrine, retinoic acid (Lonza)
Colon	**HT-29**	h	colon, colorectal adenocarcinoma	Kindly provided by Prof. Radostina Alexandrova (IEMPAM-BAS)	3.1 g/L D-Glucose DMEM/F12 with 10% (v/v) FBS, 100 U/mL penicillin, 100 U/mL streptomycin (Lonza)
	**Colon-26**	mouse	colon, colon carcinoma, grade IV, undifferentiated	Kindly provided by Prof. Iana Tsoneva (IBPhBME – BAS)	
Breast	**MCF-7**	h	mammary gland, breast; derived from metastatic site: pleural effusion; adenocarcinoma (ER+)	Kindly provided by Prof. Iana Tsoneva (IBPhBME – BAS)	3.1 g/L Glucose, DMEM/F12 with 10% FBS, 100 U/mL penicillin, 100 U/mL streptomycin (Lonza).
	**MDA-MB-231**	h	mammary gland/breast; derived from metastatic site: pleural effusion; adenocarcinoma (ER-)		
	**MCF-10A**	h	mammary gland; breast; fibrocystic disease; non-		

			tumorigenic epithelial cell line		
**Endothelial**	**EA hy.926**	h	endothelial; primary human umbilical vein cells fused with a thioguanine-resistant clone of A549.	Kindly provided by Dr. C-J.S. Edgell - University of North Carolina, USA	4.5 g/L D-Glucose, DMEM/F12 with 10% FBS, 100 U/mL penicillin, 100 U/mL streptomycin (Gibco)

S.3.2. Evaluation of leading benzothiazolone CA-4 analogs cytotoxic activity in different tumor and control cell lines

Table S 3.2. IC_{50} values for CA-4 and 26Z against different tumor and control cell lines following treatment for 72 hours

**Cell line**	**IC_{50} (μM)***	
	**CA-4**	**26Z**
**MCF-7**	Cytostatic (40% viable)	2.42 ± 0.48
**MDA-MB-231**	Cytostatic (55% viable)	1.35 ± 0.42
**MCF-10A**	1.64 ± 0.34	Cytostatic (65% viable)
**Colon-26**	0.66 ± 0.08	Cytostatic (30% viable)
**HT-29**	2.16 ± 0.23	0.008 ± 0.001
**A549**	3.01 ± 0.16	Cytostatic (40% viable)
**BEAS-2B**	Cytostatic (80% viable)	Cytostatic (80% viable)

*Results are given as IC_{50} ± SE, n=3 independent experiments in eight replicates each.

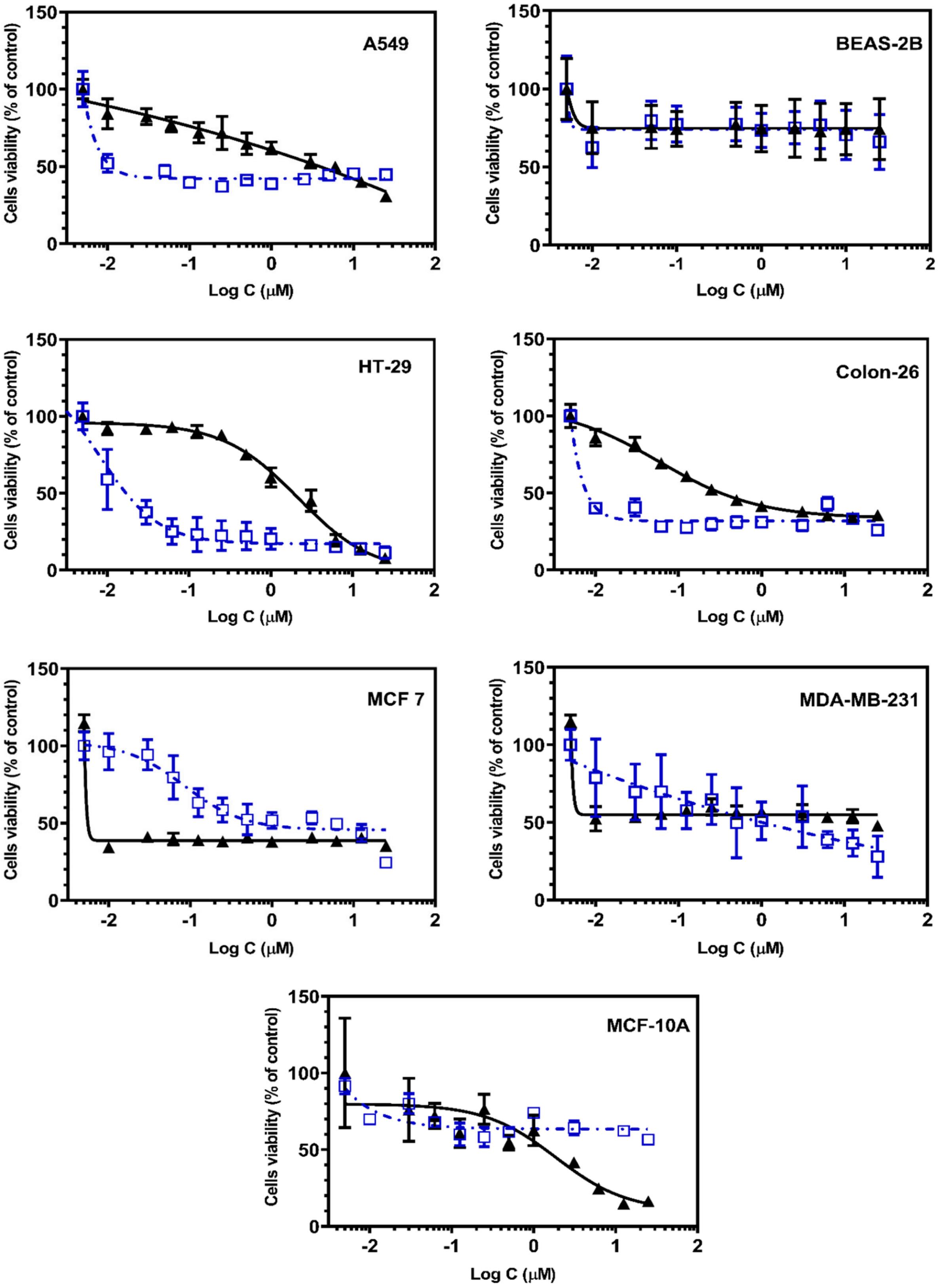

Figure S 3.1. Dose-response curves of CA-4 (▲) and **26Z** (□) in various tumor cell lines. Cells were treated with varying concentrations of drug [0.0025–25 μM]. After 72 h, cell viability was assessed using the MTT assay. Data is expressed as percentage viability of vehicle treated

controls. Values represent the means ± SE for three separate experiments carried out in eightplicate.

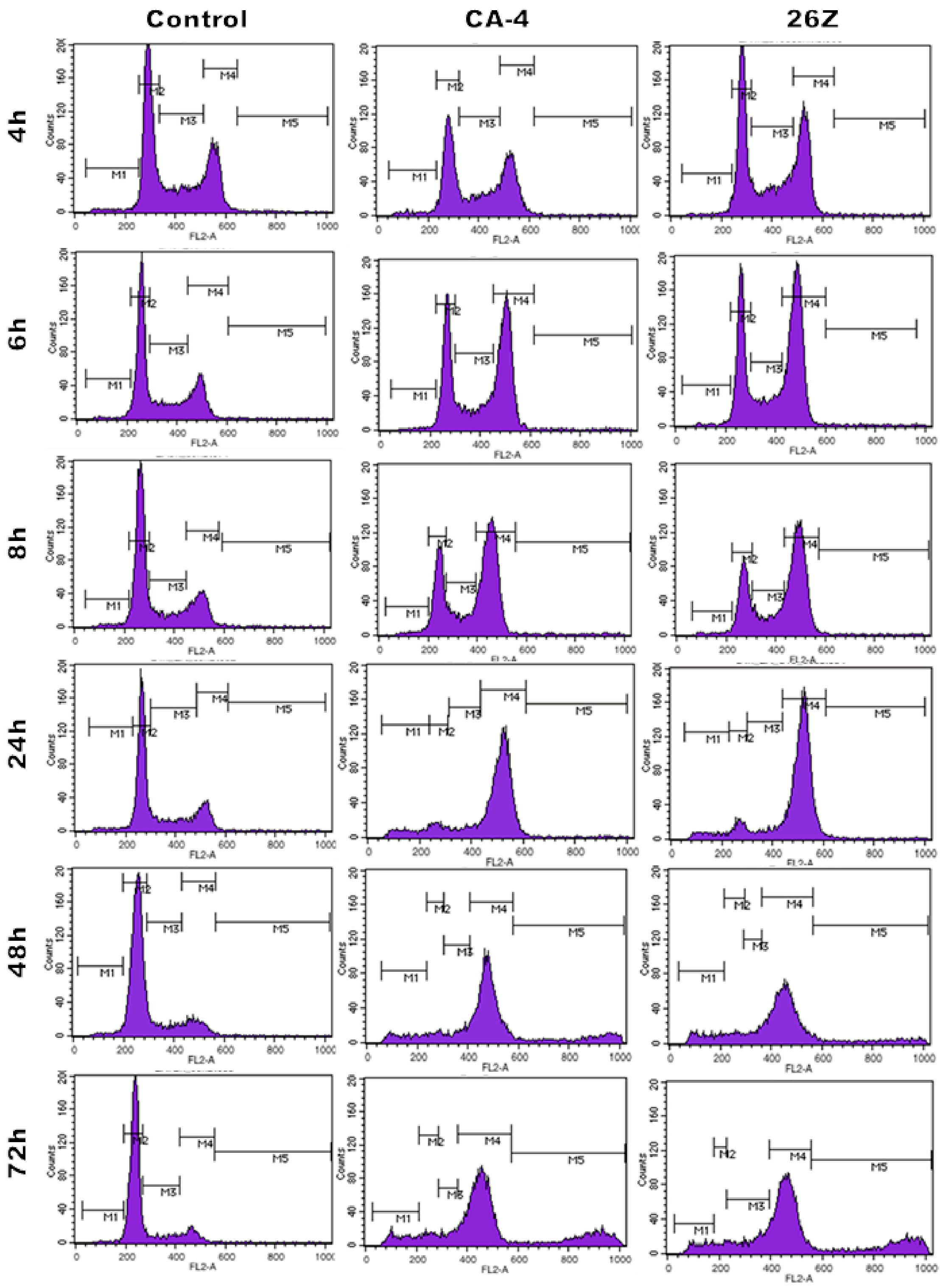

Figure S 3.2. Effect of **26Z** and CA-4 on the cell cycle in EA.hy926 cells. The cells were treated with 10 nM CA-4 or 300 nM **26Z** for different time, stained with propidium iodide for DNA, and analysed by flow cytometry. The representative histograms of the original data are shown. Markers (M1: Sub-G1, M2: G0/G1, M3: S, M4: G2/M, M5: polyploidy) different cell cycle phases.

S.4. NMR spectra

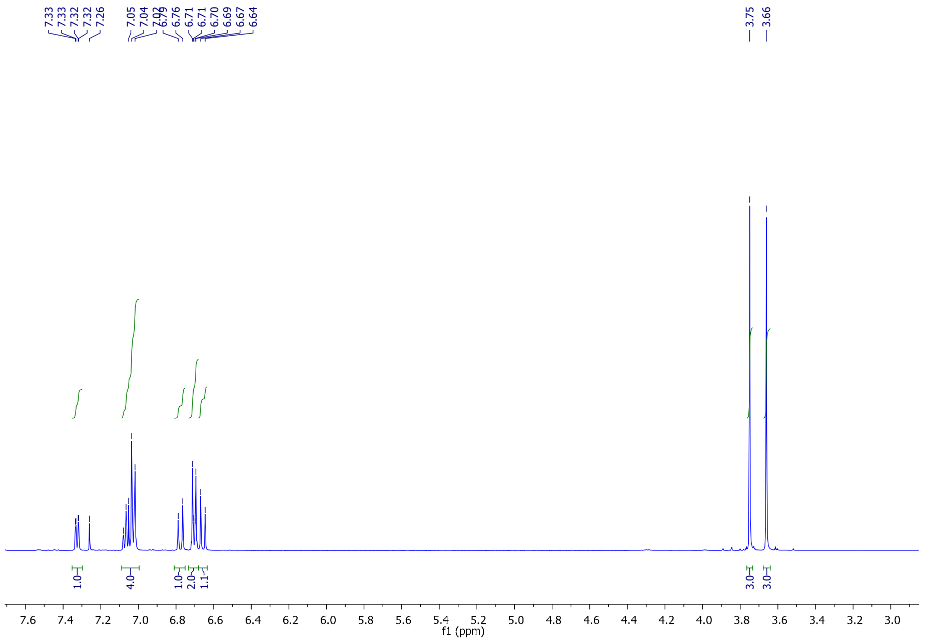

Figure S 4.1.1. ^{1}H-NMR ($CDCl_3$): (*Z*)-4-(4-Methoxystyryl)-3-methyl-2(3*H*)-benzothiazolone (**15Z**)

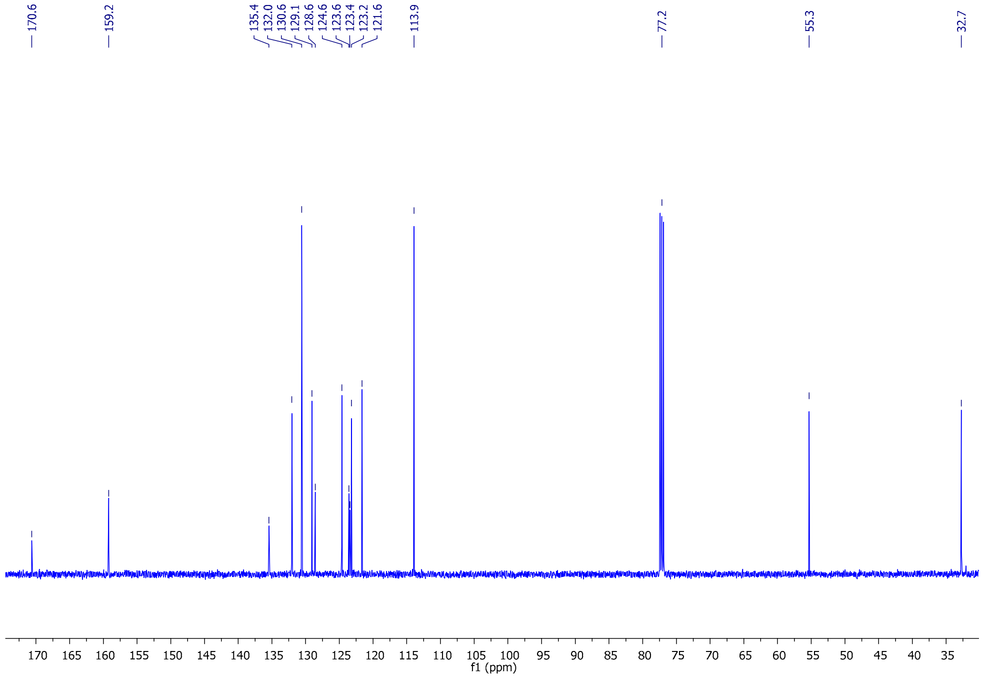

Figure S 4.1.2. ^{13}C-NMR ($CDCl_3$): (*Z*)-4-(4-Methoxystyryl)-3-methyl-2(3*H*)-benzothiazolone (**15Z**)

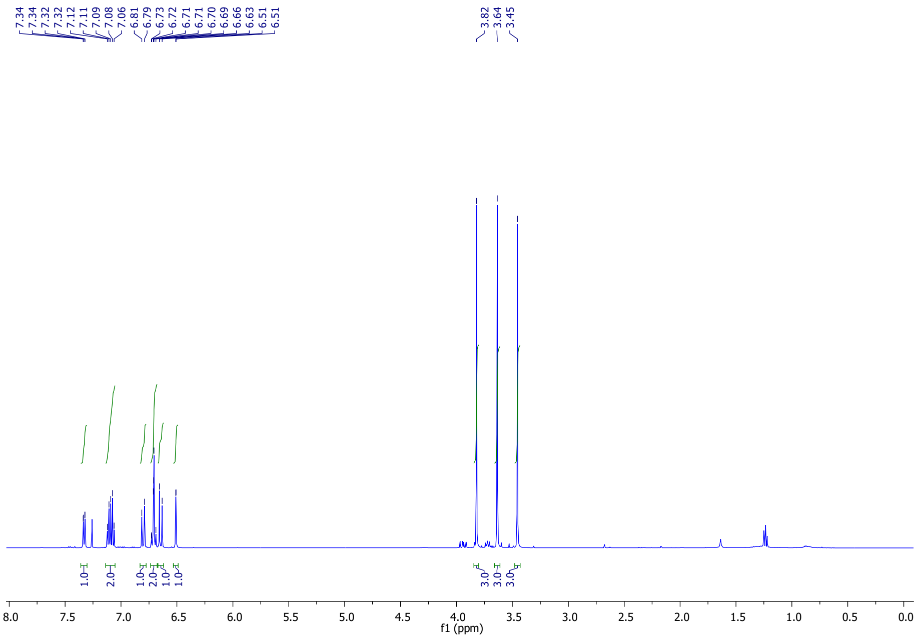

Figure S 4.2.1.^{1}H-NMR (CDCl3): (*Z*)-4-(3,4-Dimethoxystyryl)-3-methyl-2(3*H*)-benzothiazolone (**16Z**)

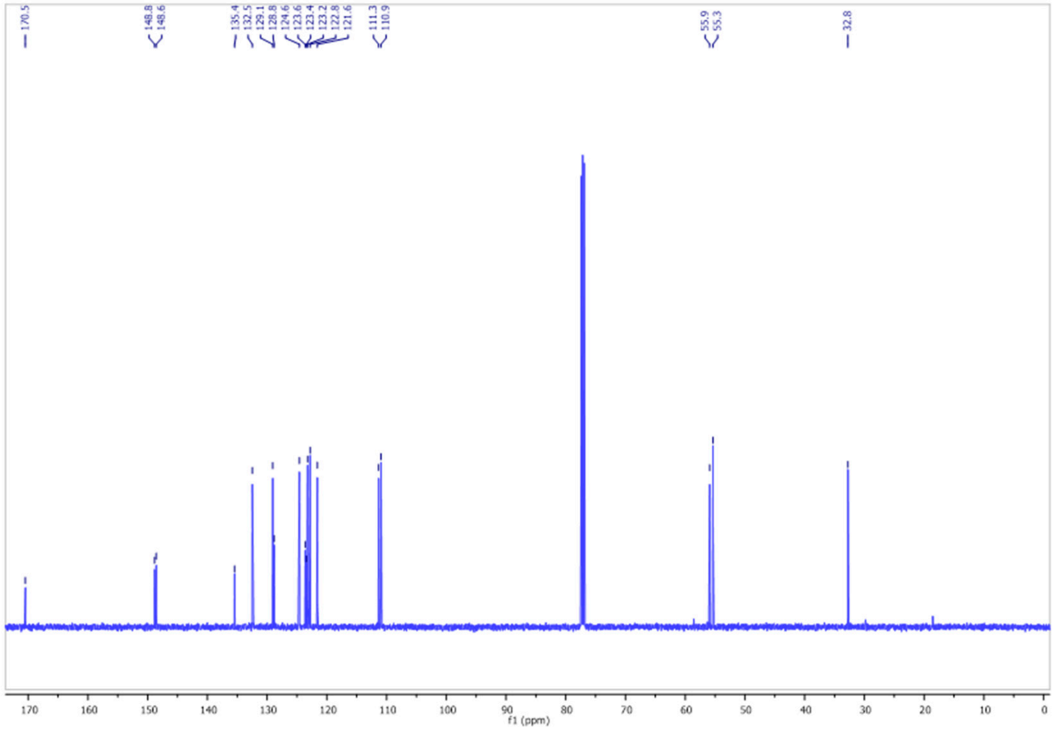

Figure S 4.2.2.^{13}C-NMR (CDCl3): (*Z*)-4-(3,4-Dimethoxystyryl)-3-methyl-2(3*H*)-benzothiazolone (**16Z**)

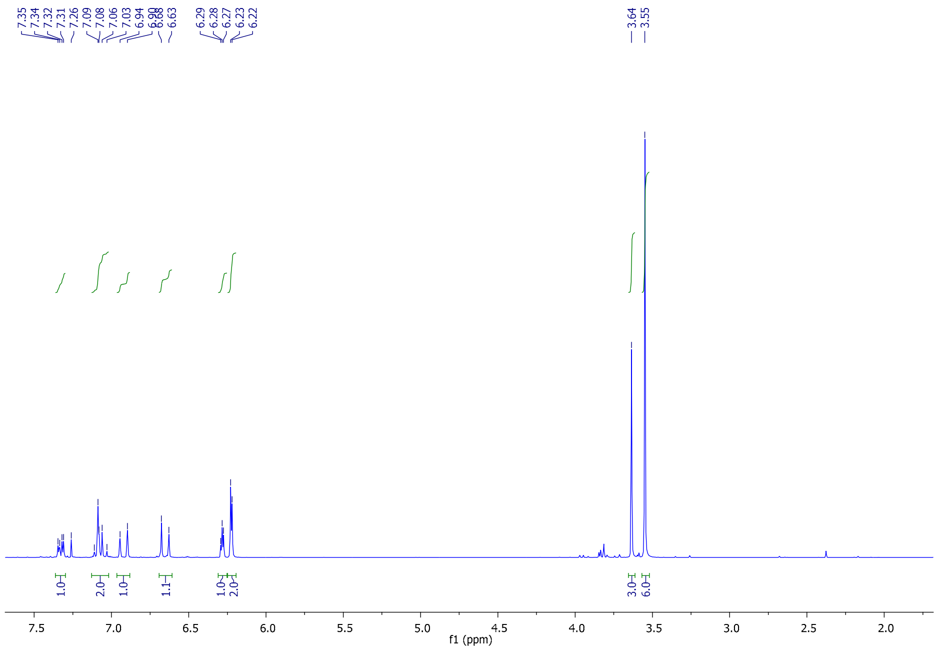

Figure S 4.3.1. ^{1}H-NMR ($CDCl_3$): (*Z*)-4-(3,5-Dimethoxystyryl)-3-methyl-2(3*H*)-benzothiazolone (**17Z**)

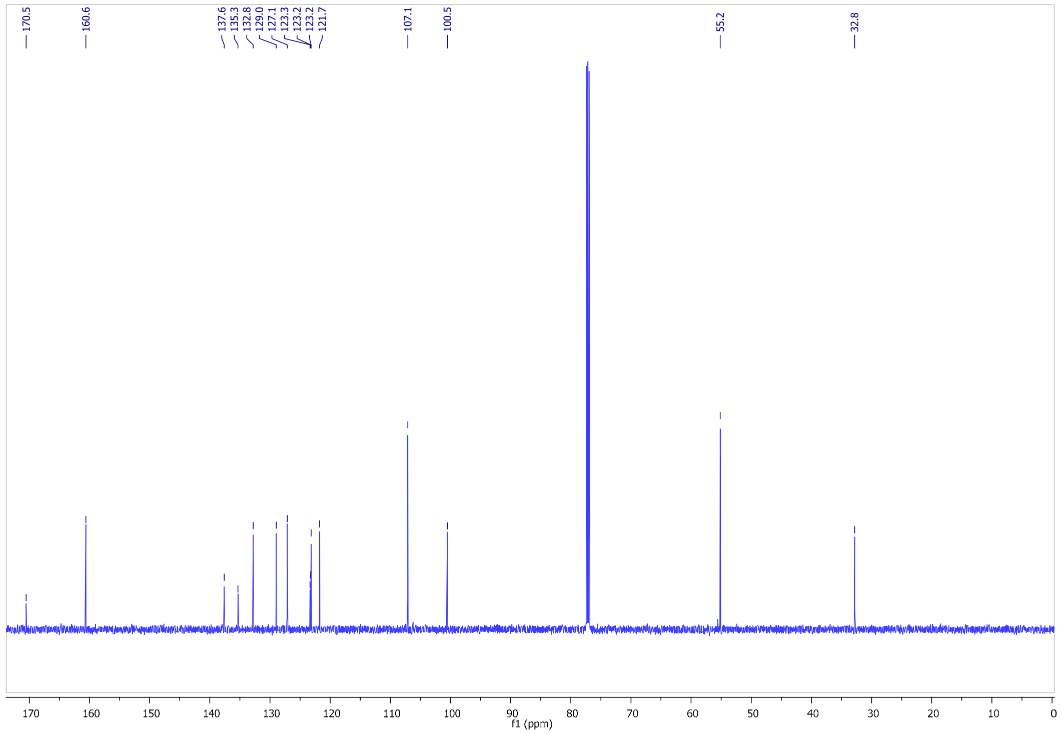

Figure S 4.3.2. ^{13}C-NMR ($CDCl_3$): (*Z*)-4-(3,5-Dimethoxystyryl)-3-methyl-2(3*H*)-benzothiazolone (**17Z**)

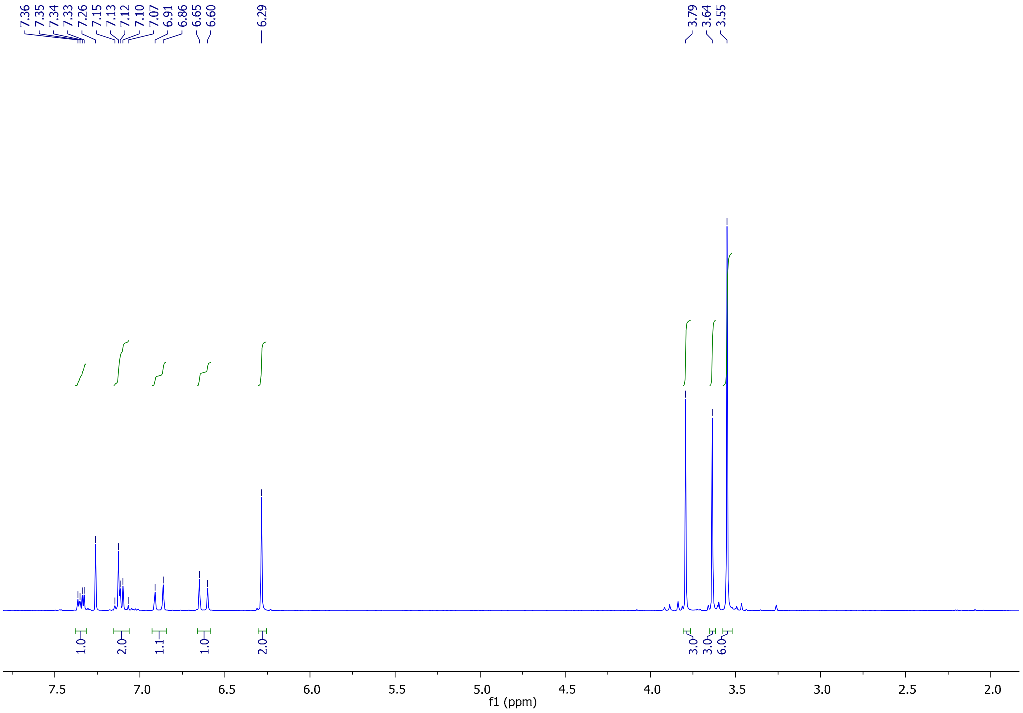

Figure S 4.4.1. ^{1}H-NMR ($CDCl_3$): (*Z*)-3-Methyl-4-(3,4,5-trimethoxystyryl)-2(3*H*)-benzothiazolone (**18Z**)

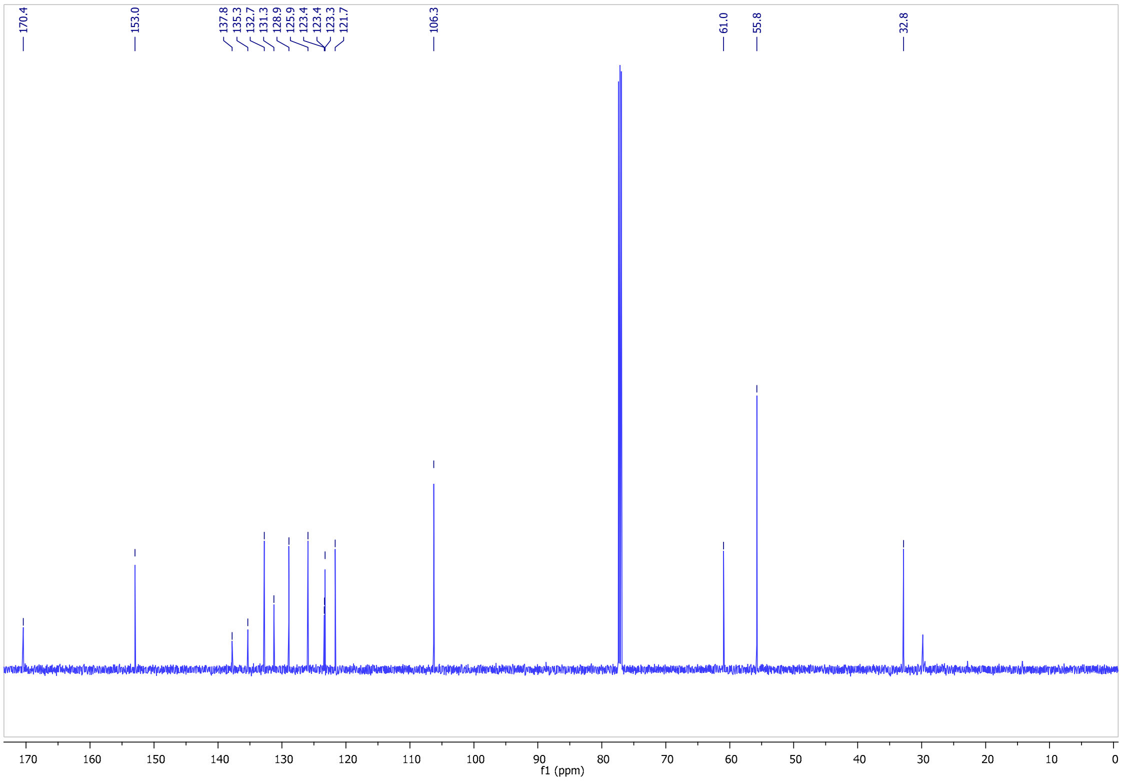

Figure S 4.4.2. ^{13}C-NMR ($CDCl_3$): (*Z*)-3-Methyl-4-(3,4,5-trimethoxystyryl)-2(3*H*)-benzothiazolone (**18Z**)

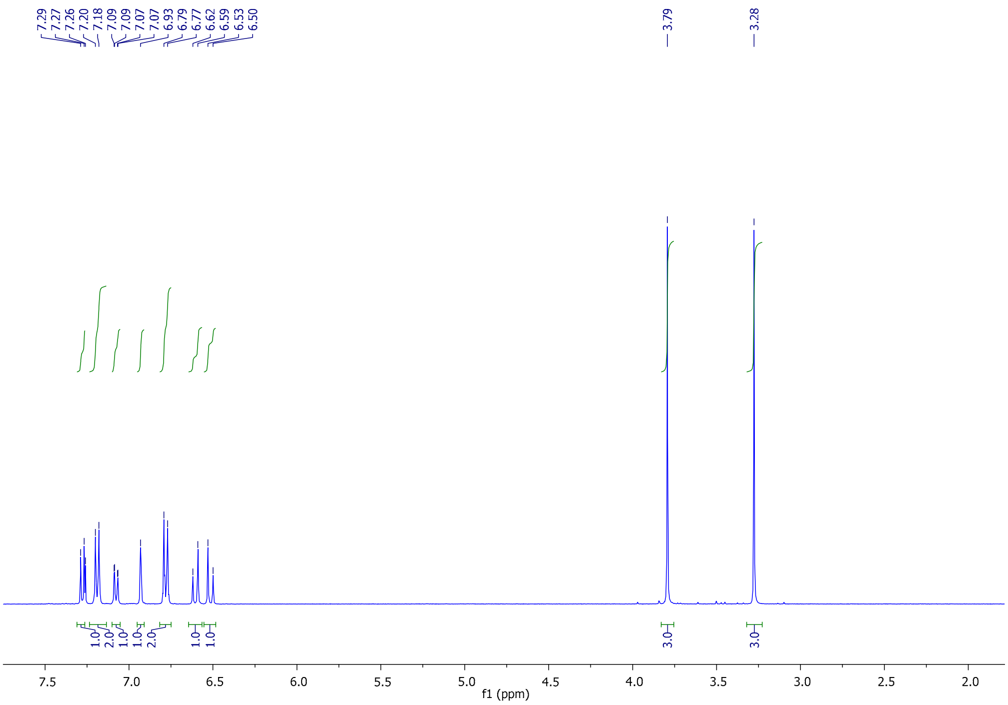

Figure S 4.5.1. ^{1}H-NMR (CDCl$_3$): (*Z*)-5-(4-Methoxystyryl)-3-methyl-2(3*H*)-benzothiazolone (**19Z**)

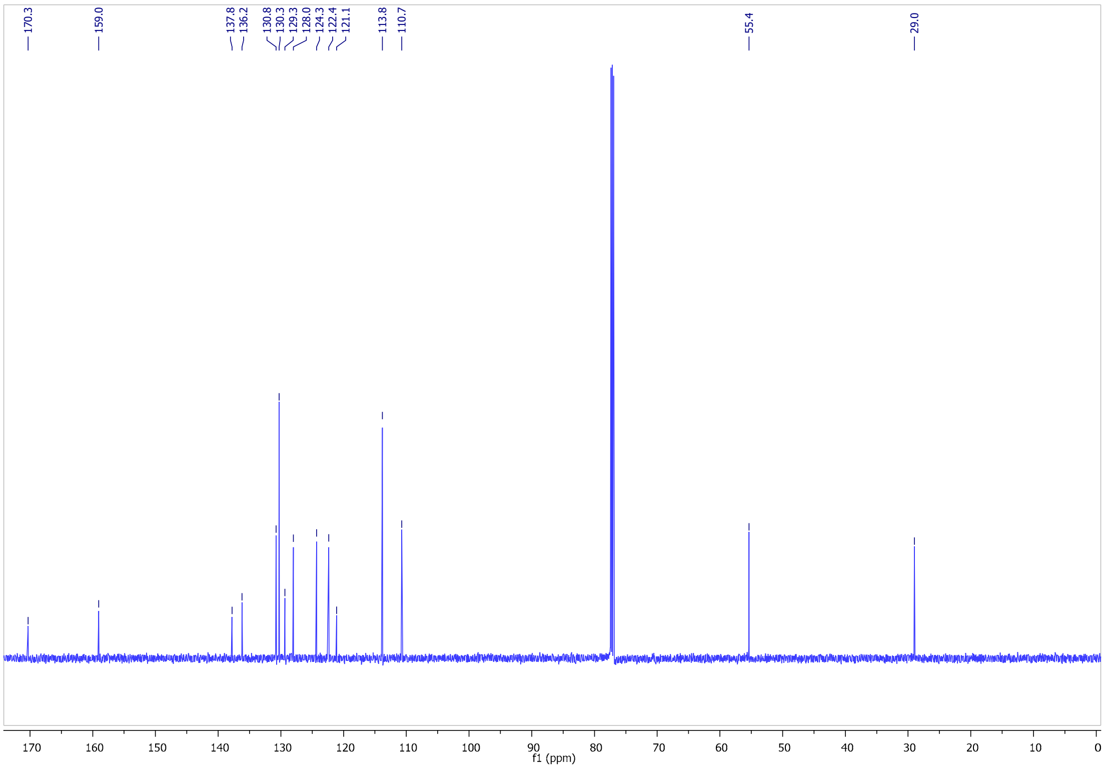

Figure S 4.5.2. ^{13}C-NMR (CDCl$_3$): (*Z*)-5-(4-Methoxystyryl)-3-methyl-2(3*H*)-benzothiazolone (**19Z**)

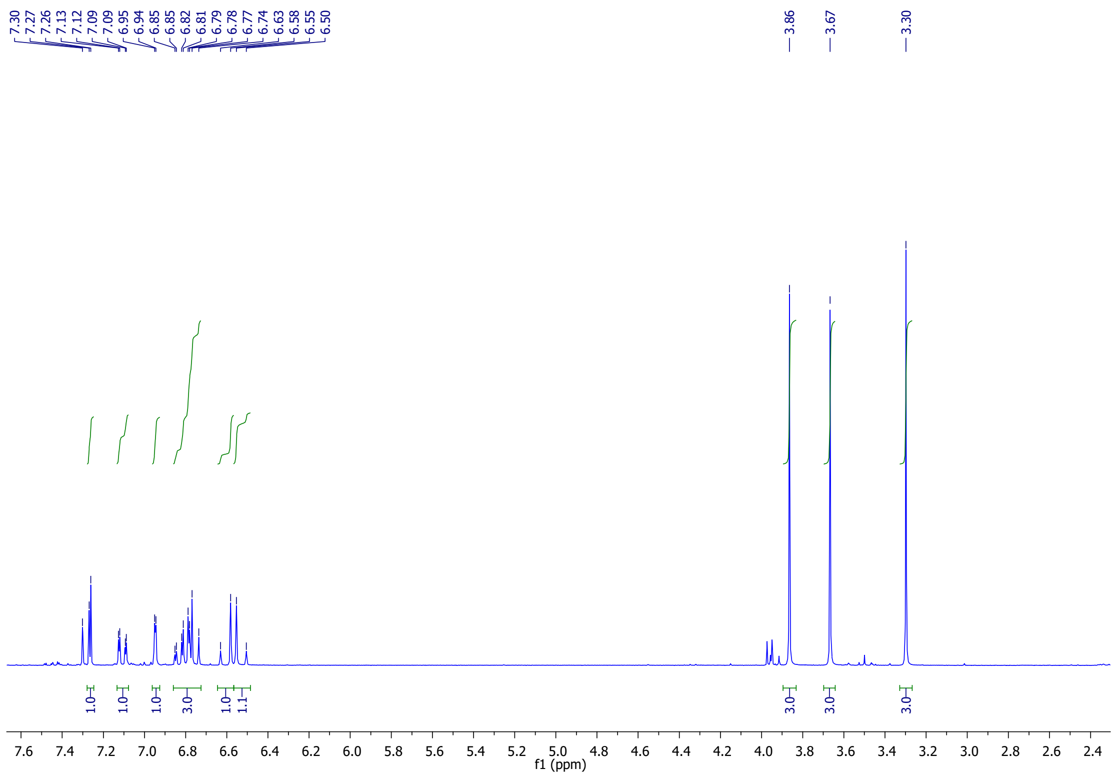

Figure S 4.6.1. ^{1}H-NMR ($CDCl_3$): (*Z*)-5-(3,4-Dimethoxystyryl)-3-methyl-2(3*H*)-benzothiazolone (**20Z**)

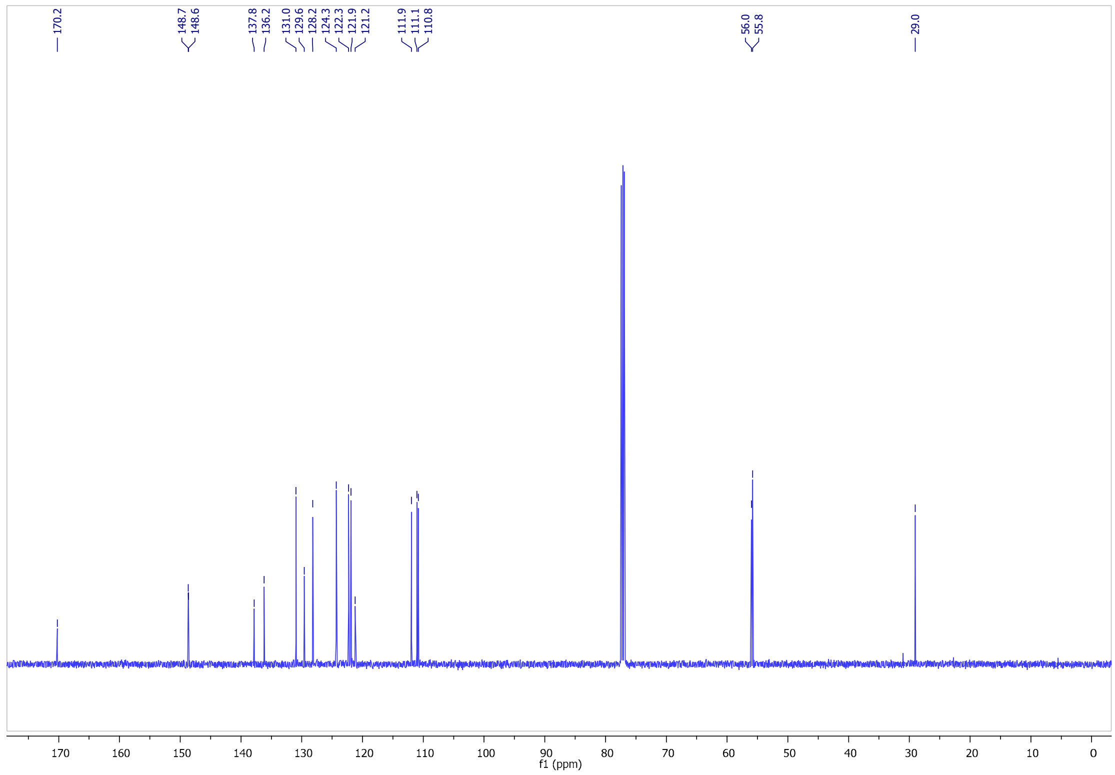

Figure S 4.6.2. ^{13}C-NMR ($CDCl_3$): (*Z*)-5-(3,4-Dimethoxystyryl)-3-methyl-2(3*H*)-benzothiazolone (**20Z**)

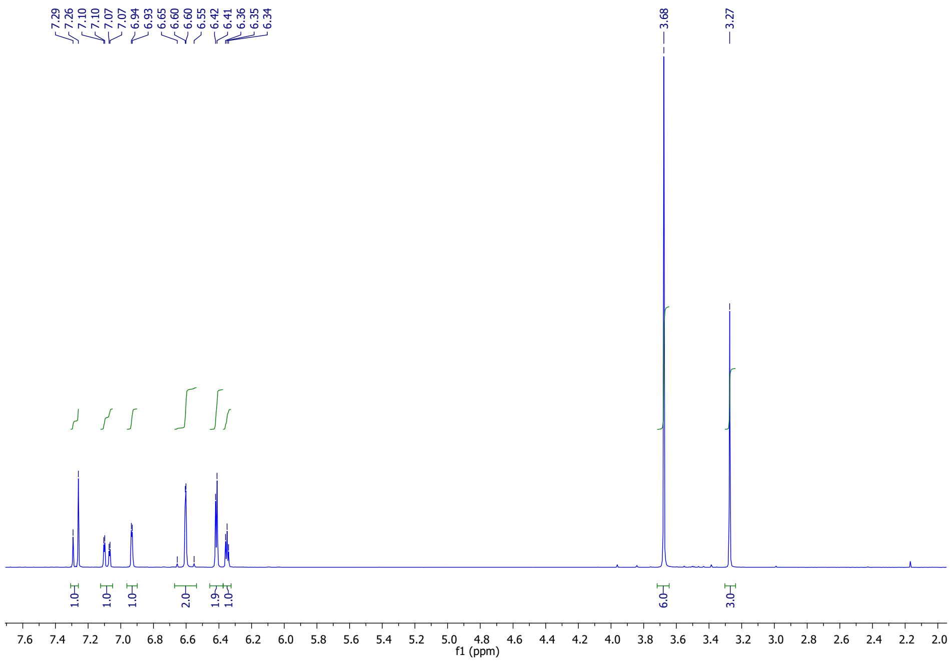

Figure S 4.7.1. ^{1}H-NMR ($CDCl_3$): (*Z*)-5-(3,5-Dimethoxystyryl)-3-methyl-2(3*H*)-benzothiazolone (**21Z**)

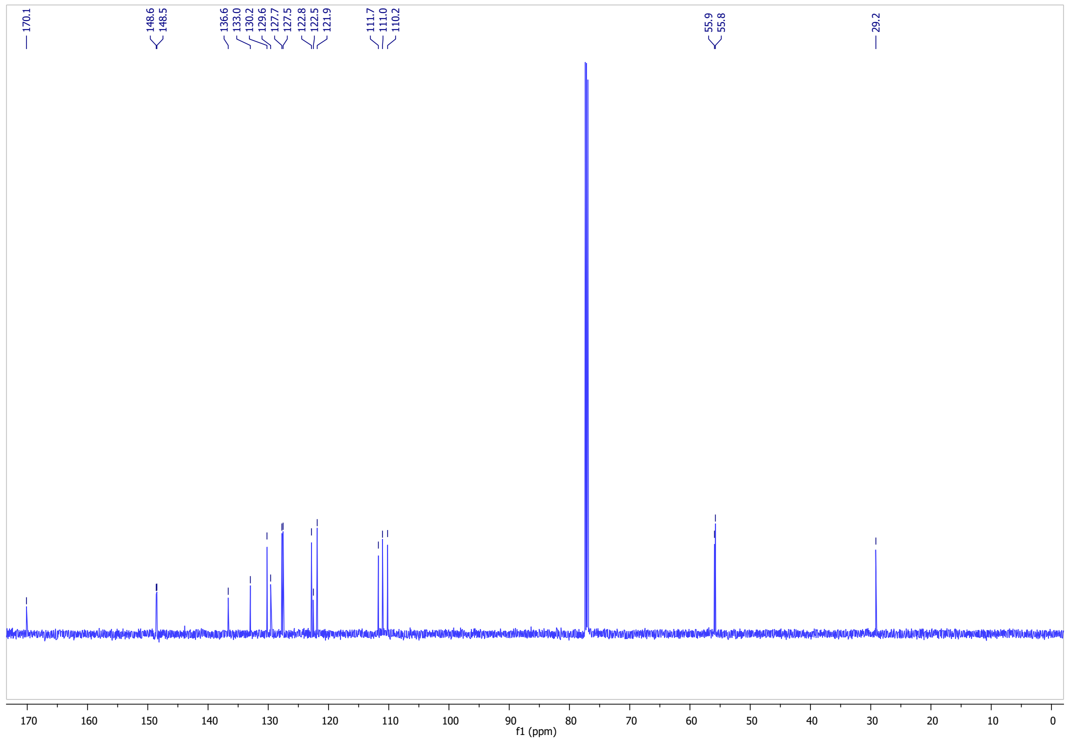

Figure S 4.7.2. ^{13}C-NMR ($CDCl_3$): (*Z*)-5-(3,5-Dimethoxystyryl)-3-methyl-2(3*H*)-benzothiazolone (**21Z**)

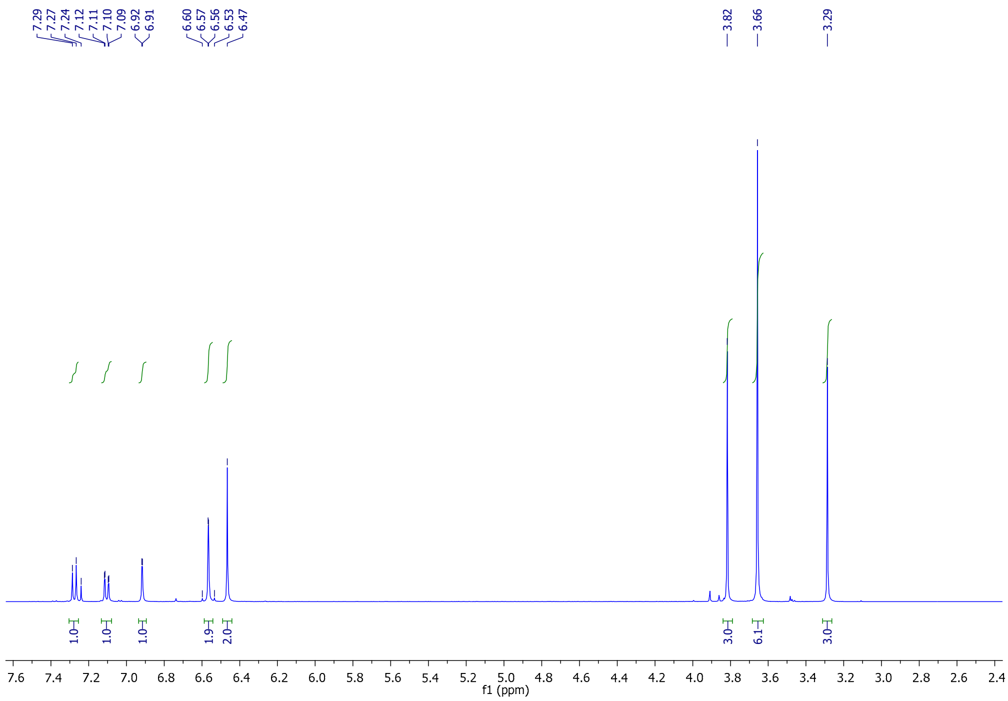

Figure S 4.8.1. ^{1}H-NMR (CDCl$_3$): (*Z*)-3-Methyl-5-(3,4,5-trimethoxystyryl)-2(3*H*)-benzothiazolone (**22Z**)

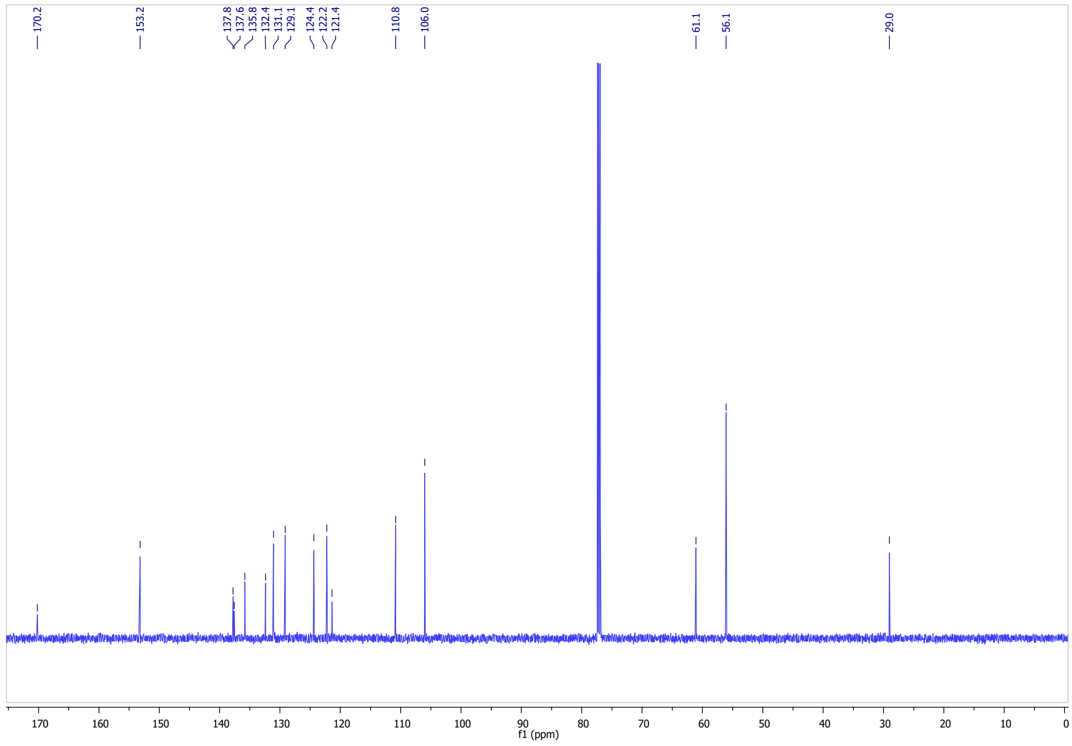

Figure S 4.8.2. ^{13}C-NMR (CDCl$_3$): (*Z*)-3-Methyl-5-(3,4,5-trimethoxystyryl)-2(3*H*)-benzothiazolone (**22Z**)

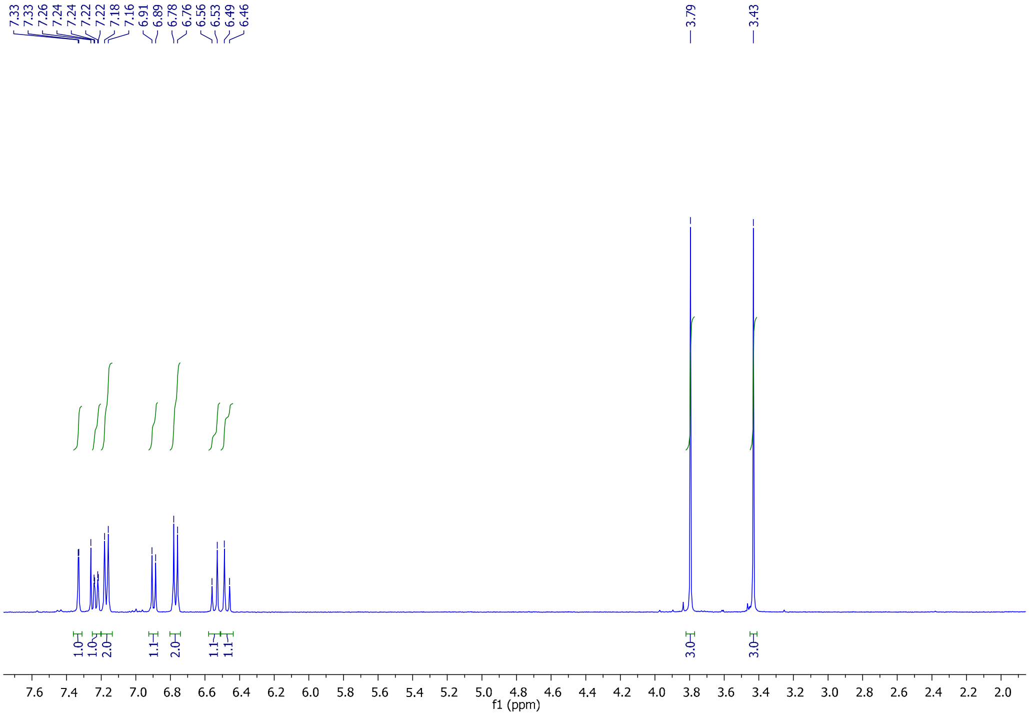

Figure S 4.9.1. ^{1}H-NMR ($CDCl_3$): (*Z*)-6-(4-Methoxystyryl)-3-methyl-2(3*H*)-benzothiazolone (**23Z**)

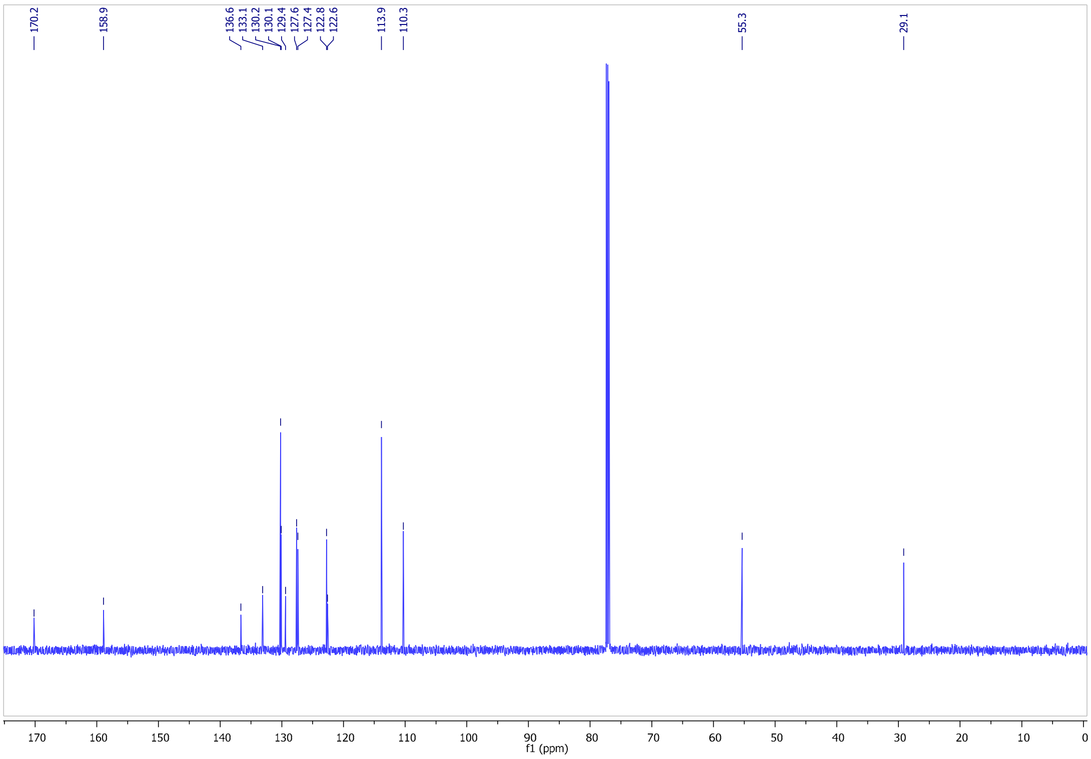

Figure S 4.9.2. ^{13}C-NMR ($CDCl_3$): (*Z*)-6-(4-Methoxystyryl)-3-methyl-2(3*H*)-benzothiazolone (**23Z**)

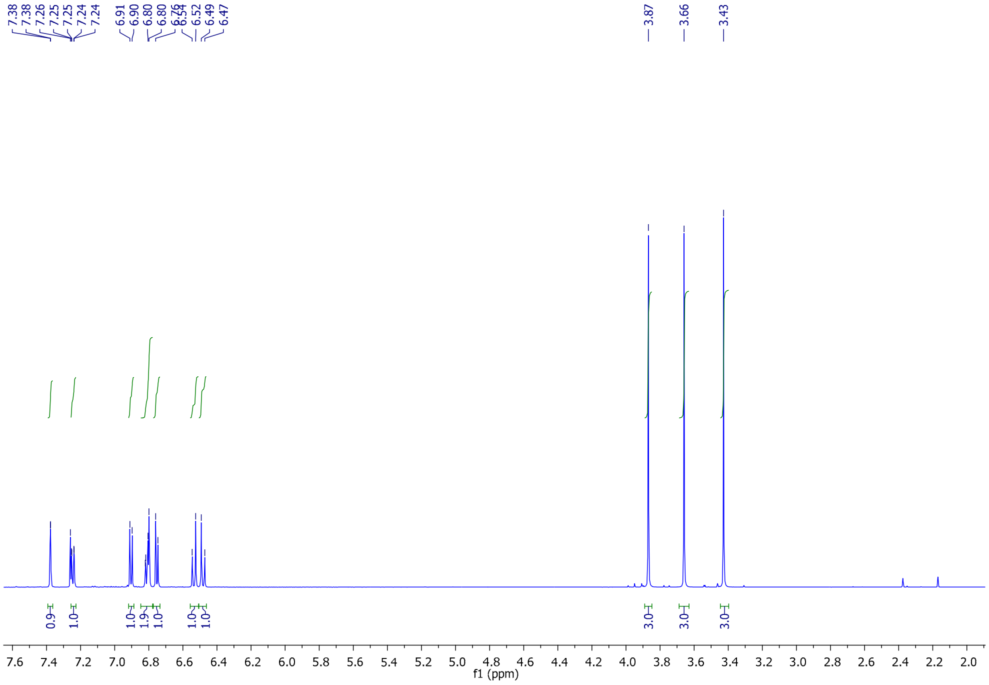

Figure S 4.10.1. ^{1}H-NMR ($CDCl_3$): (*Z*)-6-(3,4-Dimethoxystyryl)-3-methyl-2(3*H*)-benzothiazolone (**24Z**)

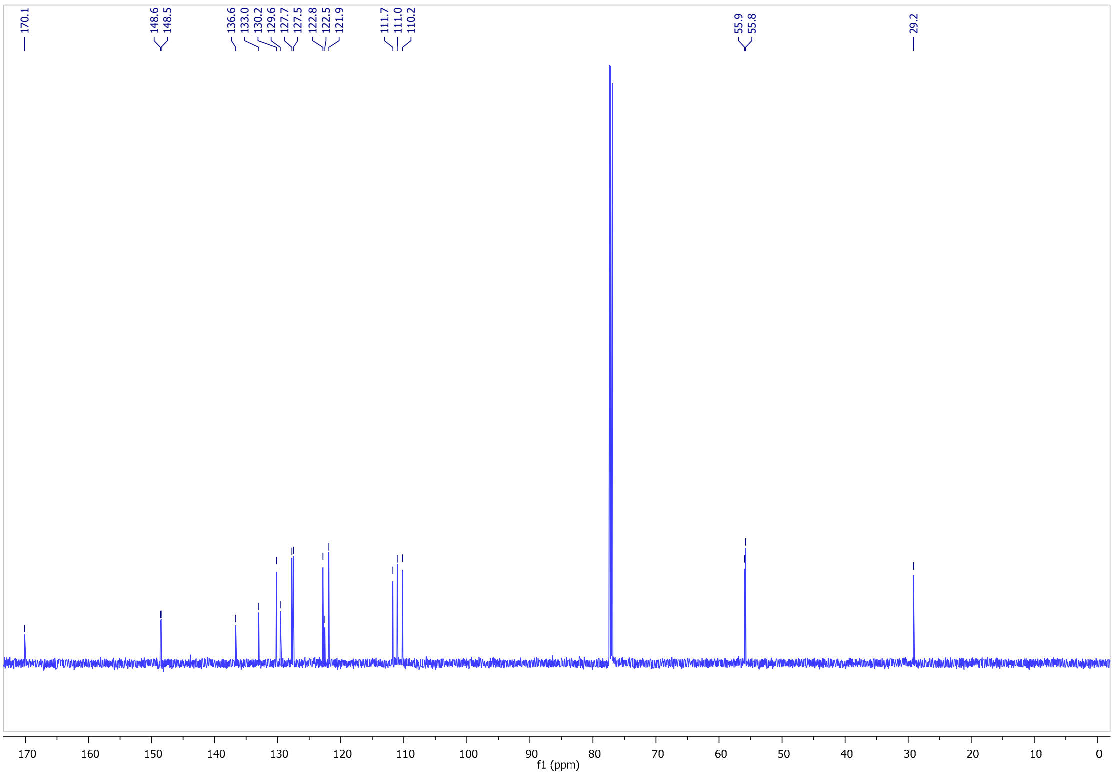

Figure S 4.10.2. ^{13}C-NMR ($CDCl_3$): (*Z*)-6-(3,4-Dimethoxystyryl)-3-methyl-2(3*H*)-benzothiazolone (**24Z**)

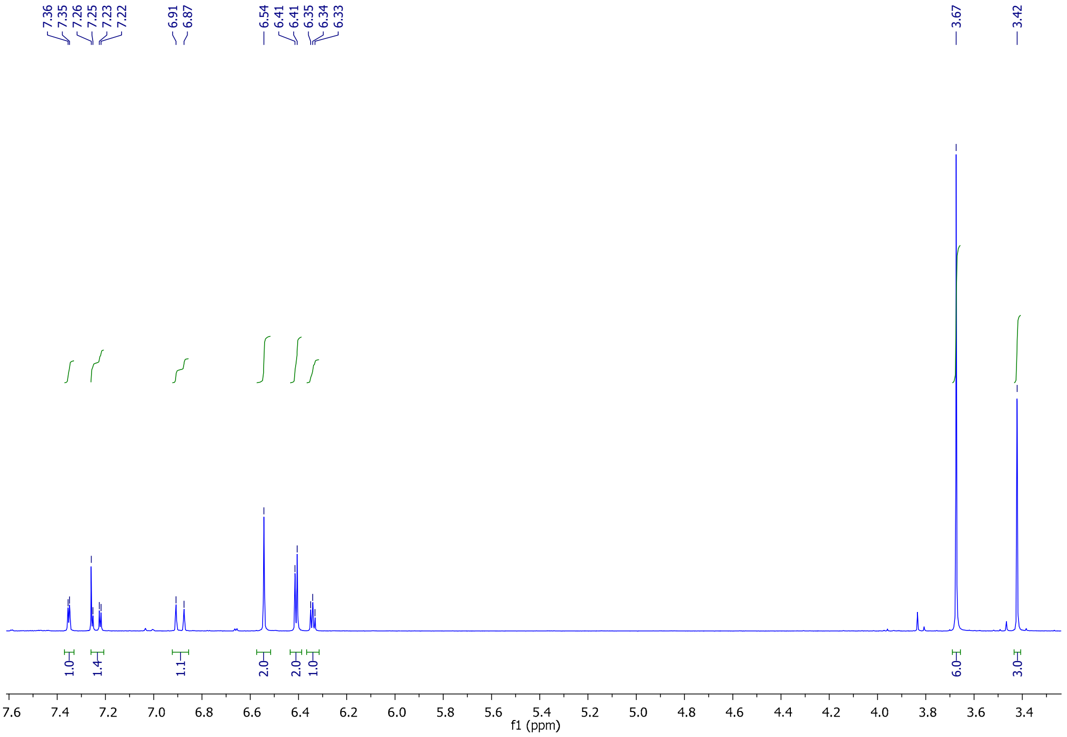

Figure S 4.11.1. ^{1}H-NMR ($CDCl_3$): (*Z*)-6-(3,5-Dimethoxystyryl)-3-methyl-2(3*H*)-benzothiazolone (**25Z**)

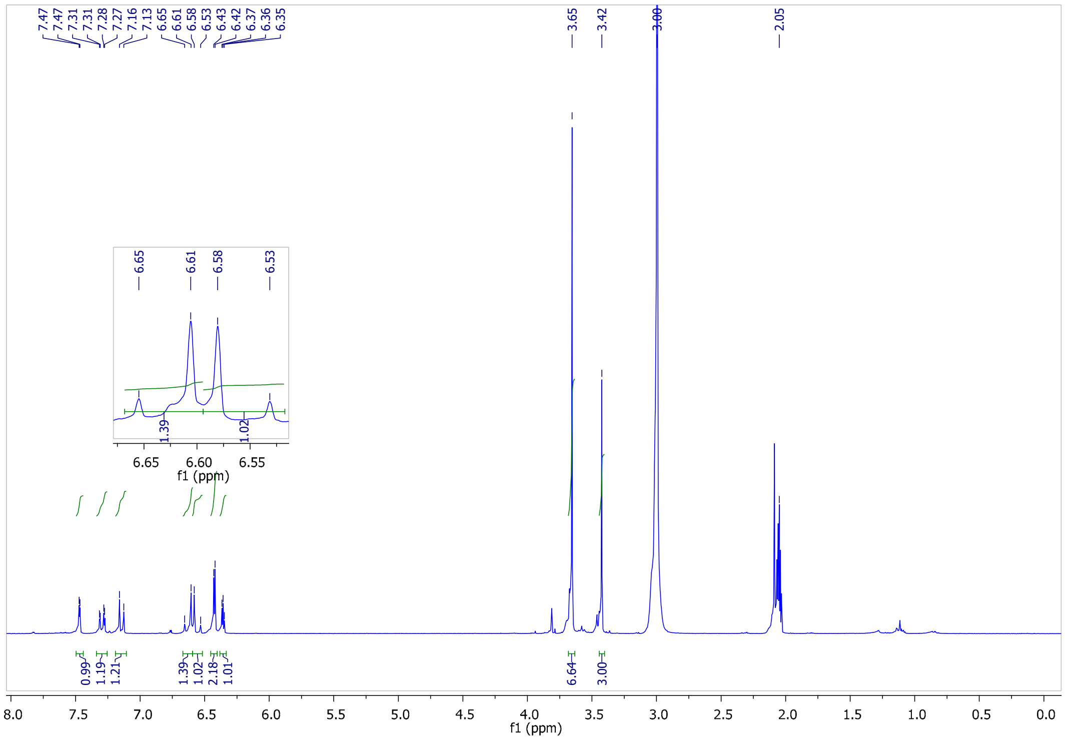

Figure S 4.11.2. ^{1}H-NMR (acetone-d_6): (*Z*)-6-(3,5-Dimethoxystyryl)-3-methyl-2(3*H*)-benzothiazolone (**25Z**)

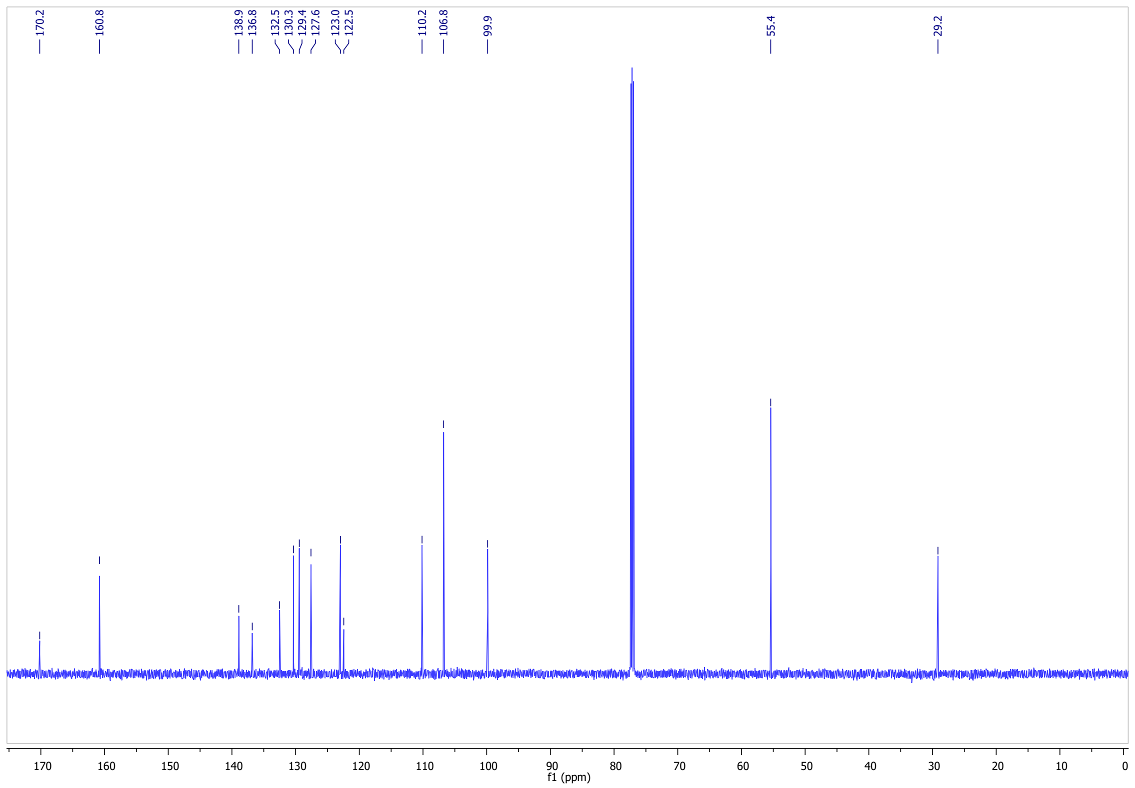

Figure S 4.11.3. ^{13}C-NMR ($CDCl_3$): (*Z*)-6-(3,5-Dimethoxystyryl)-3-methyl-2(3*H*)-benzothiazolone (**25Z**)

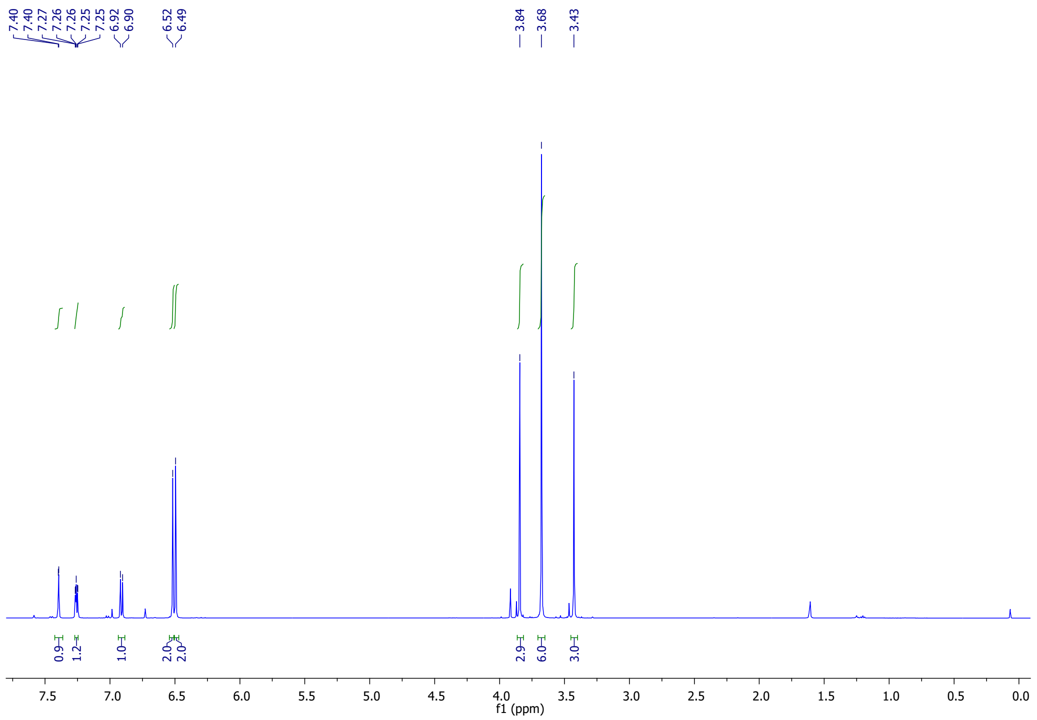

Figure S 4.12.1. ^{1}H-NMR ($CDCl_3$): (*Z*)-3-Methyl-6-(3,4,5-trimethoxystyryl)-2(3*H*)-benzothiazolone (**26Z**)

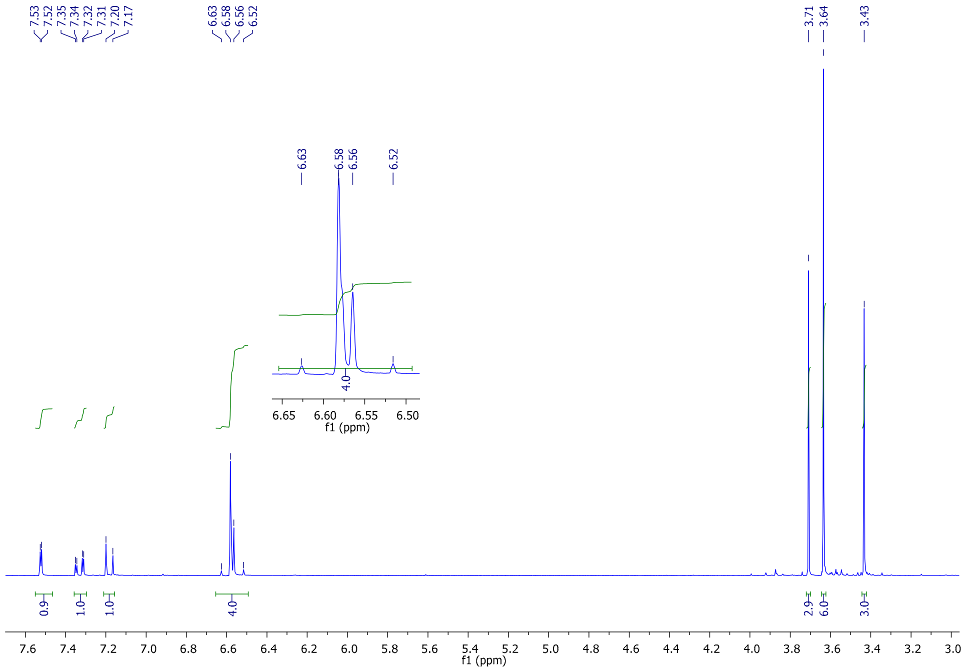

Figure S 4.12.2. ^{1}H-NMR (acetone-d$_6$): (*Z*)-3-Methyl-6-(3,4,5-trimethoxystyryl)-2(3*H*)-benzothiazolone (**26Z**)

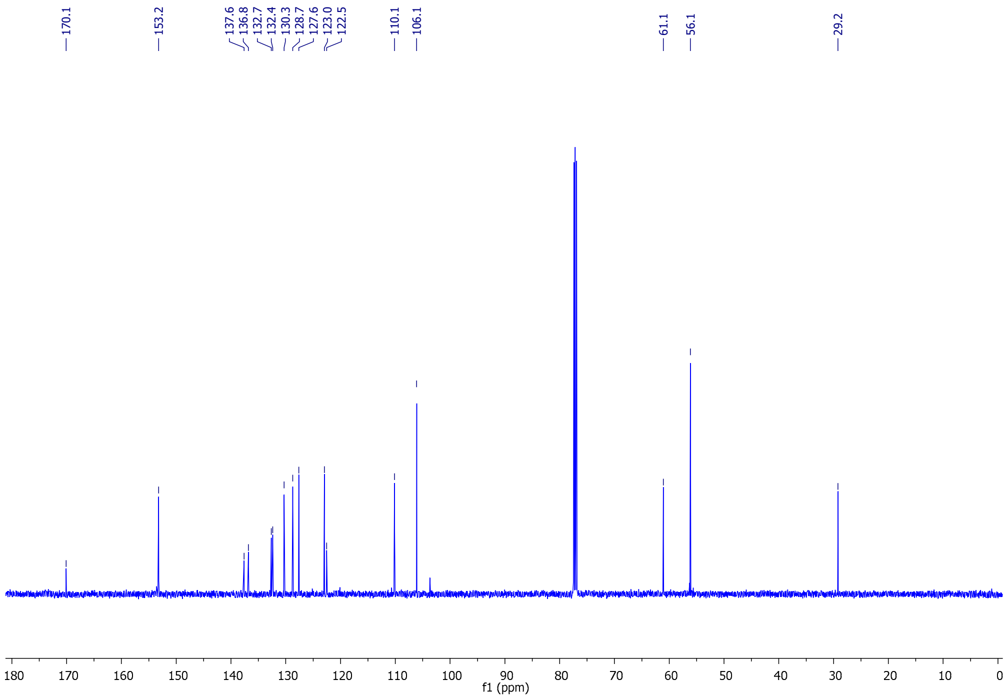

Figure S 4.12.3. ^{13}C-NMR (CDCl$_3$): (*Z*)-3-Methyl-6-(3,4,5-trimethoxystyryl)-2(3*H*)-benzothiazolone (**26Z**)

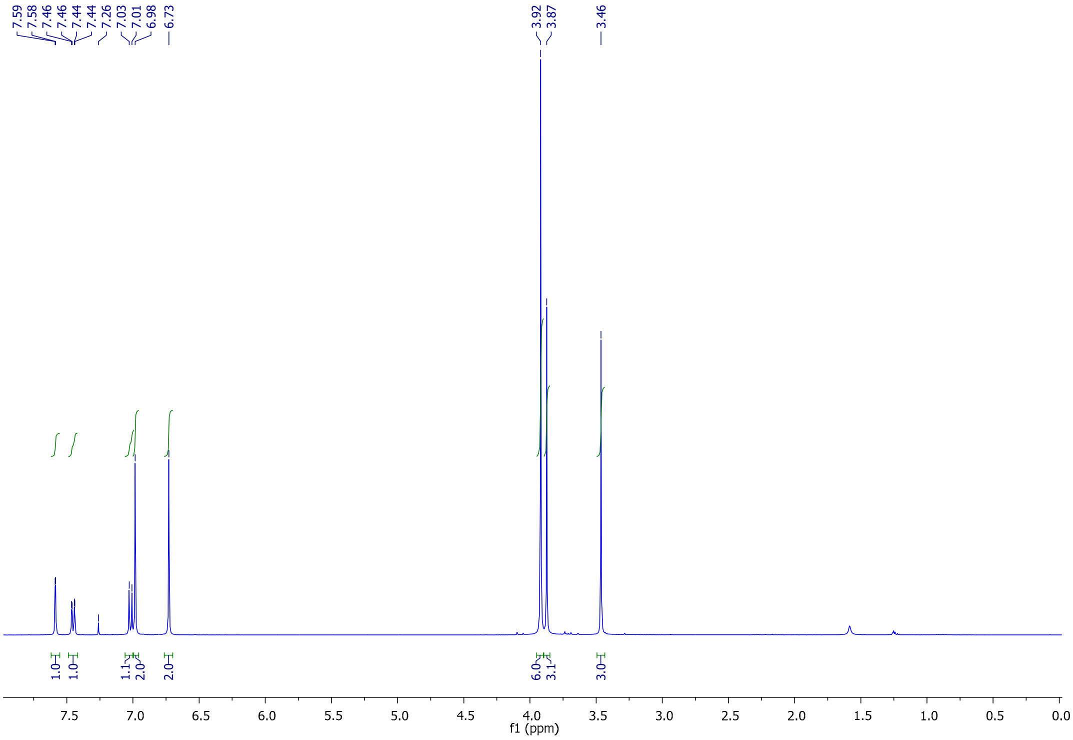

Figure S 4.13.1. ^{1}H-NMR ($CDCl_3$): (*E*)-3-Methyl-6-(3,4,5-trimethoxystyryl)-2(3*H*)-benzothiazolone (**26E**)

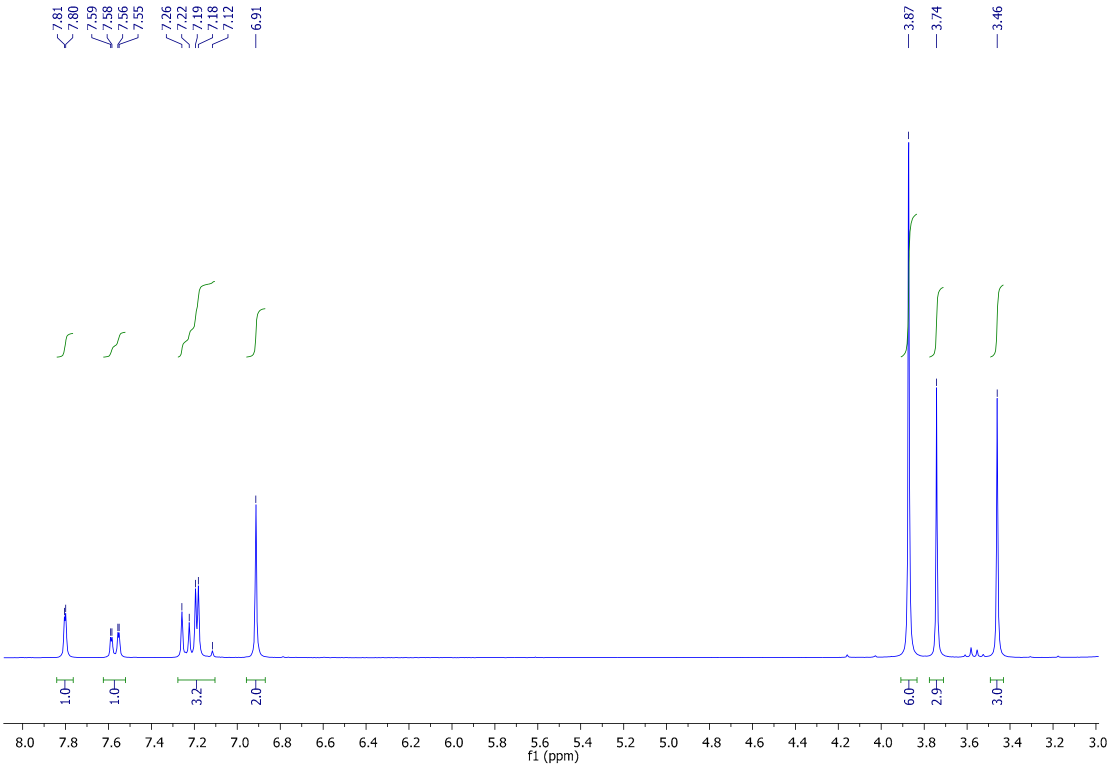

Figure S 4.13.2. ^{1}H-NMR (acetone-d_6): (*E*)-3-Methyl-6-(3,4,5-trimethoxystyryl)-2(3*H*)-benzothiazolone (**26E**)

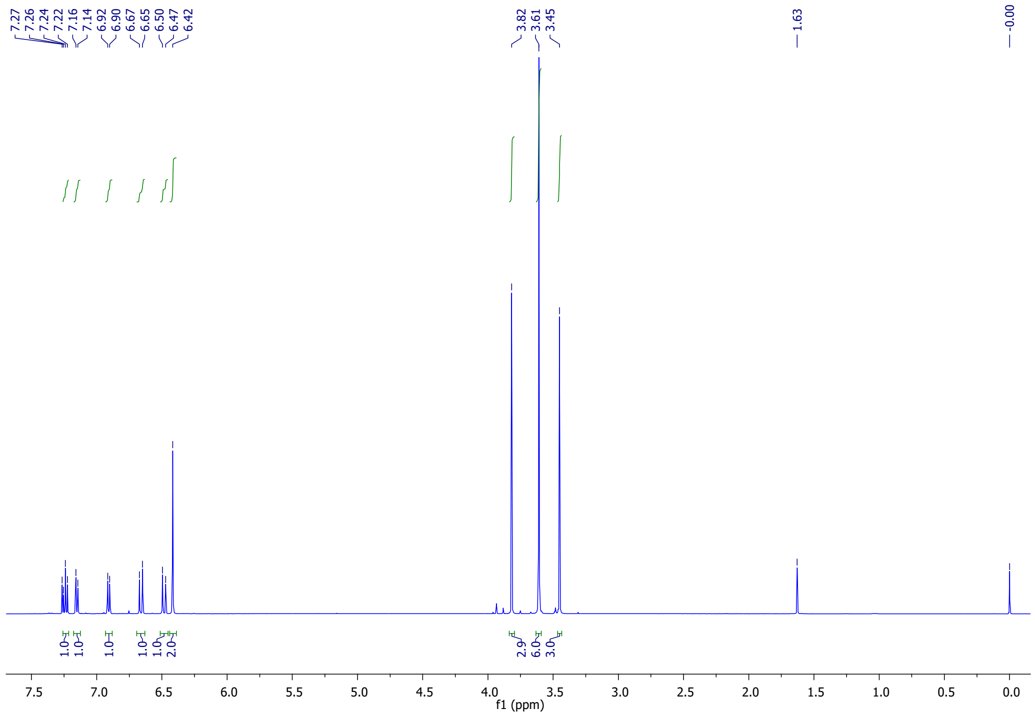

Figure S 4.14.1. ¹H-NMR ($CDCl_3$): (*Z*)-3-Methyl-7-(3,4,5-trimethoxystyryl)-2(3*H*)-benzothiazolone (**27Z**)

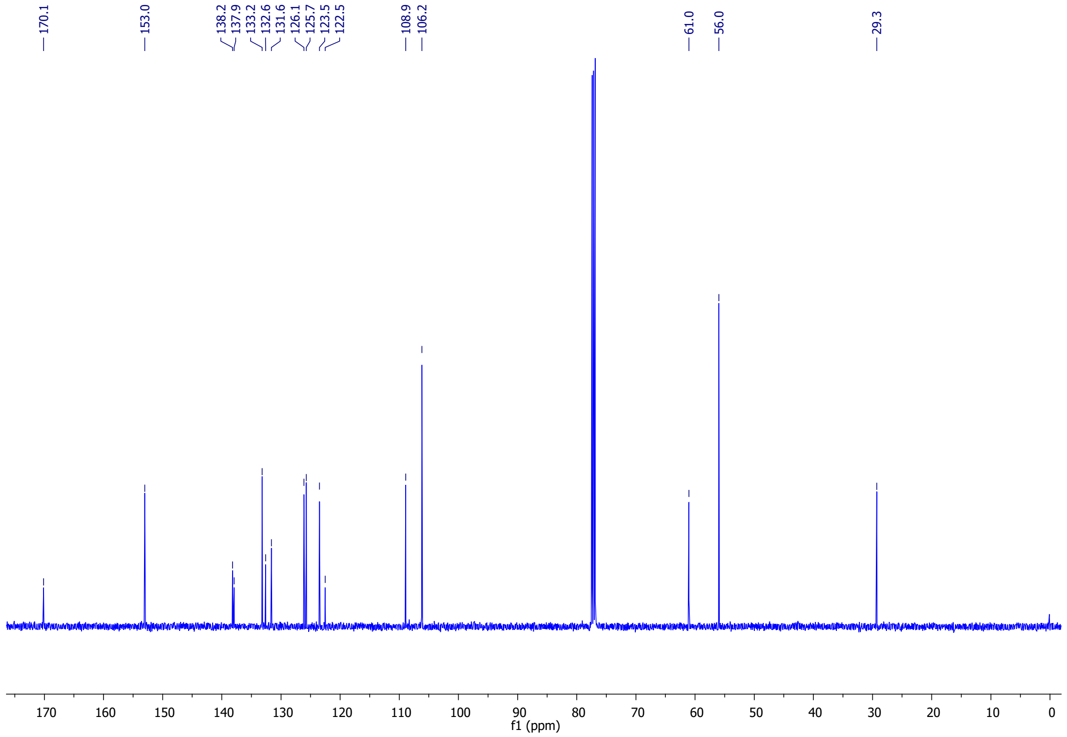

Figure S 4.14.2. ¹³C-NMR ($CDCl_3$): (*Z*)-3-Methyl-7-(3,4,5-trimethoxystyryl)-2(3*H*)-benzothiazolone (**27Z**)

References

[1] M.S. Gerova, F.E. Svetoslavov, B.L. Shivachev, R.P. Nikolova, O.I. Petrov, Synthesis of 4-acetyl-2(3*H*)-benzothiazolone: Sulfur bioisostere of benzoxazolone allelochemicals, Phosphorus Sulfur Silicon Relat. Elem, 192 (2017) 905-910.
[2] B. Zhou, H. Hong, H. Wang, T. Zhang, L. Han, N. Zhu, Efficient synthesis of benzothiazolone derivatives by a domino reaction of disulfide and COS under mild conditions, Eur. J. Org. Chem, (2018) 6983-6990.
[3] J. Teppema, B. Sebrell, Researches on mercaptothiazoles. I, J. Am. Chem. Soc, 49 (1927) 1748-1758.
[4] D.V. Dekhane, S. S. Pawar, S. V. Gupta, M. S. Shingare, S. N. Thore, Synthesis of benzimidazolones, benzooxazolones, 2-amino-benzothiazoles from ethyl cyanoformate and o-phenylene diamines, o-aminophenols, oaminothiophenols promoted by lithium bromide, Lett. Org. Chem.,8 (2011) 406-411.
[5] C. Franchini, M. Muraglia, F. Corbo, M.A. Florio, A. Di Mola, A. Rosato, R. Matucci, M. Nesi, F. van Bambeke, C. Vitali, Synthesis and biological evaluation of 2-mercapto-1,3-benzothiazole derivatives with potential antimicrobial activity, Arch. Pharm, 342 (2009) 605-613.
[6] R.O. Matevosyan, J. Org. Chem. USSR in english translation, 4 (1968) 1405-1408.
[7] P. Hrobárik, V. Hrobáriková, I. Sigmundová, P. Zahradník, M. Fakis, I. Polyzos, P. Persephonis, Benzothiazoles with tunable electron-withdrawing strength and reverse polarity: a route to triphenylamine-based chromophores with enhanced two-photon absorption, The J. Org. Chem, 76 (2011) 8726-8736.
[8] F.R. Hunter, R. E. Parken, The unsaturation and tautomeric mobility of heterocyclic compounds. Part VI. The mobility of 5-substituted 1-hydroxybenzthiazoles, and the ultra-violet absorption of mobile and static derivatives of 1-hydroxybenzthiazole, J. Org. Chem (1935) 1755-1761.
